# Identification of dendritic cell precursor from the CD11c^+^ cells expressing high levels of MHC class II molecules in the culture of bone marrow with FLT3 ligand

**DOI:** 10.3389/fimmu.2023.1179981

**Published:** 2023-11-29

**Authors:** Hyunju In, Ji Soo Park, Hyun Soo Shin, Seul Hye Ryu, Moah Sohn, Wanho Choi, Sejung Park, Soomin Hwang, Jeyun Park, Lihua Che, Tae-Gyun Kim, Min Kyung Chu, Hye Young Na, Chae Gyu Park

**Affiliations:** ^1^ Laboratory of Immunology, Severance Biomedical Science Institute, Yonsei University College of Medicine, Seoul, Republic of Korea; ^2^ Brain Korea 21 PLUS/FOUR Project for Medical Science, Yonsei University College of Medicine, Seoul, Republic of Korea; ^3^ Heart-Immune-Brain Network Research Center, Department of Life Science, Ewha Womans University, Seoul, Republic of Korea; ^4^ Department of Dermatology, Severance Hospital, Cutaneous Biology Research Institute, Yonsei University College of Medicine, Seoul, Republic of Korea; ^5^ Department of Neurology, Severance Hospital, Yonsei University College of Medicine, Seoul, Republic of Korea; ^6^ Laboratory of Dendritic Cell Immunology, The Good Capital Institute for Immunology, Seoul, Republic of Korea

**Keywords:** antigen presentation, bone marrow cells, cell differentiation, cultured cells, dendritic cells, FLT3 ligand, GM-CSF, precursor cells

## Abstract

Dendritic cells (DCs) are readily generated from the culture of mouse bone marrow (BM) treated with either granulocyte macrophage-colony stimulating factor (GM-CSF) or FMS-like tyrosine kinase 3 ligand (FLT3L). CD11c^+^MHCII^+^ or CD11c^+^MHCII^hi^ cells are routinely isolated from those BM cultures and generally used as *in vitro*-generated DCs for a variety of experiments and therapies. Here, we examined CD11c^+^ cells in the BM culture with GM-CSF or FLT3L by staining with a monoclonal antibody 2A1 that is known to recognize mature or activated DCs. Most of the cells within the CD11c^+^MHCII^hi^ DC gate were 2A1^+^ in the BM culture with GM-CSF (GM-BM culture). In the BM culture with FLT3L (FL-BM culture), almost of all the CD11c^+^MHCII^hi^ cells were within the classical DC2 (cDC2) gate. The analysis of FL-BM culture revealed that a majority of cDC2-gated CD11c^+^MHCII^hi^ cells exhibited a 2A1^-^CD83^-^CD115^+^CX_3_CR1^+^ phenotype, and the others consisted of 2A1^+^CD83^+^CD115^-^CX_3_CR1^-^ and 2A1^-^CD83^-^CD115^-^CX_3_CR1^-^ cells. According to the antigen uptake and presentation, morphologies, and gene expression profiles, 2A1^-^CD83^-^CD115^-^CX_3_CR1^-^ cells were immature cDC2s and 2A1^+^CD83^+^CD115^-^CX_3_CR1^-^ cells were mature cDC2s. Unexpectedly, however, 2A1^-^CD83^-^CD115^+^CX_3_CR1^+^ cells, the most abundant cDC2-gated MHCII^hi^ cell subset in FL-BM culture, were non-DCs. Adoptive cell transfer experiments in the FL-BM culture confirmed that the cDC2-gated MHCII^hi^ non-DCs were precursors to cDC2s, i.e., MHCII^hi^ pre-cDC2s. MHCII^hi^ pre-cDC2s also expressed the higher level of DC-specific transcription factor Zbtb46 as similarly as immature cDC2s. Besides, MHCII^hi^ pre-cDC2s were generated only from pre-cDCs and common DC progenitor (CDP) cells but not from monocytes and common monocyte progenitor (cMoP) cells, verifying that MHCII^hi^ pre-cDC2s are close lineage to cDCs. All in all, our study identified and characterized a new cDC precursor, exhibiting a CD11c^+^MHCII^hi^CD115^+^CX_3_CR1^+^ phenotype, in FL-BM culture.

## Introduction

Antigen presenting cells (APCs) such as dendritic cells (DCs) and macrophages (Macs) are important players mediating innate3and adaptive immunity. Particularly, DCs are potent professional APCs that can stimulate naive T cells to proliferate and differentiate efficiently (1–3). Classical DCs (cDCs) are derived from common DC progenitor (CDP) cells via pre-cDCs, while plasmacytoid DCs (pDCs) develop from both CDPs and common lymphoid progenitor (CLP) cells (4). CDPs and common monocyte progenitor (cMoP) cells are derived from monocyte and DC progenitor (MDP) cells (5, 6). Monocytes, originating from cMoPs, can also be converted into DCs, i.e., monocyte-derived DCs (Mo-DCs) by *in vitro* treatment with granulocyte macrophage-colony stimulating factor (GM-CSF) and IL-4 as well as during inflammatory conditions *in vivo* (7).

CD11c^+^ DCs efficiently develop after culture of bone marrow (BM) with GM-CSF or FLT3 ligand (FLT3L) for 6 to 10 days (8–10), which has been widely used as *in vitro*-generated BM-derived DCs (BM-DCs) in various experiments. Apparently, however, not all the CD11c^+^ cells generated from those BM cultures are a homogeneous population. In the BM culture with GM-CSF (GM-BM culture), DCs and Macs are abundantly found among the CD11c^+^ cells. While MDPs, cMoPs, and monocytes differentiate into both DCs and Macs, CDPs preferentially generate DCs in GM-BM culture (11). Meanwhile, in the CD11c^+^ cells generated from the BM culture with FLT3L (FL-BM culture), a variety of DC subsets such as pDCs, classical type 1 DCs (cDC1s) and cDC2s are found (10).

2A1 is one of the monoclonal antibodies (mAbs) generated by early efforts to produce new reagents that specifically detected DCs in GM-BM culture as well as in lymphoid tissues *in situ* (8, 12–15). The cells detected by mAb 2A1, i.e., 2A1^+^ cells are abundantly present in the T cell rich regions of lymph nodes, spleen, and Peyer’s patch, suggesting that 2A1 marks a subset of DCs closely located near T cells in lymphoid tissues (16–18). Analysis of GM-BM culture also revealed that only the MHCII^hi^ cells were detected by mAb 2A1 (8, 14). Based on those observations, 2A1^+^ DCs have been considered as activated or mature DCs (19). However, unlike other well-characterized surface markers for DCs, publications examining 2A1^+^ DCs have been rare because mAb 2A1 recognizes an unknown antigen localized intracellularly in DCs. As a result, the function of 2A1 antigen (hereafter 2A1) in DCs is still unknown.

Here, we set out to examine the expression of 2A1 in CD11c^+^ cells from GM-BM and FL-BM cultures. All the DCs in GM-BM culture (GM-DCs) highly expressed 2A1, whereas only small fractions of cDC-gated cell subsets in FL-BM culture were 2A1^+^. In FL-BM culture, most of those 2A1^+^ cells were found within the cells gated for cDC2 subset expressing high levels of MHC class II molecules (MHCII^hi^), and more than a half of CD11c^+^MHCII^hi^ cDC2-gated cells were 2A1^-^. Up to now, together with GM-DCs, CD11c^+^MHCII^+^ cells in FL-BM culture have been widely used as *in vitro*-generated DCs (20–22). Surprisingly, however, we found only a half of the CD11c^+^MHCII^hi^ cells in FL-BM culture were DCs. The remaining half of the CD11c^+^MHCII^hi^ cells in FL-BM culture were not DCs but DC precursor cells which were poor at antigen presentation. In this report, we identified and characterized a novel DC precursor population, which displayed a CD11c^+^MHCII^hi^CD115^+^CX_3_CR1^+^ phenotype, in FL-BM culture.

## Materials and methods

### Animals

C57BL/6 mice were purchased from the Orient Bio (Seongnam, Korea). C57BL/6-Tg(TcraTcrb)1100Mjb/J (OT-1), B6.Cg-Tg(TcraTcrb)425Cbn/J (OT-2), B6.SJL-Ptprc^a^Pepc^b^/BoyJ (CD45.1), and B6.129S6(C)-*Zbtb46^tm1.1Kmm^*/J (*Zbtb46*-gfp) mice were obtained from the Jackson Laboratory (Bar Harbor, ME). CD45.1^+^ OT-1 and CD45.1^+^ OT-2 mice were bred in house. Animal care and experiments were carried out according to the guidelines and protocols set and approved by the Institutional Animal Care and Use Committee of the Yonsei University College of Medicine (approval numbers 2016-0040, 2017-0001, 2019-0024, 2019-0180, 2020-0003, 2021-0010).

### Cells, antibodies, and reagents

Chinese hamster ovary (CHO) cells were purchased from GIBCO (CHO-S cells; Thermo Fisher Scientific, Waltham, MA). Cells were cultured in DMC7 medium (23, 24) composed of DMEM containing L-glutamine, high glucose, and pyruvate (HyClone, Logan, UT) supplemented with 7% fetal bovine serum (FBS; HyClone) and 1× solutions of non-essential amino acids (HyClone), antibiotic-antimycotic (HyClone), and primocin (InvivoGen, San Diego, CA). The following fluorochrome-conjugated mAbs were purchased from BioLegend (San Diego, CA): anti-CD3 (17A2), anti-CD4 (GK1.5), anti-CD8a (53-6.7), anti-CD11b (M1/70), anti-CD11c (N418), anti-CD14 (Sa14-2), anti-CD16/32 (93), anti-CD19 (6D5), anti-CD24 (M1/69), anti-CD45.1 (A20), anti-CD45.2 (104), anti-B220/CD45R (RA3-6B2), anti-TCRβ (H57-597), anti-TCR Vα2 (B20.1), anti-CD62L (MEL-14), anti-CD64 (X54-5/7.1), anti-CD80 (16-10A1), anti-CD83 (Michel-19), anti-CD86 (GL-1), anti-CD103 (2E7), anti-CD115 (AFS98), anti-CD117 (2B8), anti-CD135 (A2F10), anti-CD172α (P84), anti-CD205/DEC205 (NLDC145), anti-CD206 (C068C2), anti-CD301a (LOM-8.7), anti-CD301b (URA-1), anti-CCR9 (CW-1.2), anti-PD-1 (2F.1A12), anti-PD-L1 (10F.9G2), anti-PD-L2 (TY25), anti-BTLA (6A6), anti-CCR2 (475301), anti-CCR3 (J073E5), anti-CCR7 (4B12), anti-CXCR5 (L138D7), anti-CX3CR1 (SA011F11), anti-CD370/Clec9a/DNGR1 (7H11), anti-H-2 (anti- MHCI; M1/42), anti-I-A/I-E (anti-MHCII; M5/114.15.2), anti-H2-K^b^ bound to SIINFEKL (25-D1.16), anti-NK1.1 (PK136), anti-CD49b (DX5), anti-Ly6C (HK1.4), anti-Ly6G (1A8), anti-Gr1 (RB6-8C5), anti-F4/80 (BM8), anti-TER119 (TER-119), and anti-CD209a (MMD3). Anti-CD209b (22D1) was purchased from from Invitrogen (Carlsbad, CA). Anti-Zbtb46 (U4-1374) antibody was purchased from BD Biosciences (San Jose, CA). CellTrace™ CFSE and CellTrace™ violet (CTV) Cell Proliferation Kits and LIVE/DEAD™ Fixable Yellow, Blue, and Far Red Dead Cell Stain Kits were purchased from Thermo Fisher Scientific (Seoul, Korea) and used according to the manufacturer’s instructions. FITC-conjugated ovalbumin (FITC-OVA) was purchased from Invitrogen. Fluoresbrite^®^ yellow green microsphere (YGM) beads (1.00 μm; Polysciences, Warrington, PA) were purchased and sterilized by washing according to the manufacturer’s instruction. Ovalbumin (OVA, grade V) and lipopolysaccharide (LPS) were purchased from Sigma-Aldrich (St. Louis, MO). Mouse GM-CSF and FLT3L were produced and purified in house as described previously (23, 24).

### Antibody purification and labeling

Supernatants were collected from the mAb hybridoma cultures of 2A1 and L5 (an isotype control for 2A1) (25) in DMC7 medium and filtrated before purification by affinity chromatography with protein G bead (Pierce, Rockford, IL; GE Healthcare, Piscataway, NJ). For those hybridoma cultures, DMC7 medium was prepared with ultra-low IgG FBS (Thermo Fisher Scientific) to remove bovine IgG and increase the purity of those mAbs (**Supplementary Figure 1**). Then, mAbs were concentrated using Amicon^®^ Ultra-4 Centrifugal Filter (Millipore, Burlington, MA) before quantification. Purified mAbs were fluorescently labeled using Alexa Fluor™ 488 Antibody Labeling Kit (Invitrogen) or Alexa Fluor™ 647 Antibody Labeling Kit (Invitrogen) according to the manufacturer’s instructions.

### Bone marrow culture

Mice were euthanized by asphyxiation in a CO_2_ chamber. Bone marrow cells were isolated from femurs and tibias of C57BL/6 mice at 8-14 weeks of age under sterile conditions, as described (9, 26–28), with some modifications. Briefly, femurs and tibias were detached from mice and purified from the surrounding muscle tissue by rubbing with sterile gauze followed by disinfecting in 70% ethanol for 3 minutes and washing with fresh DMEM (8). Next, the marrow was isolated from the bones by flushing using a syringe with a 23-gauge needle and disintegrated by vigorous pipetting. After lysing erythrocytes with RBC lysis buffer (BioLegend), single cell suspension of BM cells was generated by sieving through cell strainers (100 μm; SPL Life Sciences, Pocheon, Korea), counted, and cultured at 1×10^6^ cells per well for GM-BM culture or at 2×10^6^ cells per well for FL-BM culture in DMC7 medium containing GM-CSF or FLT3L with 24-well tissue culture plates for 8-10 days, as described previously (26, 27, 29). Specifically, 0.5 μg/ml of GM-CSF and 10 μg/ml of FLT3L were used for GM-BM and FL-BM cultures, respectively. During the culture, half of the medium in each well was carefully removed and replenished with fresh DMC7 medium containing GM-CSF or FLT3L every 2 days until harvest for use in subsequent experiments.

### Flow cytometry

Single cell suspension was prepared from mouse tissues or cultures and incubated with Fc receptor blocking mAb 2.4G2 for 20 minutes at 4°C followed by washing with FACS buffer (DPBS containing 2% FBS, 2mM EDTA, and 0.1% sodium azide). Then, cells were incubated with appropriate cocktails of fluorochrome-conjugated mAbs and dead cell staining dye for 30 minutes at 4°C. For intracellular staining, cells were stained for surface markers before fixation and permeabilization followed by staining intracellular molecules according to the manufacturer’s instructions (Fixation Buffer/Intracellular Staining Permeabilization Wash Buffer, BioLegend). Multiparameter analysis of each sample was performed on LSRFortessa™ flow cytometer (BD Biosciences) and flow cytometric isolation of cells was performed on BD FACSAria™ II cell sorter (BD Biosciences) at the Flow Cytometry Core Facility of the Yonsei University College of Medicine. Collected data were analyzed with FlowJo software (BD Biosciences).

### Treatment of TLR agonists

Graded doses of LPS (Sigma-Aldrich) were added into BM cultures at culture day 8. After 18 hours of incubation, cells were harvested, washed, and analyzed using flow cytometry. LPS at a final concentration of 1 μg/ml was used for *in vitro* stimulation of DCs unless otherwise noted. BM cultures at day 8 were also treated with CpG (1 μM of ODN 2216; TLRGRADE^®^, Enzo Life Science, Farmingdale, NY) or poly I:C HMW (1 μg/ml; InvivoGen) for 18 hours.

### Antigen uptake

Cultures of BM were treated with FITC-labeled OVA (100 μg/ml) or YGM beads (0.000675%) for 1 hour at 37°C or at 4°C as control. Then, each sample was washed twice with cold DMEM before being stained with appropriate reagents for flow cytometric analysis as described above.

### Generation of CHO cells expressing OVA antigens intracellularly

CHO/OT.EGFP cells, stable CHO cell-lines expressing OVA antigens, were generated as described previously (30–32). In brief, a cDNA construct encoding the open reading frame of OT.EGFP (GenBank accession number OR405547) expressing a fusion protein between enhanced green fluorescent protein (EGFP) and OVA antigen epitope peptides (i.e., OT-1 and OT-2) were cloned by PCR, sequenced, and inserted into the pCMV mammalian expression vector (Clontech, TaKaRa Bio, Shiga, Japan). Then, the stable CHO cells were generated by transfection with pCMV- OT.EGFP by Lipofectamine™ 2000 reagent (Thermo Fisher Scientific) followed by selection of G418 (1.5 mg/ml) in DMC7 medium.

### Antigen presentation and T cell proliferation

Assays for antigen presentation and T cell proliferation were performed as described previously (33). In brief, APCs were pulsed with 100 μg/ml of OVA on culture plates at 37°C for 30 minutes before being harvested by gentle pipetting and washing. Then, specific populations of APCs were isolated with flow cytometric sorter as described above. The enrichment of naive CD8^+^ OT-1 and CD4^+^ OT-2 T cells from the splenocytes of OT-1 and OT-2 mice respectively were described previously (33, 34). Naive OT-1 and OT-2 T cells were labeled with CFSE or CTV at 37°C for 10 minutes and washed with DPBS. Then, T cells (2.5×10^4^) were cultured with graded doses of APCs in 96-well round-bottom cell culture plates with DMC7 medium containing 57.2 μM β-mercaptoethanol (Sigma-Aldrich) for 3-5 days. The proliferation of T cells was assessed by the dilution of CFSE or CTV. Unpulsed APCs were co-cultured with OT-1 or OT-2 T cells in the presence of OVA (10 μg/ml) with/without LPS (1 μg/ml). For the cross-presentation assays with cell-associated antigens, 2.5×10^4^ CTV-labeled naive CD8^+^ OT-1 T cells were co-cultured with APCs plus heat-treated control CHO cells or CHO/OT.EGFP cells (1×10^5^ cells/well) for 4 days. Apoptotic CHO cells were prepared by treatment with heat at 45°C for 30 minutes. Then, the heat-treated CHO cells were plated in 24-well plates overnight before being used in the subsequent co-culture experiments. For the maturation of FL-cDC1s, media containing 20 ng/ml of GM-CSF were used for the co-culture of APCs and T cells.

### Detection of MHC class I molecules complexed with OVA epitope peptide pOT-1

At culture day 7 or 8, FL-BM culture were incubated in the presence of 100 μg/ml of endotoxin-free OVA (EndoFit Ovalbumin, InvivoGen) or 1 μg/ml of OT-1 peptide (pOT-1, SIINFEKL) for 18 hours at 37°C. For a control, 1 μg/ml of pOT-1 was added on culture for 1 hour on ice. To determine the surface levels of H2-K^b^ bound to pOT-1, cells were incubated with mAb 25-D1.16 on ice for 30 min, washed, and then analyzed by flow cytometry.

### Culture of BM precursor and progenitor cells

CDPs were isolated by gating for MHCII^-^CD3^-^CD11b^-^CD11c^-^CD19^-^CD49b^-^Sca-1^-^Ly6C^-^Ly6G^-^B220^-^IL-7Rα^-^CD115^+^CD117^+^CD135^+^ cells (11, 35, 36); pre-cDCs were isolated by gating for MHCII^-^CD3^-^CD11b^-^CD19^-^CD49b^-^CD115^-^CD117^-^Sca-1^-^Ly6C^-^Ly6G^-^CD11c^+^CD135^+^ cells (37, 38); cMoPs were isolated by gating for MHCII^-^CD3^-^CD11b^-^CD11c^-^CD19^-^CD49b^-^CD135^-^Sca-1^-^NK1.1^-^B220^-^TER119^-^Ly6G^-^CD16/32^+^CD34^+^CD115^+^CD117^+^Ly6C^+^ cells (5, 11, 39); monocytes were isolated by gating for MHCII^-^CD3^-^CD11c^-^CD19^-^CD49b^-^CD135^-^CD117^-^Sca-1^-^NK1.1^-^Ly6G^-^CD11b^+^CD115^+^Ly6C^hi^ cells (5, 11). Each population of precursor or progenitor cells were isolated from the BM of CD45.2 mice with a flow cytometric cell sorter and cultured at the densities of 5×10^3^ for CDPs, 2×10^4^ for pre-cDCs, 1×10^4^ for cMoPs, and 1×10^5^ for monocytes, respectively, per well where whole BM cells from CD45.1 mice were co-cultured as filler cells at a density of 2×10^6^ per well in DMC7 medium containing FLT3L for 6 or 8 days. LPS (1 μg/ml) was added into the culture at day 5. After 18 hours of LPS treatment, cells were harvested for flow cytometric analysis.

### Microscopic analysis

On the day 8 or 9 of BM culture, cells were harvested by gentle pipetting and stained with appropriate fluorochrome-conjugated antibodies. Each subset of the cells was isolated according to the appropriate gating strategies. Then, the isolated cells were cultured with DMC7 medium containing GM-CSF or FLT3L in 96- or 48-well flat-bottom cell culture plates overnight. Phase-contrast observations of cultures were made by means of eclipse TS100 microscope (Nikon, Tokyo, Japan) at 600× magnification. Before staining, 1×10^5^ cells were prepared on slides by cytocentrifugation (Cytospin 4, Thermo scientific) at 650 rpm for 5 minutes. For Giemsa staining, cells were visualized by Diff-Quik (Sysmex, Kobe, Japan) staining of cytospin preparations. The images were acquired with IX73 fluorescent inverted microscopes (Olympus, Tokyo, Japan). Data analysis was conducted using ImageJ.

### Transmission electron microscopy

Cells were fixed with 2% Glutaraldehyde and 2% Paraformaldehyde in 0.1M phosphate buffer (pH 7.4) for 12 hours; washed with 0.1M phosphate buffer; post-fixed with 1% OsO4 in 0.1M phosphate buffer for 2 hours; dehydrated with a series of ascending ethanol (50, 60, 70, 80, 90, 95, 100, and 100%) for 10 minutes each; infiltrated with propylene oxide for 10 minutes. Then, specimens were embedded with a Poly/Bed 812 kit (Polysciences) and polymerized in an electron microscope oven (TD-700, DOSAKA, Japan) at 65°C for 12 hours. The block was cut with a diamond knife in the ultramicrotome into 200 nm semi–thin section and stained toluidine blue for observation of optical microscope. The region of interest was then cut into 80 nm thin sections using the ultramicrotome, placed on copper grids, double stained with 3% uranyl acetate for 30 minutes and 3% lead citrate for 7 minutes, and imaged with a transmission electron microscopy (JEM-1011, JEOL, Tokyo, Japan) at the acceleration voltage of 80 kV equipped with a Megaview III CCD camera (Soft Imaging System, Germany).

### RNA sequencing and analysis

Cell subsets in the BM cultures were stained and sorted according to the suitable gating strategies as described above. Total RNA was extracted twice for each cell subset by MiniBEST universal RNA extraction kit (TaKaRa Bio) from at least 1×10^5^ cells. However, while cDC1-gated cells were fewer than 20% of the total cDC-gated cells in FL-BM culture, the number of CD83^+^ cDC1s were significantly less than 10% of the cDC1-gated cells. As a result, we were able to obtain an enough amount and high quality of RNAs only once after many trials and errors of sorting the live CD83^+^ cDC1s from FL-BM culture. Subsequent RNA sequencing (RNA-seq) procedure and analysis were performed by Macrogen (Seoul, Korea) as follows. Reverse transcription of mRNA and generation of cDNA library were carried out with SMARTer Ultra low input RNA library kit. Sequencing was performed with Illumina NovaSeq (Illumina, San Diego, CA). The raw reads from the sequencer were preprocessed to remove low quality and adapter sequence before analysis to align the processed reads to the *Mus musculus (mm10)* using HISAT v2.1.0. After alignment, StringTie v1.3.4d was used to measure the relative abundances of genes in FPKM (Fragments Per Kilobase of exon per Million mapped fragments). Multidimensional scaling method was used to visualize the similarities among samples. The larger the dissimilarity between two samples, the further apart the points representing the experiments in the picture should be. Euclidean distance was applied as the measure of dissimilarity. Hierarchical clustering analysis was also performed using complete linkage and Euclidean distance as a measure of similarity to display the expression patterns of differentially expressed transcripts which were satisfied with |fold change|≥2. Data analysis and visualization of differentially expressed genes was conducted using GraphPad Prism 8 (GraphPad Software, La Jolla, CA) R 3.5.1 (https://www.r-project.org), Multiple Experiment Viewer software (MeV), and Morpheus software (https://software.broadinstitute.org/morpheus). Venn diagram analysis was performed using the InteractiVenn website (http://www.interactivenn.net). Enrichment analyses were performed with GSEA software (Broad Institute) on the gene set curated in the Molecular Signature Database (MSigDb).

### Statistical analysis

Experiments with multiplicate samples were analyzed for statistical comparisons between different groups using with ANOVA and unpaired Student’s *t*-test using GraphPad Prism 5 (GraphPad Software, La Jolla, CA). Statistical significance is denoted by the *p* values equal or below 0.05 (*), 0.01 (**), and 0.001 (***). Data were plotted for graphs with GraphPad Prism 5 and GraphPad Prism 8.

## Results

### 2A1^+^ cells are significantly generated in both GM-BM and FL-BM cultures

**Figure 1:**
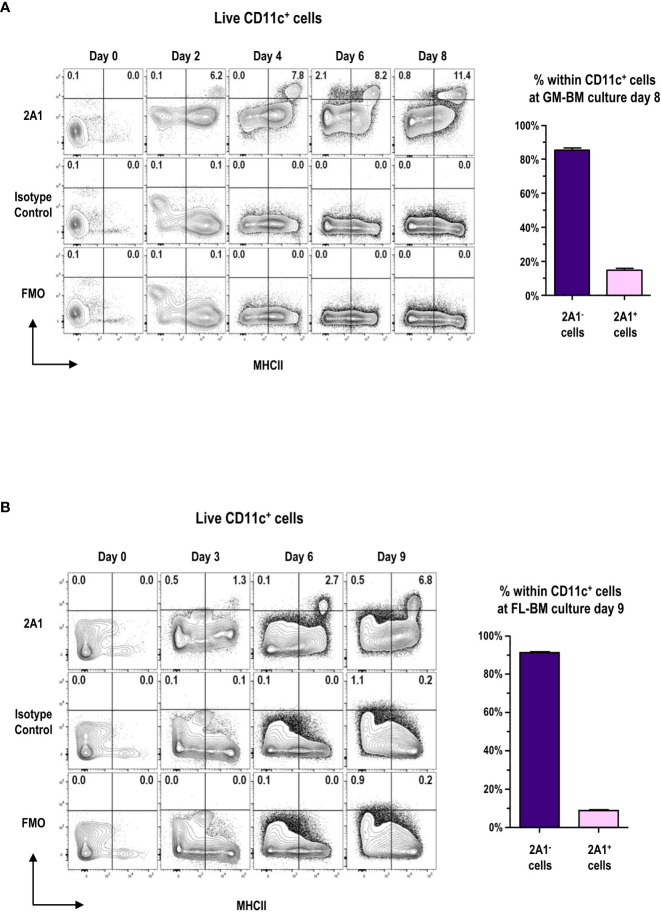
Expression of 2A1 antigen in GM-BM and FL-BM cultures. **(A)** Representative flow cytograms show kinetic change in the expression of 2A1 from CD11c^+^ cells in GM-BM culture for 8 days (left panels). Distribution of 2A1^-^ and 2A1^+^ cells within CD11c^+^ cells at day 8 of GM-BM culture (right graph). Flow cytograms are representative from 3 independent experiments. Graph is shown in mean ± SEM (n = 10). **(B)** Representative flow cytograms show kinetic change in the expression of 2A1 from CD11c^+^ cells in FL-BM culture for 9 days (left panels). Distribution of 2A1^-^ and 2A1^+^ cells within CD11c^+^ cells at day 9 of FL-BM culture (right graph). Flow cytograms are representative from 3 independent experiments. Graph is shown in mean ± SEM (n = 10).

Highly pure mAb 2A1 was prepared (**Supplementary Figure 1**) and the detection of 2A1 required the permeabilization of cells to stain with the mAb (**Supplementary Figure 2**) indicating the intracellular localization of 2A1, as reported previously (8). We examined the expression of 2A1 among the cells in GM-BM and FL-BM cultures at various time points (**Figures 1A, B**; **Supplementary Figure 3**). 2A1^+^ cells were hardly found in the BM, but became readily detectable among the MHCII^hi^ cells of both GM-BM and FL-BM cultures in 2 to 3 days after the culture. After a week of culture, around 15% of the CD11c^+^ cells in GM-BM culture and around 10% of CD11c^+^ cells in FL-BM culture became 2A1^+^.

### 2A1^+^ Cells are within the DC gate and cells within the DC gate are 2A1^+^ in GM-BM culture

**Figure 2:**
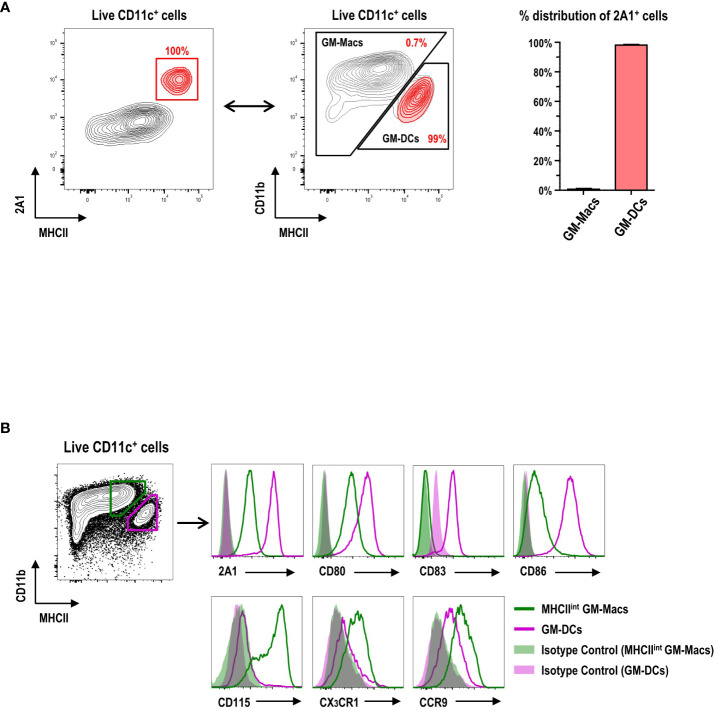
Expression of 2A1 and surface molecules in GM-DCs and GM-Macs. **(A)** Gating strategy (left panel) identifies 2A1^+^ cells (red contours) in CD11c^+^ cells at day 8 of GM-BM culture. 2A1^+^ cells (red contours in left panel) are backgated in an MHCII versus CD11b contour plot of live-gated CD11c^+^ cells (black contours in middle panel), indicating 2A1^+^ cells within the GM-DC gate. Graph (right panel) shows % distribution of 2A1^+^ cells between GM-Macs and GM-DCs. Representative flow cytograms are from 10 independent experiments. Graph is shown in mean ± SEM (n = 10). **(B)** Flow cytometric analysis of 2A1 and surface molecules in MHCII^int^ GM-Macs (green) and GM-DCs (purple) at day 8 of GM-BM culture. Filled histograms for isotype controls of the mAbs against the respective molecules. Representative flow cytograms are from at least 4 independent experiments.

CD11c^+^ cells in GM-BM culture (**Supplementary Figure 4**) are grouped into CD11b^hi^MHCII^int/lo^ GM-Macs (Macs in GM-BM culture) and CD11b^int^MHCII^hi^ GM-DCs (11). When the 2A1^+^ cells were backgated on those two populations in GM-BM culture, virtually all the 2A1^+^ cells were within the GM-DC gate (**Figure 2A**). Conversely, virtually all the cells within the GM-DC gate in GM-BM culture were 2A1^+^ (**Figure 2B**; **Supplementary Figure 5**). In addition to 2A1, GM-DCs expressed higher levels of CD80, CD83, and CD86; GM-Macs exhibited higher levels of CD115, CX_3_CR1, and CCR9 (**Figure 2B**; **Supplementary Figure 5**).

### 2A1^+^ cells are mostly present within the cDC gate in FL-BM culture

**Figure 3:**
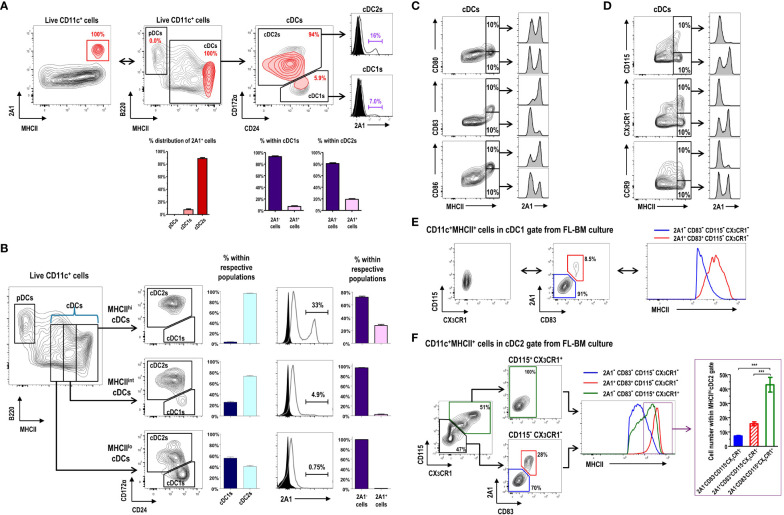
Expression of 2A1 reveals heterogeneous populations of the CD11c^+^MHCII^+^ cDC-gated cells in FL-BM culture. **(A)** Gating strategy identifying 2A1^+^ cells (red contours) in CD11c^+^ cells at day 9 of FL-BM culture (upper left panel). 2A1^+^ cell population (red contours) is backgated in an MHCII versus B220 contour plot of live-gated CD11c^+^ cells (black contours), indicating 2A1^+^ cells within the cDC1 and cDC2 gates (upper middle and right panels). Distribution of 2A1^+^ cells among DC subsets (lower left graph). Distribution of 2A1^-^ and 2A1^+^ cells within cDC1s or cDC2s (lower right graphs). Graphs are shown in mean ± SEM (n = 11) from 5 independent experiments. **(B)** Gating strategies and distributions indicating cDC1 and cDC2 populations within MHCII^lo^, MHCII^int^, or MHCII^hi^ cDC subsets at day 9 of FL-BM culture (left panels and graphs). Distributions of 2A1^-^ and 2A1^+^ cells within cDC1s or cDC2s (right panels and graphs). Graphs are shown in mean ± SEM (n = 13) from 6 independent experiments. Flow cytometric analysis of 2A1 according to the expression levels of positively **(C)** or negatively **(D)** correlating surface molecules in the MHCII^hi^ cDC gate. Flow cytometric analysis of MHCII correlating to the expression levels of 2A1, CD83, CD115, and CX_3_CR1 in the cDC1-gated **(E)** and cDC2-gated **(F)** cells from FL-BM culture. Distribution of cells among three subsets in the MHCII^hi^ cDC2 gate [purple box in right histogram in **(F)**] are shown [lower graph in purple box in **(F)**]. Representative flow cytograms are based on 3 independent experiments. Graph is shown in mean ± SEM (n = 8). ***p ≤ 0.001; one-way ANOVA test.

CD11c^+^ cells in FL-BM culture (**Supplementary Figure 6**) are grouped into B220^+^MHCII^-^ pDCs, B220^-^MHCII^+^CD24^lo/+^CD172α^-^ cDC1s, and B220^-^MHCII^+^CD24^lo/-^CD172α^+^ cDC2s (10). Virtually all the 2A1^+^ cells were MHCII^hi^ cells within the cDC gate in FL-BM culture (**Figures 3A, B**), and most of those 2A1^+^ cells were evidently within the cDC2 gate because most of the MHCII^hi^ cells in FL-BM culture were within the cDC2 gate (**Figures 3A, B**). We then characterized the surface markers of 2A1^+^ cells in comparison to 2A1^-^ cells within the cDC gates (**Figures 3C–F**; **Supplementary Figure 7**). The 2A1^+^ cells expressed higher levels of CD80, CD83, and CD86 but lower levels of CD115, CX_3_CR1, and CCR9 than the 2A1^-^ cells. Especially in MHCII^hi^ cDCs, the 2A1^+^ cells could be distinguished from the 2A1^-^ cells by the high expression of CD83 and the low expressions of CD115 and CX_3_CR1 (**Figures 3C, D**). All in all, these results indicate that the 2A1^+^ cells in FL-BM culture possess the mature DC phenotype. Indeed, all the 2A1^+^ cells within the cDC1 gate exhibited the MHCII^hi^CD83^+^CD115^-^CX_3_CR1^-^ phenotype, while all the 2A1^-^ cells within the cDC1 gate showed the MHCII^lo^CD83^-^CD115^-^CX_3_CR1^-^ phenotype (**Figure 3E**). However, the 2A1^-^ cells within the cDC2 gate from FL-BM culture displayed either CD83^-^CD115^+^CX_3_CR1^+^ or CD83^-^CD115^-^CX_3_CR1^-^ phenotypes, although all the 2A1^+^ cells within the cDC2 gate exhibited the CD83^+^CD115^-^CX_3_CR1^-^ phenotype (**Figure 3F**). Interestingly, both 2A1^-^CD83^-^CD115^+^CX_3_CR1^+^ and 2A1^+^CD83^+^CD115^-^CX_3_CR1^-^ populations in the cDC2 gate were abundantly present in MHCII^hi^ cells of the FL-BM culture.

### MHCII^hi^ cDC2-gated cells in FL-BM culture are functionally and morphologically heterogeneous

**Figure 4:**
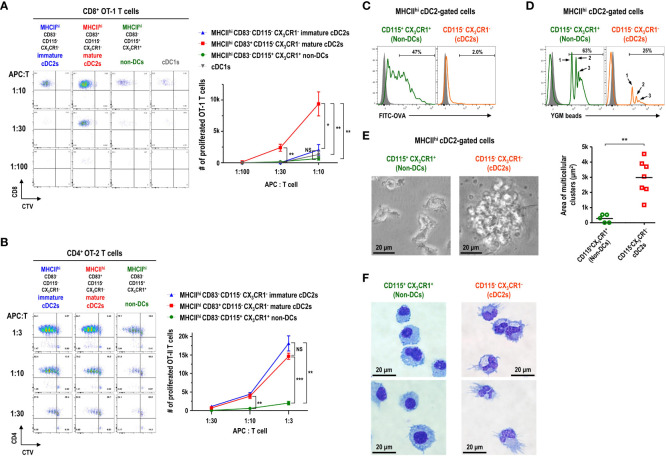
CD11c^+^MHCII^hi^ cDC2-gated cells in FL-BM culture are functionally and morphologically heterogeneous. **(A, B)** Antigen presentation to OVA-specific TCR transgenic naive T cells by CD11c^+^MHCII^+^ cDC-gated cell subpopulations. CD11c^+^MHCII^+^ cell subsets are isolated as APCs from FL-BM culture (at day 9) after being pulsed with OVA (100 μg/ml) for 30 minutes. Proliferation of CTV-labeled naive splenic CD8^+^ OT-1 **(A)** and CD4^+^ OT-2 **(B)** T cells are assessed following co-culture with graded doses of the respective APCs. Representative flow cytograms are from 5 independent experiments. Graphs are shown in mean ± SEM (n = 4). *p ≤ 0.05; **p ≤ 0.01; ***p ≤ 0.001; NS, not significant; two-way ANOVA test. In FL-BM culture (at day 9), CD11c^+^MHCII^hi^ cDC2-gated cell subpopulations are analyzed by flow cytometry after being pulsed with **(C)** soluble antigen FITC-OVA (100 μg/ml) or **(D)** particulate antigen YGM beads (0.000675%) for 1 hour at 37°C (open histograms) or at 4°C (closed histograms). Representatives flow cytograms are from 4 independent experiments. **(E)** Morphologies (left panels) of CD11c^+^MHCII^hi^ cDC2-gated cell subsets isolated from FL-BM culture (at day 9) and cultured overnight (magnification, 400×; scale bars, 20 μm). Graph (right) shows the area of cell clusters which consist of more than 2 cells. Bars indicate the mean of clusters (n = 5 for non-DCs; n = 7 for cDC2s). **p ≤ 0.01; Welch’s *t*-test. Data are collected from 3 independent experiments. **(F)** Wright-Giemsa stain of CD11c^+^MHCII^hi^ cDC2-gated cell subsets. Representatives are from 4 independent experiments.

Currently, CD11c^+^MHCII^+^ or CD11c^+^MHCII^hi^ cells in FL-BM culture are widely used as *in vitro*-generated DCs (20–22). Since most of the CD11c^+^MHCII^hi^ cells in FL-BM culture were within the cDC2 gate (**Figures 3A, B**) and those CD11c^+^MHCII^hi^ cells consisted of CD83^-^CD115^+^CX_3_CR1^+^, CD83^-^CD115^-^CX_3_CR1^-^, and CD83^+^CD115^-^CX_3_CR1^-^ subpopulations (**Figure 3F**), we set out to characterize the DC features of each subpopulation within the MHCII^hi^ cDC2 gate. As described above, the expression of 2A1 is faithfully correlated with the expression of CD83 among the cDC2-gated CD11c^+^MHCII^hi^ cell subsets in FL-BM culture. Besides, the detection of 2A1 requires the permeabilization of cells and intracellular staining which prevents and complicates the analysis of live cells. Henceforth, we utilized the cell-surface staining markers for the identification of each cell subsets in FL-BM culture. To assess the functional capacity of DCs to prime and expand T cells, naive T cells were isolated from OVA-specific TCR (i.e., CD8^+^ OT-1 and CD4^+^ OT-2) transgenic mice and used as responders to each MHCII^hi^ cDC2-gated subpopulation isolated from the OVA-pulsed FL-BM culture. In the co-cultures of graded doses of each MHCII^hi^ subpopulation with naive OVA-specific T cells, MHCII^hi^CD83^+^CD115^-^CX_3_CR1^-^ cells were far superior APCs for CD8^+^ OT-1 T cells to MHCII^hi^CD83^-^CD115^+^CX_3_CR1^+^ and MHCII^hi^CD83^-^CD115^-^CX_3_CR1^-^ cells (**Figure 4A**), while both MHCII^hi^CD83^+^CD115^-^CX_3_CR1^-^ and MHCII^hi^CD83^-^CD115^-^CX_3_CR1^-^ cells were much better APCs for CD4^+^ OT-2 T cells than MHCII^hi^CD83^-^CD115^+^CX_3_CR1^+^ cells (**Figure 4B**). Therefore, only the MHCII^hi^CD83^-^CD115^+^CX_3_CR1^+^ cDC2-gated cells exhibited the poor antigen presentation to naive T cells on both MHCI and MHCII pathways. Also, MHCII^hi^CD83^-^CD115^+^CX_3_CR1^+^ cDC2-gated cells survived most poorly in the co-culture with OVA-specific T cells (**Supplementary Figure 8**). Meanwhile, unlike cDC1s isolated from the spleen (**Supplementary Figure 9B**), cDC1s in FL-BM culture were poor at cross-presenting both soluble OVA and cell-associated OVA antigens to CD8^+^ OT-1 T cells (**Figure 4A**; **Supplementary Figure 9A**) indicating that cDC1s in FL-BM culture require an additional maturation stimulus, such as treatment with GM-CSF (**Supplementary Figure 9C**), to acquire cross-presentation capacity (40, 41).

Then, the antigen uptake ability of MHCII^hi^ subpopulations was examined from the FL-BM culture pulsed with soluble FITC-OVA or particulate 1 μm YGM beads. The MHCII^hi^CD115^+^CX_3_CR1^+^ cells were much better capable of taking up soluble antigens (**Figure 4C**) as well as phagocytosing particulate microbeads (**Figure 4D**) than the MHCII^hi^CD115^-^CX_3_CR1^-^ cells in FL-BM culture. Although the MHCII^hi^CD83^-^CD115^+^CX_3_CR1^+^ cDC2-gated cells expressed high levels of MHC class I molecules (MHCI) on surface (**Supplementary Figure 10**) and took up antigens efficiently (**Figures 4C, D**), the MHCII^hi^CD83^-^CD115^+^CX_3_CR1^+^ cDC2-gated cells were poor at processing OVA antigen and presenting OT-1 peptide epitope on MHCI (**Supplementary Figure 10**). DCs are considered as professional APCs with low endocytic and phagocytic capacities, as compared to other mononuclear phagocytes (7, 26, 42–44). Therefore, MHCII^hi^ cDC2-gated cells in the FL-BM culture likely consist of 2A1^-^CD83^-^CD115^-^CX_3_CR1^-^ immature cDC2s, 2A1^+^CD83^+^CD115^-^CX_3_CR1^-^ mature cDC2s, and 2A1^-^CD83^-^CD115^+^CX_3_CR1^+^ non-DCs.

Particular morphology is one of the key features that characterize distinct types of hematopoietic cells (24, 26, 29, 33). In the overnight culture of MHCII^hi^ cDC2-gated cells isolated from FL-BM culture, the individual morphologies of each subpopulation were examined. As expected, CD115^-^CX_3_CR1^-^ cDC2s illustrated the large clusters of non-adherent cells with noticeably long cellular processes or dendrites (**Figures 4E, F**), a typical morphology of DCs (7, 33, 45). In contrast, CD115^+^CX_3_CR1^+^ non-DCs were devoid of the morphology and clustering of DCs (**Figures 4E, F**). Besides, CD115^-^CX_3_CR1^-^ cDC2s exhibited the typical electron microscopic features of DCs (12, 46–48) that the irregularly shaped nuclei possessed a dense rim of heterochromatin and small nucleoli; the cytoplasm comprised many scattered mitochondria, short slips of rough endoplasmic reticulum, few electron-dense membrane-bound lysosomes but many electron-lucent vesicles including multivesicular bodies of varying size in the Golgi region (**Supplementary Figure 11**), while CD115^+^CX_3_CR1^+^ non-DCs displayed more frequent electron-dense membrane-bound lysosomes (**Supplementary Figure 12**).

### Gene expression profiles indicate that MHCII^hi^ cDC2-gated non-DCs in FL-BM culture are closely related to MHCII^int^ GM-Macs

**Figure 5:**
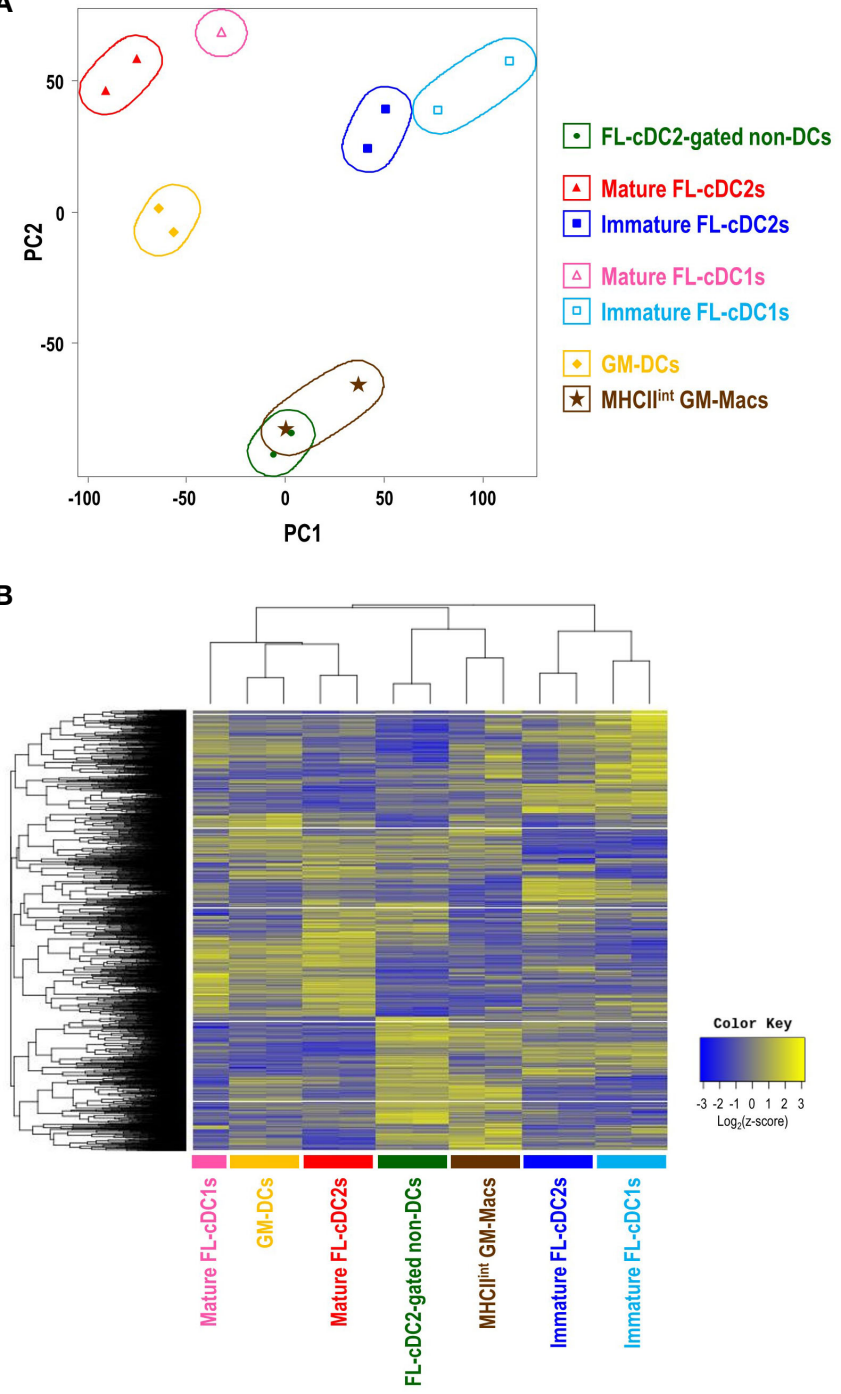
Transcriptome analysis reveals the close relationship between MHCII^int^ GM-Macs and MHCII^hi^CD115^+^CX3CR1^+^ cDC2-gated non-DCs in FL-BM culture. **(A)** Principal component analysis (PCA) of transcriptomes of cell subsets in GM-BM and FL-BM cultures. **(B)** Heatmap displays the one-way hierarchical clustering using z-score for normalized value of randomly selected genes that are differentially expressed among cell subsets in GM-BM and FL-BM cultures.

Bulk RNA sequencing (RNA-Seq) was performed to check the distinctive gene expression profile of each cell subset in GM-BM and FL-BM cultures, which are GM-DCs and MHCII^int^ GM-Macs from GM-BM culture; MHCII^hi^ FL-cDC2-gated non-DCs, mature FL-cDC2s, immature FL-cDC2s, mature FL-cDC1s, and immature FL-cDC1s from FL-BM culture. Comparative analysis of the transcriptomes revealed the transcriptional differences and similarities among those cell subsets in GM-BM and FL-BM cultures. The principal components analysis (PCA) of the transcriptomes showed that the clusters of CD83^+^ cell subsets (i.e., GM-DCs, mature FL-cDC2s, and mature FL-cDC1s) were located close to each other; so were those of CD83^-^ cDC subsets (i.e., immature FL-cDC2s and immature FL-cDC1s) and those of CD83^-^ non-DC subsets (i.e., MHCII^int^ GM-Macs and MHCII^hi^ FL-cDC2-gated non-DCs), respectively (**Figure 5A**). Also, the hierarchical cluster analysis (HCA) of the transcriptomes revealed comparable outcomes (**Figure 5B**). In FL-BM culture, CD83^+^ mature cDC2s elevated the expression of marker genes for mature DCs such as *CCR7* and *CCL22* (49–51); CD83^-^ immature cDC2s augmented the expression of DCs-related genes including *Kmo*, *Flt3*, and *CD209a* (DC-SIGN) (29, 52, 53); CD83^-^ cDC2-gated non-DCs exhibited higher levels of gene expression for macrophage signatures such as *Lyz2, Apoe, Csf1r, Clec4e, Mmp12* (54) (**Supplementary Figure 13**). Select gene expression profiles among cell subsets in FL-BM and GM-BM cultures were compared to those among MHCII^+^ mononuclear phagocytes in mice such as splenic cDC subsets, peritoneal DC subsets, and small peritoneal macrophages (SPMs) (**Supplementary Figure 14**). While each cell subset in FL-BM and GM-BM cultures was clustered close to the related cell subset in the spleen and peritoneal cavity, it was notable that FL-cDC2-gated non-DCs and MHCII^int^ GM-Macs showed close clustering with splenic cDC2s and peritoneal DC2s in the expression profiles of DC signature genes (**Supplementary Figure 14D**).

### Differentiation of MHCII^hi^ cDC2-gated non-DCs in FL-BM culture

**Figure 6:**
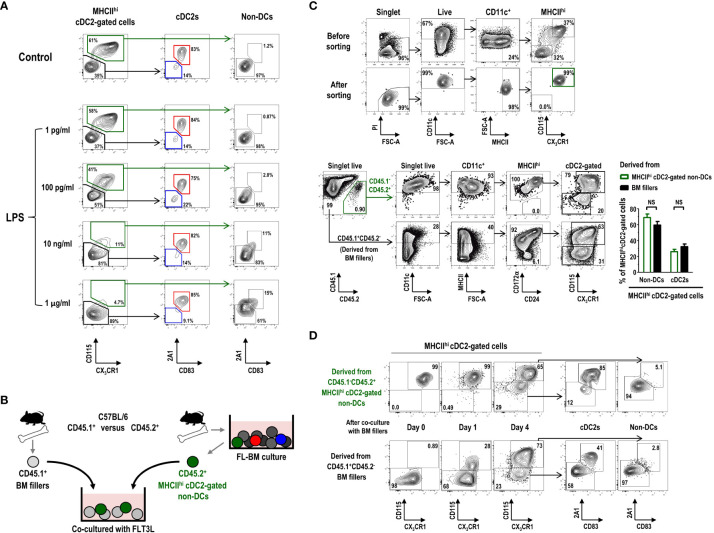
MHCII^hi^CD115^+^CX3CR1^+^ cDC2-gated non-DCs in FL-BM culture can differentiate into cDC2s. **(A)** Graded doses of LPS are added into FL-BM culture (at day 8) for 18 hours prior to the flow cytometric analysis. **(B)** Experimental scheme of co-culture shown in **(C, D)** CD45.2^+^MHCII^hi^CD115^+^CX_3_CR1^+^ cDC2-gated non-DCs are isolated from FL-BM culture (at day 7 or 8) and co-cultured with freshly isolated filler BM cells. **(C)** Gating strategies for sorting and resulting purity of CD45.2^+^MHCII^hi^ CD115^+^CX_3_CR1^+^ cDC2-gated non-DCs (upper panels). After 4 days of co-culture with BM fillers, cells are analyzed (lower panels and graph). Graph is shown in mean ± SEM (n = 3). NS, not significant; one-way ANOVA test. **(D)** Expression of 2A1 and CD83 is analyzed over the differentiation of CD45.2^+^MHCII^hi^CD115^+^CX_3_CR1^+^ cDC2-gated non-DCs as in **(B, C)** All the flow cytograms are representative from 3 independent experiments (n = 3).

CD11c^+^ cells in the gate of MHCII^int^ GM-Macs, exhibiting a similar gene expression profile as that of CD11c^+^MHCII^hi^ cDC2-gated non-DCs in FL-BM culture (**Figures 5A, B**), have been suggested to contain mixed populations of precursors or immature DCs in addition to the Macs or Mac-like cells (55). MHCII^hi^ cDC2-gated non-DCs were found to express higher levels of various toll-like receptors (TLRs) than other cells within the cDC2 gate in FL-BM culture (**Supplementary Figure 15**). Because the treatment with TLR agonists is commonly employed to induce the maturation or differentiation of DCs (7), we investigated the phenotype change of MHCII^hi^ cells in FL-BM culture following overnight treatment with graded doses of LPS. Treatment of FL-BM culture with more than 10 ng/ml of LPS sharply reduced the number of MHCII^hi^ cDC2-gated non-DCs and largely augmented the population of MHCII^hi^ mature cDC2s (**Figure 6A**; **Supplementary Figure 16**). Similarly, upon stimulation of FL-BM culture with TLR3 or TLR9 ligands, MHCII^hi^ cDC2-gated non-DCs disappeared and MHCII^hi^ mature cDC2s increased (**Supplementary Figure 17**). Meanwhile, we found that the MHCII^hi^ cDC2-gated cell subsets in FL-BM culture were not MHCII^+^RORγt^+^ APCs such as ILC3s, Janus cells, or Thetis cells (56–58) (**Supplementary Figure 18**).

Then, we tested whether the MHCII^hi^ cDC2-gated non-DCs, vanishing by stimulation of FL-BM culture with TLR ligands, could differentiate into MHCII^hi^ mature cDC2s. After isolation from the FL-BM culture at day 8, MHCII^hi^ cDC2-gated non-DCs were further co-cultured with BM filler cells from congenic mice (**Figures 6B, C**). After more than 4 days of additional co-culture with FLT3L, a significant fraction of the MHCII^hi^ cDC2-gated non-DCs became MHCII^hi^ cDC2s (**Figure 6C**). As expected, in the FL-BM co-culture, the MHCII^hi^ cDC2s which had differentiated from the MHCII^hi^ cDC2-gated non-DCs also expressed the higher levels of 2A1 and CD83 than the undifferentiated MHCII^hi^ cDC2-gated non-DCs (**Figure 6D**). Therefore, the MHCII^hi^ cDC2-gated non-DCs in FL-BM culture are precursors to the MHCII^hi^ cDC2s, i.e., MHCII^hi^ pre-cDC2s.

### MHCII^hi^ Pre-cDC2s in FL-BM culture are derived from pre-cDCs and CDPs but not from monocytes and cMoPs

**Figure 7:**
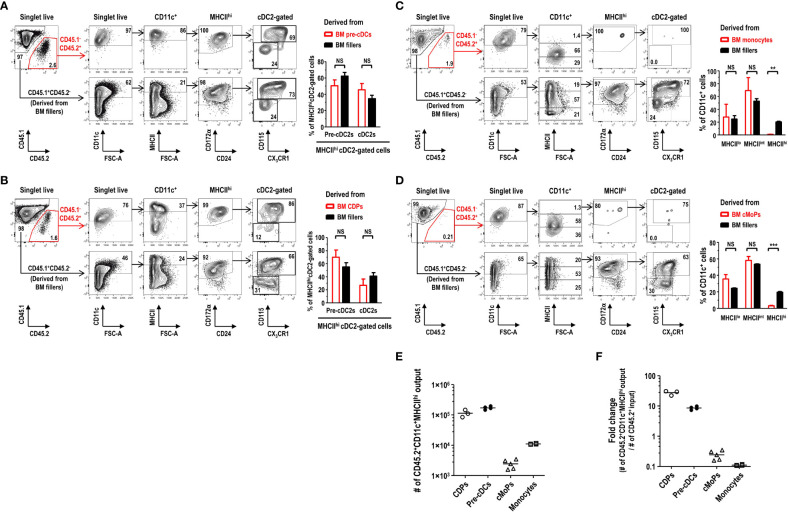
MHCII^hi^CD115^+^CX3CR1^+^ cDC2-gated pre-cDC2s in FL-BM culture are derived from pre-cDCs and CDPs but not from monocytes and cMoPs. **(A)** Gating strategies to analyze the co-culture of isolated pre-cDCs and BM fillers at day 6 (upper panels). Graph (lower) is shown in mean ± SEM (n = 4). NS, not significant; one-way ANOVA test. **(B)** Gating strategies to analyze the co-culture of isolated CDPs and BM fillers at day 8 (upper panels). Graph (lower) is shown in mean ± SEM (n = 3). NS, not significant; one-way ANOVA test. **(C)** Gating strategies to analyze the co-culture of isolated monocytes and BM fillers at day 6 (upper panels). Graph (lower) is shown in mean ± SEM (n = 4). **p ≤ 0.01; NS, not significant; one-way ANOVA test. **(D)** Gating strategies to analyze the co-culture of isolated cMoPs and BM fillers at day 8 (upper panels). Graph (lower) is shown in mean ± SEM (n = 5). ***p ≤ 0.001; NS, not significant; one-way ANOVA test. **(E, F)** Resulting output numbers and fold changes of the CD45.2^+^CD11c^+^MHCII^hi^ cells generated from the respective CD45.2^+^ BM precursor/progenitor inputs are shown. The input numbers of CD45.2^+^ precursor/progenitor are 5×10^3^ for CDPs, 2×10^4^ for pre-cDCs, 1×10^4^ for cMoPs, and 1×10^5^ for monocytes, respectively, per well. Bars indicate the mean. Data are collected from more than 3 independent experiments (n = 3 for CDPs; n = 4 for pre-cDCs; n = 5 for cMoPs; n = 4 for monocytes).

**Figure 8:**
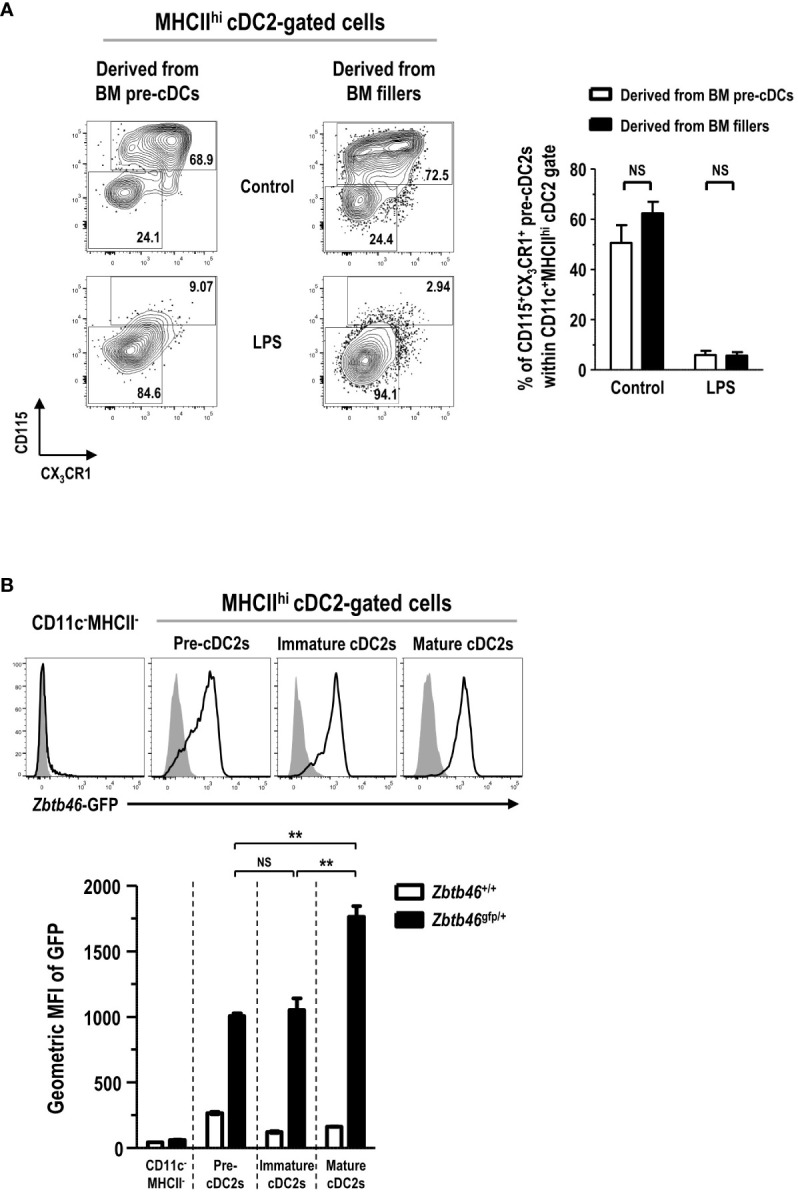
CD11c^+^MHCII^hi^ cDC2-gated CD115^+^CX_3_CR1^+^ pre-cDC2s are a close precursor to cDC2s. **(A)** LPS (1 μg/ml) is added into the co-culture of isolated pre-cDCs and BM fillers at day 5. After 18 hours, CD11c^+^MHCII^hi^ cDC2-gated cells are flow cytometrically analyzed (left panels). Representatives are from 4 independent experiments. Graph (right) is shown in mean ± SEM (n = 4). NS, not significant; one-way ANOVA test. **(B)** BMs are prepared from *Zbtb46* ^+/+^ and *Zbtb46* ^gfp/+^ mice, respectively, and cultured with FLT3L for 8 days. Representative flow cytograms (upper panels) show the expression of *Zbtb46*-GFP in different cell subsets from *Zbtb46* ^+/+^ (closed histograms) and *Zbtb46* ^gfp/+^ (open histograms) FL-BM cultures. Representatives are from 3 independent experiments. Graph (lower) shows the geometric MFI value for each cell subset in mean ± SEM (n = 3). **p ≤ 0.01; NS, not significant; one-way ANOVA test.

In the development of DC lineage cells, MDPs can differentiate either into cDCs via CDPs and pre-cDCs (35, 37) or into Mo-DCs via cMoPs and monocytes (11, 38). Hence, we examined the differentiation potential of various DC progenitors and precursors into the MHCII^hi^ pre-cDC2s in FL-BM culture. Lineage^-^CD11c^+^CD135^+^MHCII^-^ pre-cDCs, immediate precursors to cDCs (36), were isolated from the BM, and further co-cultured in FL-BM culture of congenic mice (**Supplementary Figure 19**). After 6 days of the co-culture with BM fillers and FLT3L, pre-cDCs differentiated mostly into the cells within the MHCII^hi^cDC2 gate, which consisted of MHCII^hi^ pre-cDC2s and MHCII^hi^ cDC2s in a similar ratio as found in the MHCII^hi^ cDC2-gated cells derived from the BM fillers (**Figure 7A**). Next, lineage^-^CD115^+^CD117^+^CD135^+^MHCII^-^ CDPs, progenitors to cDCs and some pDCs (37), were isolated from the BM, and co-cultured with the BM fillers of congenic mice and FLT3L (**Supplementary Figure 20**). After 8 days of the co-culture, CDPs were able to differentiate into a variety of CD11c^+^ cells in FL-BM culture where the MHCII^hi^ cDC2-gated cells comprised MHCII^hi^ pre-cDC2s and MHCII^hi^ cDC2s in a similar ratio as found in the MHCII^hi^ cDC2-gated cells derived from the BM fillers (**Figure 7B**). Monocytes, more abundant than DCs in the blood and BM, are precursors to MHCII^hi^ GM-DCs and MHCII^int/lo^ GM-Macs in GM-BM culture and can rapidly differentiate into MHCII^hi^ Mo-DCs *in vivo* during infection and inflammation (7, 11, 26). Next, we have tested whether monocytes could differentiate into the MHCII^hi^ pre-cDC2s in FL-BM culture. Lineage^-^Ly6C^hi^CD11b^+^CD115^+^MHCII^-^ monocytes were isolated from the BM, and then co-cultured with the BM fillers of congenic mice and FLT3L (**Supplementary Figure 21**). After 6 days of the co-culture, monocytes have differentiated mainly into CD11c^+^MHCII^int^ and CD11c^+^MHCII^lo^ cells but hardly into the CD11c^+^MHCII^hi^ cells in FL-BM culture (**Figure 7C**). Then, lineage^-^Ly6C^+^CD16/32^+^ CD34^+^CD115^+^CD117^+^MHCII^-^ cMoPs, progenitors to monocytes (5), were also isolated from the BM, and co-cultured with the BM fillers of congenic mice and FLT3L (**Supplementary Figure 22**). After 8 days of the co-culture, cMoPs have rarely survived and mostly differentiated into CD11c^+^MHCII^int^ and CD11c^+^MHCII^lo^ cells but not into the CD11c^+^MHCII^hi^ cells in FL-BM culture (**Figure 7D**). Therefore, in FL-BM culture, MHCII^hi^ pre-cDC2s are derived from pre-cDCs and CDPs but not from monocytes and cMoPs (**Figures 7E, F**). When we tested the co-culture of pre-cDCs and BM fillers with FLT3L to be further treated with LPS, most of the MHCII^hi^ pre-cDC2s derived from both pre-cDCs and BM fillers differentiated similarly into MHCII^hi^ cDC2s (**Figure 8A**). We also examined the expression of DC-specific transcription factor Zbtb46 and found that MHCII^hi^ pre-cDC2s expressed *Zbtb46* as highly as MHCII^hi^ immature cDC2s in FL-BM culture (**Figure 8B**), indicating that MHCII^hi^ pre-cDC2s are closely related to the DC lineage.

## Discussion

DCs have been typically identified as CD11c^+^MHCII^hi^ cells in various mouse tissues and organs (59). Likewise, from GM-BM culture as well as FL-BM culture, CD11c^+^MHCII^hi^ cells (11), if not CD11c^+^ cells (60), have been routinely isolated and used as *in vitro*-generated DCs for the numerous experiments. Our present study, however, demonstrated that only a half of the CD11c^+^MHCII^hi^ cells in FL-BM culture were DCs whereas the remaining half were precursor cells with poor antigen-presenting capacity.

Considering that Helft and others (11) had shown that GM-BM culture comprised heterogeneous populations of CD11c^+^CD11b^hi^MHCII^int^ GM-Macs and CD11c^+^CD11b^int^MHCII^hi^ GM-DCs, we began to investigate the heterogeneity of CD11c^+^ cells in GM-BM culture in terms of expression of 2A1, an intracellular antigen of mature or activated DCs (12, 14). Subsequently, in GM-BM culture, we found that CD11c^+^CD11b^hi^MHCII^int^ GM-Macs are 2A1^-^ and CD11c^+^CD11b^int^MHCII^hi^ GM-DCs are 2A1^+^, indicating that 2A1 could be a valuable marker to distinguish DCs from non-DCs among the CD11c^+^MHCII^+^ cell population.

Then, we examined the heterogeneity of CD11c^+^ cells in FL-BM culture in terms of expression of 2A1. Surprisingly, in FL-BM culture, we discovered that less than a half of the CD11c^+^MHCII^hi^ cells were 2A1^+^, but the remaining cells were 2A1^-^. Flow cytometric analysis revealed that almost of all the CD11c^+^MHCII^hi^ cells in FL-BM culture were within the cDC2 gate, and categorized into three groups: two large majorities of 2A1^+^CD83^+^CD115^-^CX_3_CR1^-^ and 2A1^-^CD83^-^CD115^+^CX_3_CR1^+^ cells and a smaller number of 2A1^-^CD83^-^CD115^-^CX_3_CR1^-^ cells. According to the assays on antigen uptake and presentation capacities, morphologies, and gene expression profiles of the CD11c^+^MHCII^hi^ cDC2-gated cell subsets, 2A1^-^CD83^-^CD115^-^CX_3_CR1^-^ cells were immature cDC2s; 2A1^+^CD83^+^CD115^-^CX_3_CR1^-^ cells were mature cDC2s; and 2A1^-^CD83^-^CD115^+^CX_3_CR1^+^ cells were non-DCs. The treatment with LPS and other TLR ligands greatly decreased both non-DCs and immature cDC2s but highly increased mature FL-cDC2s among the CD11c^+^MHCII^hi^ cDC2-gated cells in FL-BM culture. Then, the FL-BM culture with adoptively transferred cells confirmed that the MHCII^hi^ cDC2-gated non-DCs were precursors to cDC2s, i.e., MHCII^hi^ pre-cDC2s. Besides, MHCII^hi^ pre-cDC2s also showed the higher expression of DC-specific transcription factor Zbtb46 as similarly as immature cDC2s in FL-BM culture. In the meantime, the MHCII^hi^ pre-cDC2s were found generated from pre-cDCs and CDPs but not from monocytes and cMoPs, corroborating that the MHCII^hi^ pre-cDC2s have close lineage to cDCs but not Mo-DCs. All in all, our present study identified and characterized a novel cDC precursor population, exhibiting a CD11c^+^MHCII^hi^CD115^+^CX_3_CR1^+^ phenotype, in FL-BM culture.

DCs are the most efficient professional PCs and typically form clusters under the *in vitro* culture condition. Meanwhile, DCs are much poorer at taking up antigens than other APCs such as monocytes and macrophages (7, 26, 43, 44). For example, Mo-DCs are much poorer at taking up antigens than monocytes, i.e., their precursors. Therefore, the loss of the strong ability to take up antigens and other characteristics of monocytes and macrophages is considered as key features of fully differentiated Mo-DCs (7). In this study, we discovered that MHCII^hi^ cDC2-gated CD115^+^CX3CR1^+^ cells are precursors to cDC2s in FL-BM culture. The newly identified MHCII^hi^ cDC2 precursor cells might possess certain characteristics similar to those of monocytes and macrophages prior to differentiating into cDC2s in FL-BM culture. Besides, there are still unresolved differences in view on the nature of MHCII^int^ GM-Macs (11, 55, 61, 62). Helft and others classified CD11c^+^CD11b^hi^MHCII^int^ cells in GM-BM culture as Macs or GM-Macs on the basis of their poor antigen presentation capacity, morphology, progenitor/precursor, and gene expression profile (11), whereas Lutz and others argued that GM-Macs might be a mixture of macrophages and immature DCs (55). The features of MHCII^hi^ pre-cDC2s that we have identified and characterized in the present study are quite similar to those of GM-Macs in many aspects: lack of dendritic morphology and cell clustering, poor antigen presentation, CD11c^+^2A1^-^CD83^-^CD115^+^CX3CR1^+^ phenotype, and similar gene expression profiles. Those two cell populations also have some notable differences: MHCII^hi^ pre-cDC2s in FL-BM culture have a MHCII^hi^ phenotype and are derived efficiently from pre-cDCs and CDPs but not from monocytes and cMoPs; GM-Macs have a MHCII^int^ phenotype and are derived efficiently from monocytes and cMoPs. Therefore, it will be intriguing to investigate and compare the differentiation potentials of those two cell populations under a variety of conditions.

It is well known that FLT3L-induced BM-DCs do not fully recapitulate the phenotype of cDCs *in vivo*. It is also recognized that developing better phenotypes of FLT3L-induced BM-DCs *in vitro* requires the addition of still many unidentified factors into FL-BM culture (63). Our discovery of a large number of MHCII^hi^ pre-cDC2s in FL-BM culture may reflect less than perfect *in-vitro* culture conditions for FLT3L-induced BM-DCs. If so, it is not likely that the *in-vivo* counterpart cells of the MHCII^hi^ pre-cDC2s exist in large numbers. Nonetheless, in the future studies, it will be important to determine and characterize an *in-vivo* counterpart of the MHCII^hi^ pre-cDC2s identified in FL-BM culture.

## Data availability statement

The datasets presented in this study can be found in online repositories. The names of the repository/repositories and accession number(s) can be found below: https://www.ncbi.nlm.nih.gov/geo/query/acc.cgi?acc=GSE225321, GSE225321.

## Ethics statement

The animal study was approved by The Institutional Animal Care and Use Committee of the Yonsei University College of Medicine. The study was conducted in accordance with the local legislation and institutional requirements.

## Author contributions

Conceptualization: CP. Methodology: HI, JSP, HS, SR, MS, WC, SP, SH, HN, and CP. Formal Analysis: HI, HN, and CP. Investigation: HI, JSP, SR, WC, SH, and HN. Resources: JP, LC, and T-GK. Data Curation: HI. Writing – Original Draft: HI and CP. Writing – Review & Editing: HI, MC, HN, and CP. Visualization: HI and JSP. Supervision: HN and CP. Project Administration: HI and HN. Funding Acquisition: HN, MC, and CP. All authors contributed to the article and approved the submitted version.

## Glossary

**Table:**

APC	antigen presenting cell
BM	bone marrow
BM-DC	BM-derived DC
cDC	classical DC
CDP	common DC progenitor
CLP	common lymphoid progenitor
cMoP	common monocyte progenitor
CTV	CellTrace™ Violet
DC	dendritic cell
EGFP	enhanced green fluorescent protein
FBS	fetal bovine serum
FITC-OVA	FITC-conjugated OVA
FL-BM culture	BM culture with FLT3L
FL-cDC	cDC in FL-BM culture
FLT3L	FMS-like tyrosine kinase 3 ligand
FPKM	fragments per kilobase of exon per million mapped fragments
GM-BM culture	BM culture with GM-CSF
GM-CSF	granulocyte macrophage-colony stimulating factor
GM-DC	DC in GM-BM culture
GM-Mac	Mac in GM-BM culture
HCA	hierarchical cluster analysis
LPS	lipopolysaccharide
mAb	monoclonal antibody
Mac	macrophage
MDP	monocyte and DC progenitor
MFI	mean fluorescence intensity
MHC	major histocompatibility complex
MHCI	MHC class I molecule
MHCII	MHC class II molecule
Mo-DC	monocyte-derived DC
OVA	ovalbumin
PCA	principal components analysis
pDCs	plasmacytoid DCs
RNA-seq	RNA sequencing
SPMs	small peritoneal macrophages
TCR	T cell receptor
Tg	transgenic
TLR	Toll-like receptor
YGM	yellow green microsphere
