# Supplementary material for: Identification of dendritic cell precursor from the CD11c+ cells expressing high levels of MHC class II molecules in the culture of bone marrow with FLT3 ligand
Source: Front Immunol. 2023 Nov 29;14:1179981. doi: 10.3389/fimmu.2023.1179981 (PMC10716454; doi:10.3389/fimmu.2023.1179981)
Supplement: Supplementary file 1 [file Presentation_1.pptx]

## Slide 1
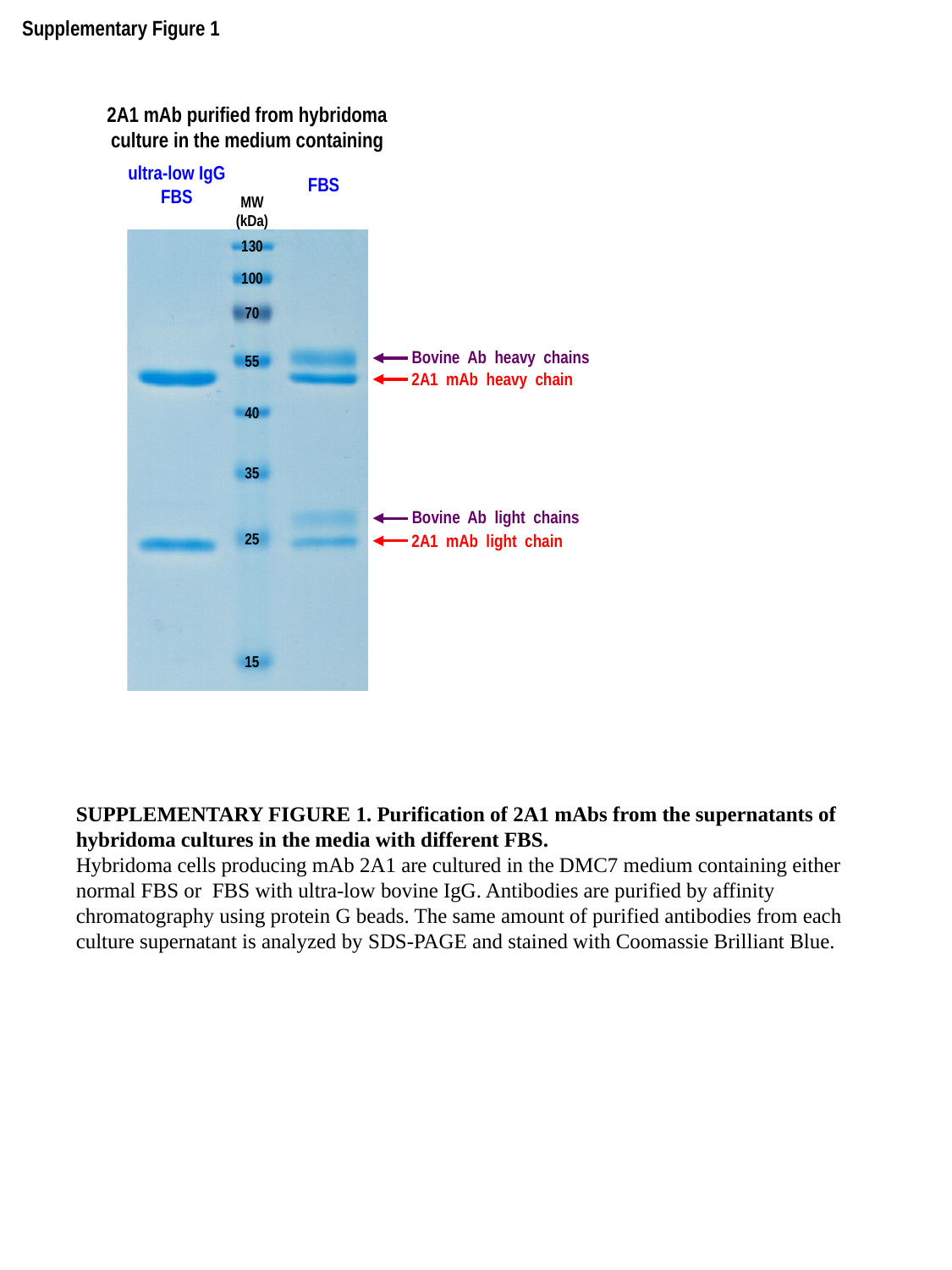

Supplementary Figure 1
2A1 mAb purified from hybridoma
culture in the medium containing
ultra-low IgG
FBS
FBS
MW
(kDa)
130
100
70
55
40
35
25
15
Bovine Ab heavy chains
2A1 mAb heavy chain
Bovine Ab light chains
2A1 mAb light chain
SUPPLEMENTARY FIGURE 1. Purification of 2A1 mAbs from the supernatants of hybridoma cultures in the media with different FBS.
Hybridoma cells producing mAb 2A1 are cultured in the DMC7 medium containing either normal FBS or FBS with ultra-low bovine IgG. Antibodies are purified by affinity chromatography using protein G beads. The same amount of purified antibodies from each culture supernatant is analyzed by SDS-PAGE and stained with Coomassie Brilliant Blue.

## Slide 2
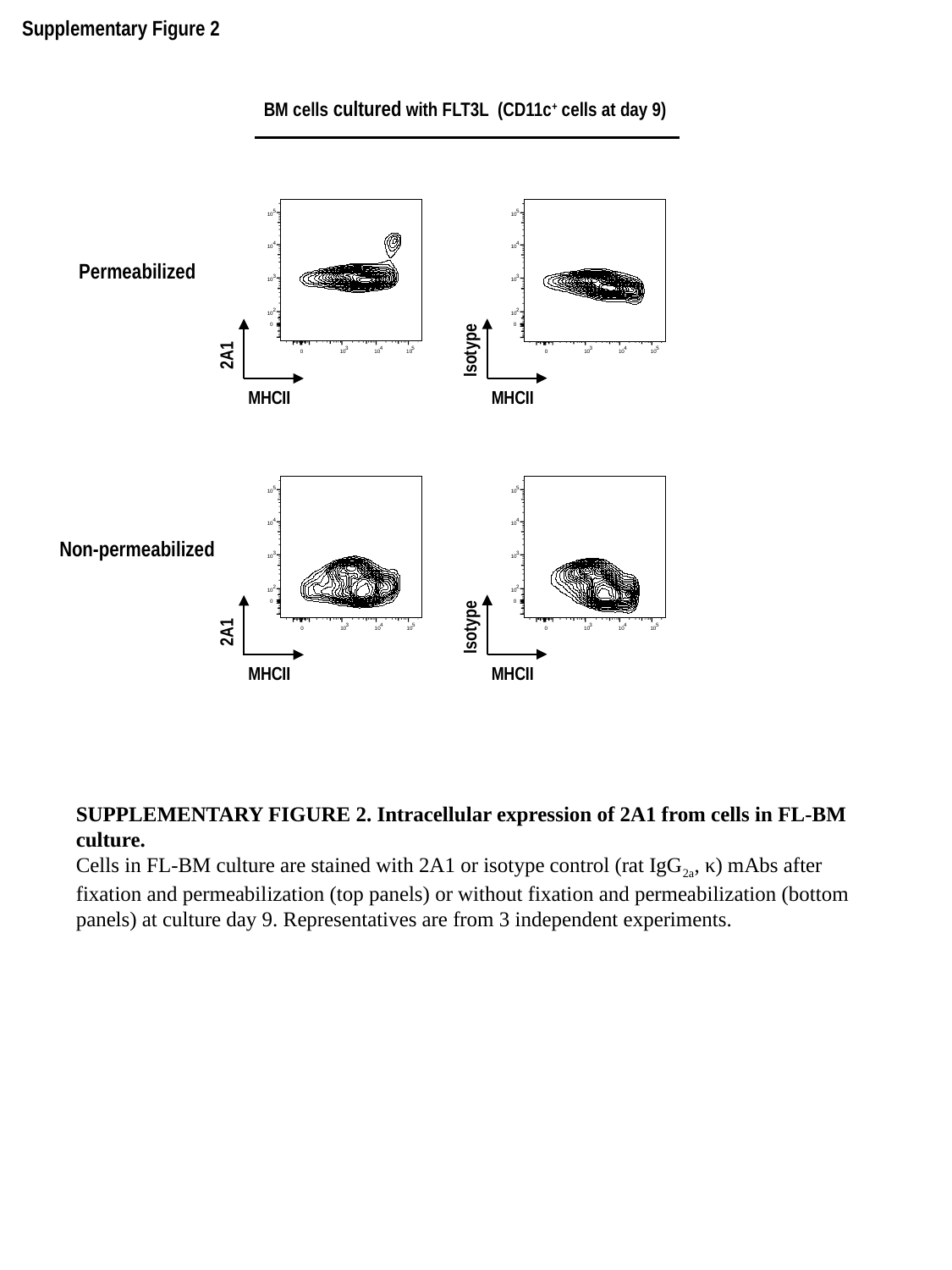

Supplementary Figure 2
BM cells cultured with FLT3L (CD11c+ cells at day 9)
5
10
4
10
3
10
2
10
0
3
4
5
0
10
10
10
2A1
MHCII
5
10
4
10
3
10
2
10
0
3
4
5
0
10
10
10
Isotype
MHCII
Permeabilized
5
10
4
10
3
10
2
10
0
3
4
5
0
10
10
10
2A1
MHCII
5
10
4
10
3
10
2
10
0
3
4
5
0
10
10
10
Isotype
MHCII
Non-permeabilized
SUPPLEMENTARY FIGURE 2. Intracellular expression of 2A1 from cells in FL-BM culture.
Cells in FL-BM culture are stained with 2A1 or isotype control (rat IgG2a, κ) mAbs after fixation and permeabilization (top panels) or without fixation and permeabilization (bottom panels) at culture day 9. Representatives are from 3 independent experiments.

## Slide 3
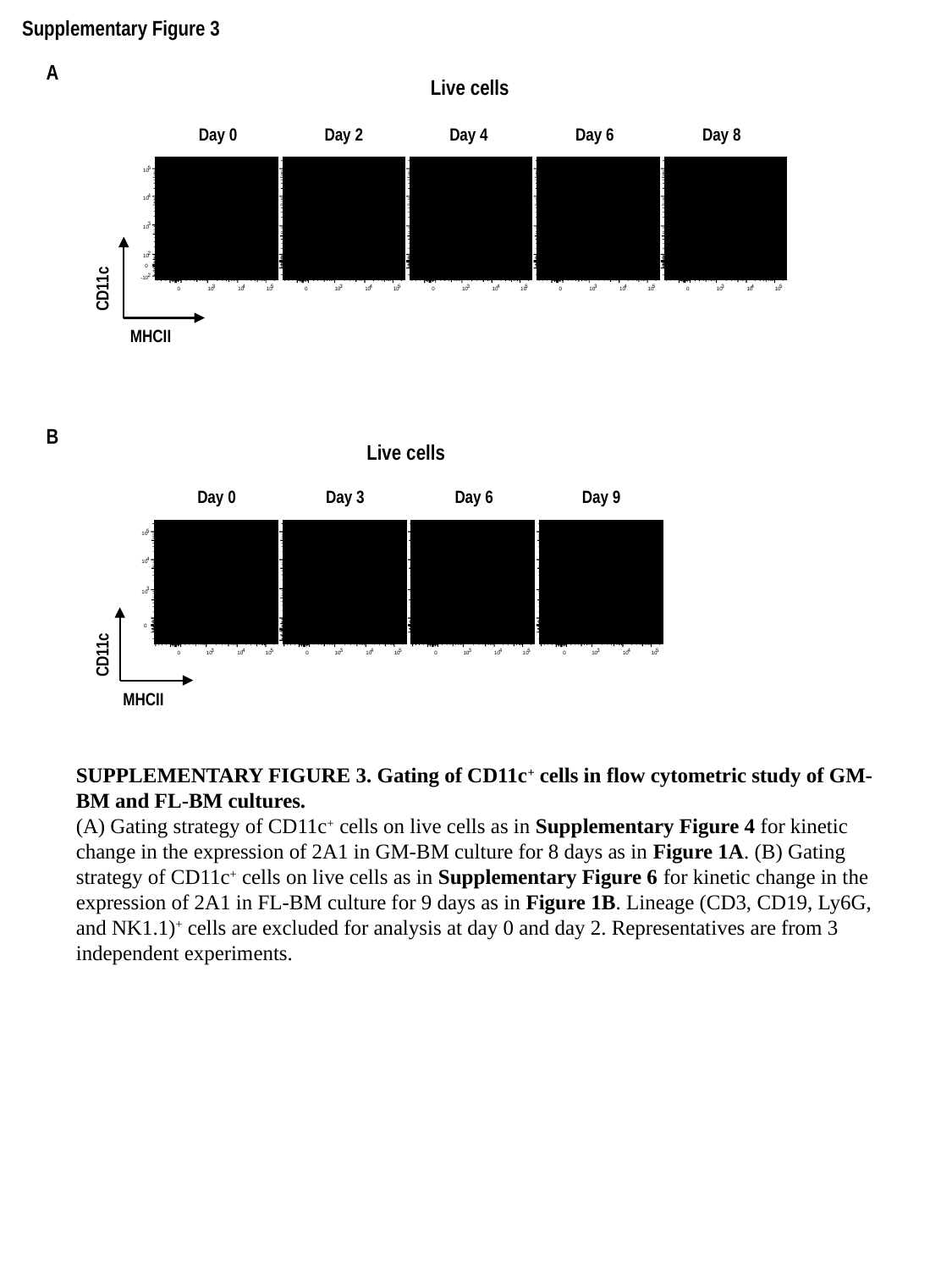

Supplementary Figure 3
A
Live cells
Day 0
Day 2
Day 4
Day 6
Day 8
2.5
5
10
4
10
3
10
2
10
0
2
-10
3
4
5
0
10
10
10
12
3
4
5
0
10
10
10
42
3
4
5
0
10
10
10
54
3
4
5
0
10
10
10
50
3
4
5
0
10
10
10
CD11c
MHCII
B
Live cells
Day 0
Day 3
Day 6
Day 9
1.7
5
10
4
10
3
10
0
3
4
5
0
10
10
10
12
3
4
5
0
10
10
10
56
3
4
5
0
10
10
10
68
3
4
5
0
10
10
10
CD11c
MHCII
SUPPLEMENTARY FIGURE 3. Gating of CD11c+ cells in flow cytometric study of GM-BM and FL-BM cultures.
(A) Gating strategy of CD11c+ cells on live cells as in Supplementary Figure 4 for kinetic change in the expression of 2A1 in GM-BM culture for 8 days as in Figure 1A. (B) Gating strategy of CD11c+ cells on live cells as in Supplementary Figure 6 for kinetic change in the expression of 2A1 in FL-BM culture for 9 days as in Figure 1B. Lineage (CD3, CD19, Ly6G, and NK1.1)+ cells are excluded for analysis at day 0 and day 2. Representatives are from 3 independent experiments.

## Slide 4
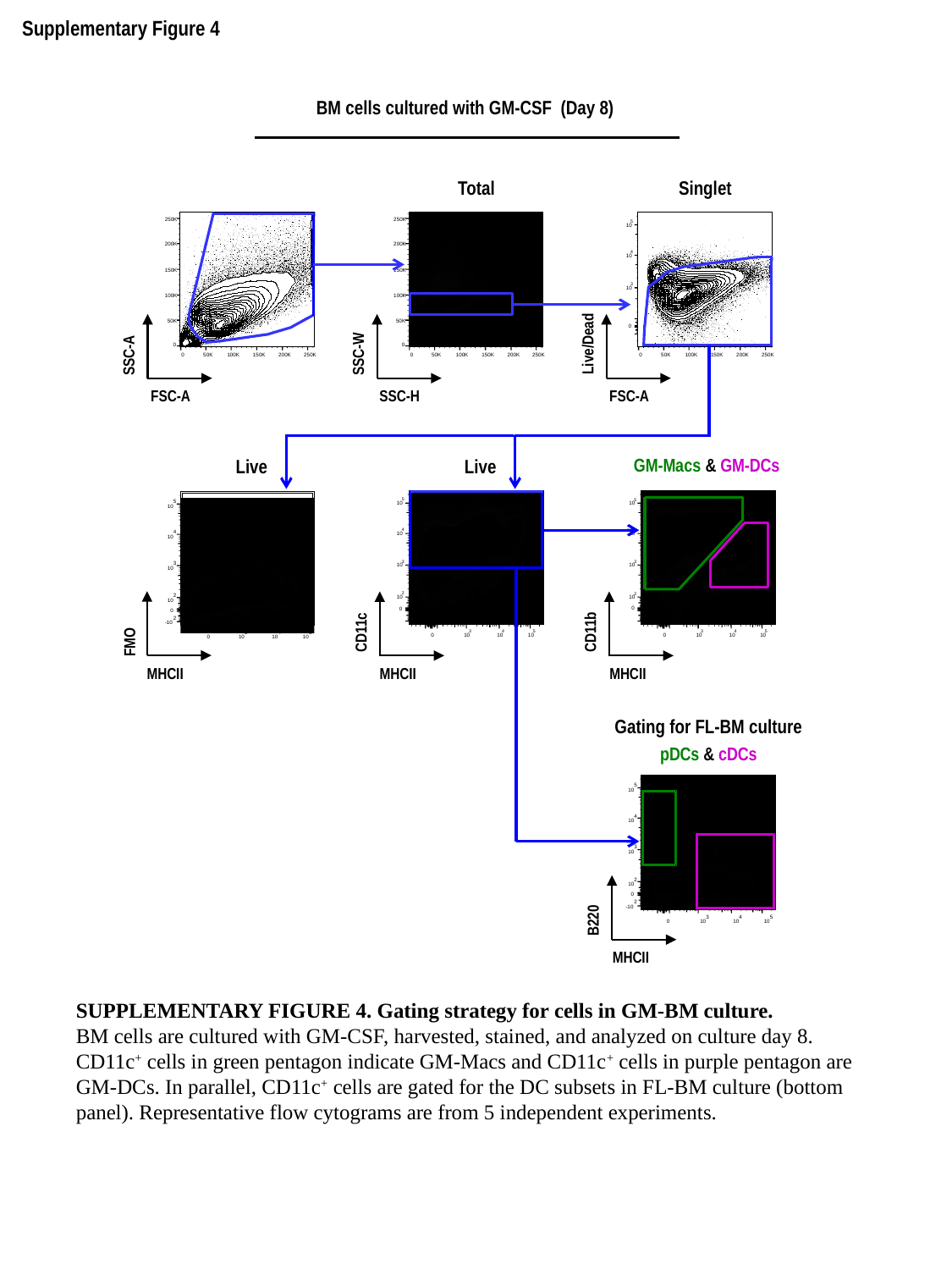

Supplementary Figure 4
BM cells cultured with GM-CSF (Day 8)
Total
Singlet
250K
200K
150K
100K
50K
0
0
50K
100K
150K
200K
250K
250K
200K
150K
100K
50K
0
0
50K
100K
150K
200K
250K
5
10
4
10
3
10
0
0
50K
100K
150K
200K
250K
Live/Dead
FSC-A
SSC-A
FSC-A
SSC-W
SSC-H
Live
GM-Macs & GM-DCs
Live
5
10
4
10
3
10
2
10
0
3
4
5
0
10
10
10
5
10
4
10
3
10
2
10
0
3
4
5
0
10
10
10
5
10
4
10
3
10
2
10
0
2
-10
3
4
5
0
10
10
10
FMO
MHCII
CD11c
MHCII
CD11b
MHCII
Gating for FL-BM culture
pDCs & cDCs
5
10
4
10
3
10
2
10
0
2
-10
3
4
5
0
10
10
10
B220
MHCII
SUPPLEMENTARY FIGURE 4. Gating strategy for cells in GM-BM culture.
BM cells are cultured with GM-CSF, harvested, stained, and analyzed on culture day 8. CD11c+ cells in green pentagon indicate GM-Macs and CD11c+ cells in purple pentagon are GM-DCs. In parallel, CD11c+ cells are gated for the DC subsets in FL-BM culture (bottom panel). Representative flow cytograms are from 5 independent experiments.

## Slide 5
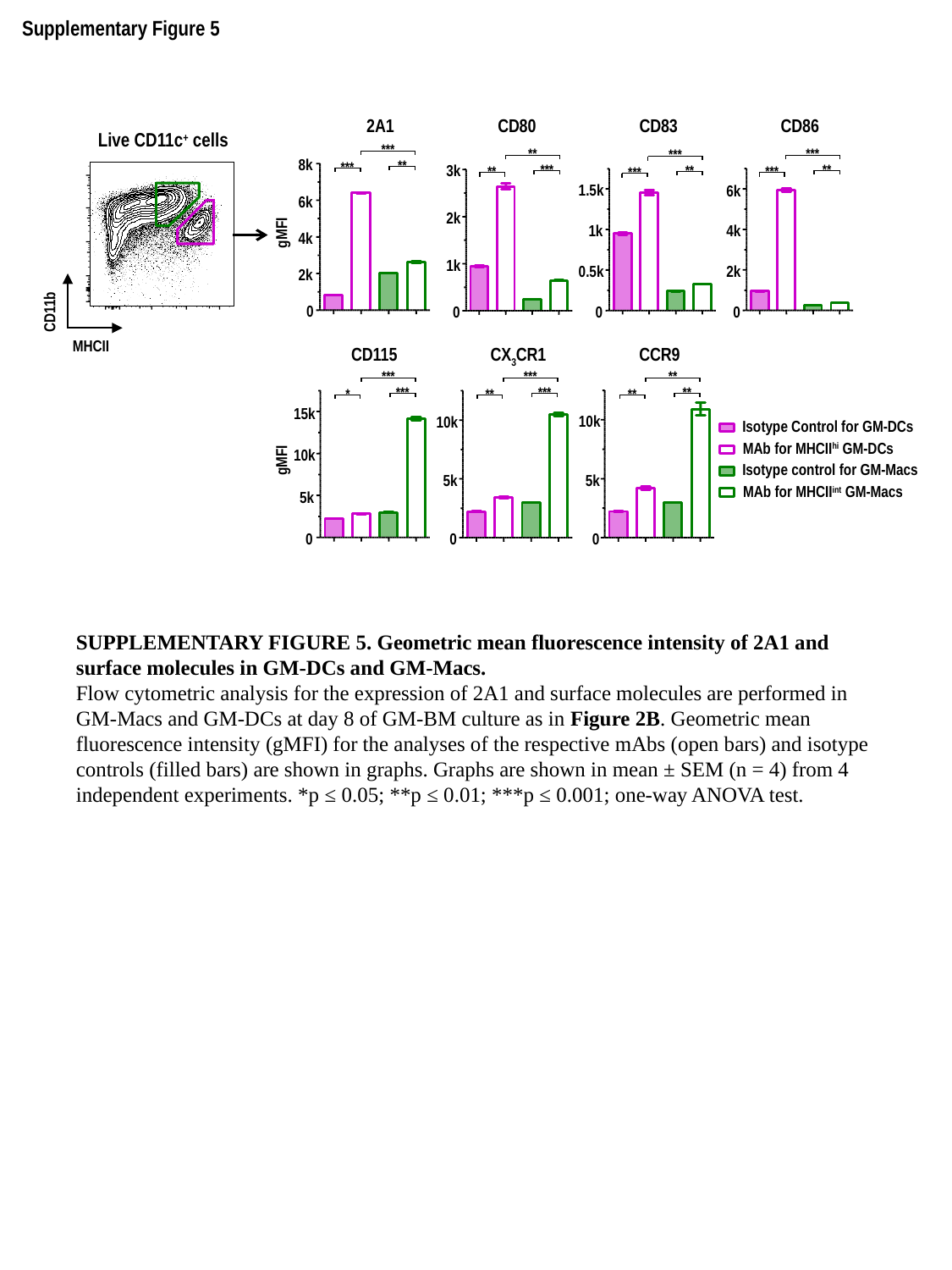

Supplementary Figure 5
2A1
CD80
CD83
CD86
Live CD11c+ cells
CD11b
MHCII
***
8k
6k
4k
2k
0
**
***
**
***
3k
2k
1k
0
**
***
**
***
6k
4k
2k
0
***
**
***
1.5k
1k
0.5k
0
gMFI
CCR9
CD115
CX3CR1
***
***
**
10k
5k
0
**
**
**
10k
5k
0
***
***
*
15k
10k
5k
0
Isotype Control for GM-DCs
MAb for MHCIIhi GM-DCs
Isotype control for GM-Macs
MAb for MHCIIint GM-Macs
gMFI
SUPPLEMENTARY FIGURE 5. Geometric mean fluorescence intensity of 2A1 and surface molecules in GM-DCs and GM-Macs.
Flow cytometric analysis for the expression of 2A1 and surface molecules are performed in GM-Macs and GM-DCs at day 8 of GM-BM culture as in Figure 2B. Geometric mean fluorescence intensity (gMFI) for the analyses of the respective mAbs (open bars) and isotype controls (filled bars) are shown in graphs. Graphs are shown in mean ± SEM (n = 4) from 4 independent experiments. *p ≤ 0.05; **p ≤ 0.01; ***p ≤ 0.001; one-way ANOVA test.

## Slide 6
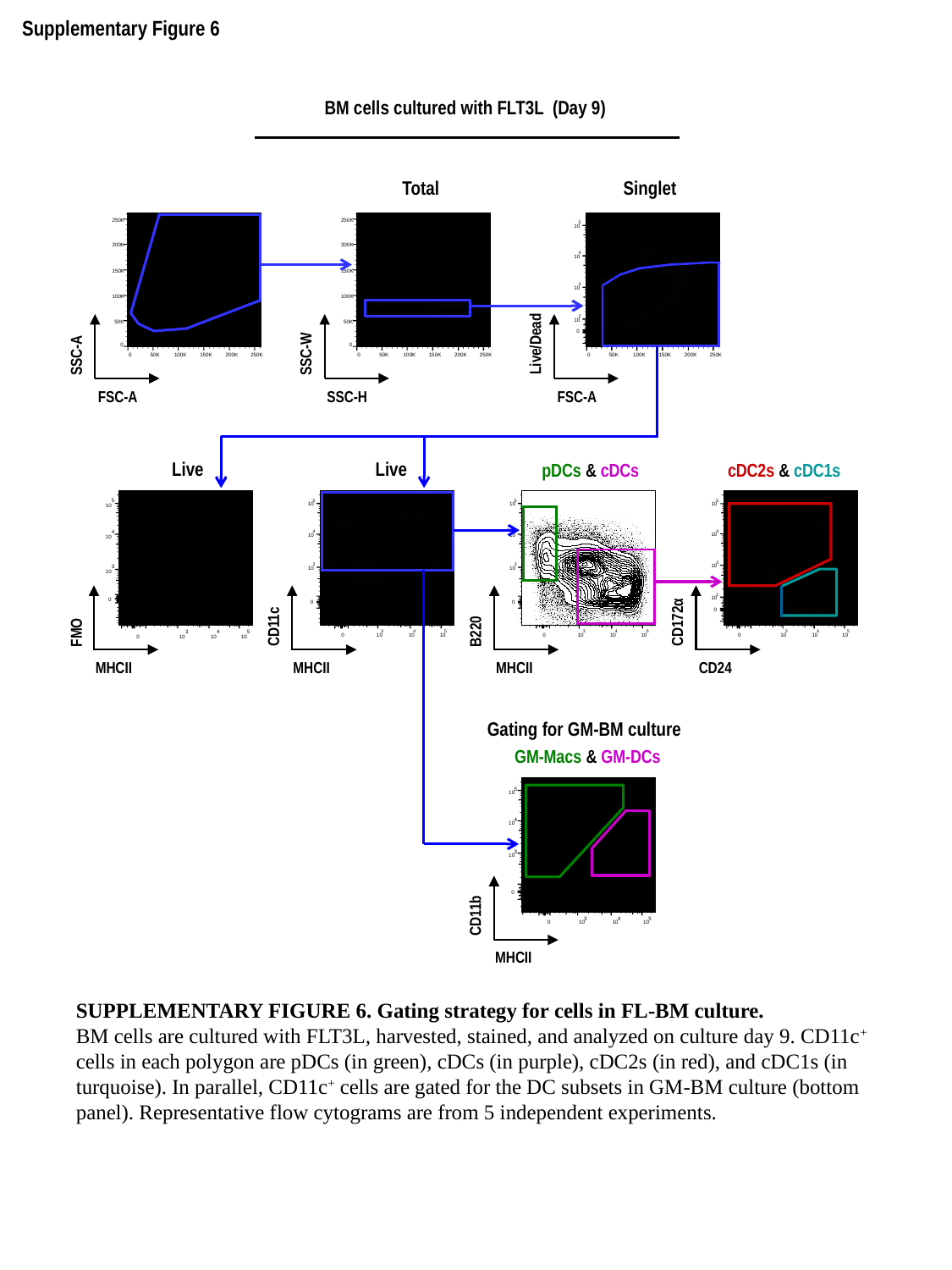

Supplementary Figure 6
BM cells cultured with FLT3L (Day 9)
Total
Singlet
250K
200K
150K
100K
50K
0
0
50K
100K
150K
200K
250K
250K
200K
150K
100K
50K
0
0
50K
100K
150K
200K
250K
5
10
4
10
3
10
2
10
0
0
50K
100K
150K
200K
250K
Live/Dead
FSC-A
SSC-A
FSC-A
SSC-W
SSC-H
Live
Live
pDCs & cDCs
cDC2s & cDC1s
5
10
4
10
3
10
0
3
4
5
0
10
10
10
5
10
4
10
3
10
0
3
4
5
0
10
10
10
5
10
4
10
3
10
0
3
4
5
0
10
10
10
5
10
4
10
3
10
2
10
0
3
4
5
0
10
10
10
CD172α
CD24
CD11c
MHCII
FMO
MHCII
B220
MHCII
Gating for GM-BM culture
GM-Macs & GM-DCs
5
10
4
10
3
10
0
3
4
5
0
10
10
10
CD11b
MHCII
SUPPLEMENTARY FIGURE 6. Gating strategy for cells in FL-BM culture.
BM cells are cultured with FLT3L, harvested, stained, and analyzed on culture day 9. CD11c+ cells in each polygon are pDCs (in green), cDCs (in purple), cDC2s (in red), and cDC1s (in turquoise). In parallel, CD11c+ cells are gated for the DC subsets in GM-BM culture (bottom panel). Representative flow cytograms are from 5 independent experiments.

## Slide 7
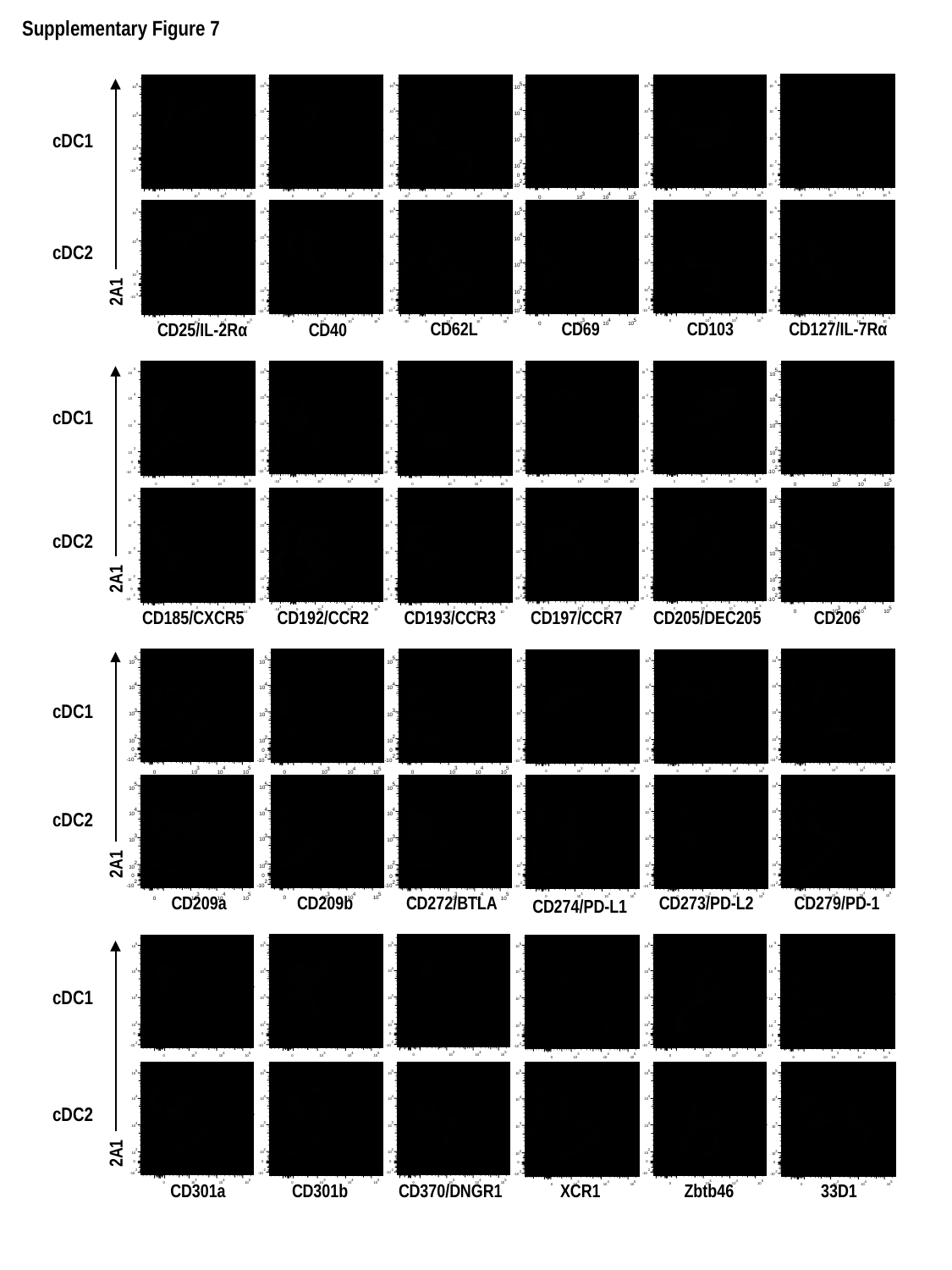

Supplementary Figure 7
0.87
0.097
5
10
4
10
3
10
2
10
98.7
0.29
0
2
-10
3
4
5
0
10
10
10
9.67
0.65
5
10
4
10
3
10
2
10
86.9
2.77
0
2
-10
3
4
5
0
10
10
10
CD127/IL-7Rα
2.39
0.077
5
10
4
10
3
10
2
10
96.8
0.73
0
2
-10
3
4
5
0
10
10
10
5.88
0.077
5
10
4
10
3
10
2
10
92.3
1.71
0
2
-10
3
4
5
0
10
10
10
CD69
4.6
7.8
5
10
4
10
3
10
2
10
0
36.6
51.3
2
-10
3
4
5
0
10
10
10
12.6
0.8
5
10
4
10
3
10
2
10
0
79.6
7.1
2
-10
3
4
5
0
10
10
10
CD103
8.55
12.9
5
10
4
10
3
10
0
3
-10
77.7
0.86
3
4
5
0
10
10
10
9.13
3.98
5
10
4
10
3
10
0
3
-10
86.7
0.16
3
4
5
0
10
10
10
CD25/IL-2Rα
7.6
0.2
5
10
4
10
3
10
2
10
75.1
17.1
0
2
-10
3
3
4
5
-10
0
10
10
10
12.8
0.4
5
10
4
10
3
10
2
10
76.6
10.3
0
2
-10
3
3
4
5
-10
0
10
10
10
CD62L
4.73
4.25
5
10
4
10
3
10
2
10
90.4
0.65
0
2
-10
3
4
5
0
10
10
10
10.2
2.78
5
10
4
10
3
10
2
10
83.6
3.46
0
2
-10
3
4
5
0
10
10
10
CD40
2A1
cDC1
cDC2
7.7
0.1
5
10
4
10
3
10
2
10
90.8
1.4
0
2
-10
3
3
4
5
-10
0
10
10
10
13.0
0.3
5
10
4
10
3
10
2
10
85.2
1.5
0
2
-10
3
3
4
5
-10
0
10
10
10
CD192/CCR2
2.62
0.063
5
10
4
10
3
10
2
10
94.1
3.21
0
2
-10
3
4
5
0
10
10
10
6.74
0.11
5
10
4
10
3
10
2
10
91.9
1.25
0
2
-10
3
4
5
0
10
10
10
CD193/CCR3
2.71
0.031
5
10
4
10
3
10
2
10
96.7
0.56
0
2
-10
3
4
5
0
10
10
10
6.82
0.13
5
10
4
10
3
10
2
10
92.1
0.98
0
2
-10
3
4
5
0
10
10
10
CD185/CXCR5
4.3
1.8
5
10
4
10
3
10
2
10
92.9
1.0
0
2
-10
3
4
5
0
10
10
10
9.4
0.1
5
10
4
10
3
10
2
10
89.9
0.6
0
2
-10
3
4
5
0
10
10
10
CD197/CCR7
0.33
5.66
5
10
4
10
3
10
2
10
84.1
9.93
0
2
-10
3
4
5
0
10
10
10
5.29
14.0
5
10
4
10
3
10
2
10
74.3
6.38
0
2
-10
3
4
5
0
10
10
10
CD205/DEC205
2.61
0.041
5
10
4
10
3
10
2
10
97.3
0.014
0
2
-10
3
4
5
0
10
10
10
6.34
0.14
5
10
4
10
3
10
2
10
89.2
4.29
0
2
-10
3
4
5
0
10
10
10
CD206
2A1
cDC1
cDC2
1.24
1.85
5
10
4
10
3
10
2
10
61.3
35.6
0
2
-10
3
4
5
0
10
10
10
1.77
4.09
5
10
4
10
3
10
2
10
20.8
73.3
0
2
-10
3
4
5
0
10
10
10
CD209a
9.8
0.5
5
10
4
10
3
10
2
10
83.3
6.4
0
2
-10
3
4
5
0
10
10
10
15.4
0.2
5
10
4
10
3
10
2
10
82.6
1.8
0
2
-10
3
4
5
0
10
10
10
CD279/PD-1
1.13
0.54
5
10
4
10
3
10
2
10
10.3
88.0
0
2
-10
3
4
5
0
10
10
10
3.69
2.39
5
10
4
10
3
10
2
10
29.3
64.6
0
2
-10
3
4
5
0
10
10
10
CD272/BTLA
3.25
0.015
5
10
4
10
3
10
2
10
96.3
0.41
0
2
-10
3
4
5
0
10
10
10
5.97
0.11
5
10
4
10
3
10
2
10
85.8
8.13
0
2
-10
3
4
5
0
10
10
10
CD209b
4.14
3.85
5
10
4
10
3
10
2
10
91.7
0.32
0
2
-10
3
4
5
0
10
10
10
12.1
0.31
5
10
4
10
3
10
2
10
86.8
0.79
0
2
-10
3
4
5
0
10
10
10
CD273/PD-L2
2.2
2.8
5
10
4
10
3
10
2
10
0
93.7
1.3
2
-10
3
4
5
0
10
10
10
2A1
cDC1
11.0
3.9
5
10
4
10
3
10
2
10
0
77.2
7.9
2
-10
3
4
5
0
10
10
10
cDC2
CD274/PD-L1
5.32
0.36
5
10
4
10
3
10
2
10
94.3
7.28E-3
0
2
-10
3
4
5
0
10
10
10
15.1
1.86
5
10
4
10
3
10
2
10
81.7
1.30
0
2
-10
3
4
5
0
10
10
10
CD301b
3.9
0.2
5
10
4
10
3
10
2
10
27.8
68.2
0
2
-10
3
4
5
0
10
10
10
11.7
0.03
5
10
4
10
3
10
2
10
87.5
0.8
0
2
-10
3
4
5
0
10
10
10
CD370/DNGR1
0.99
0.27
5
10
4
10
3
10
2
10
92.2
6.53
0
2
-10
3
4
5
0
10
10
10
4.03
12.1
5
10
4
10
3
10
2
10
19.7
64.1
0
2
-10
3
4
5
0
10
10
10
33D1
0.9
11.3
5
10
4
10
3
10
2
10
12.4
75.3
0
2
-10
3
4
5
0
10
10
10
3.1
8.4
5
10
4
10
3
10
2
10
45.1
43.4
0
2
-10
3
4
5
0
10
10
10
Zbtb46
4.5
2.0
5
10
4
10
3
10
2
10
59.0
34.5
0
2
-10
3
4
5
0
10
10
10
10.9
0.26
5
10
4
10
3
10
2
10
82.9
5.9
0
2
-10
3
4
5
0
10
10
10
XCR1
5.20
0.028
5
10
4
10
3
10
2
10
94.7
0.028
0
2
-10
3
4
5
0
10
10
10
10.1
0.81
5
10
4
10
3
10
2
10
80.4
8.72
0
2
-10
3
4
5
0
10
10
10
CD301a
2A1
cDC1
cDC2

## Slide 8
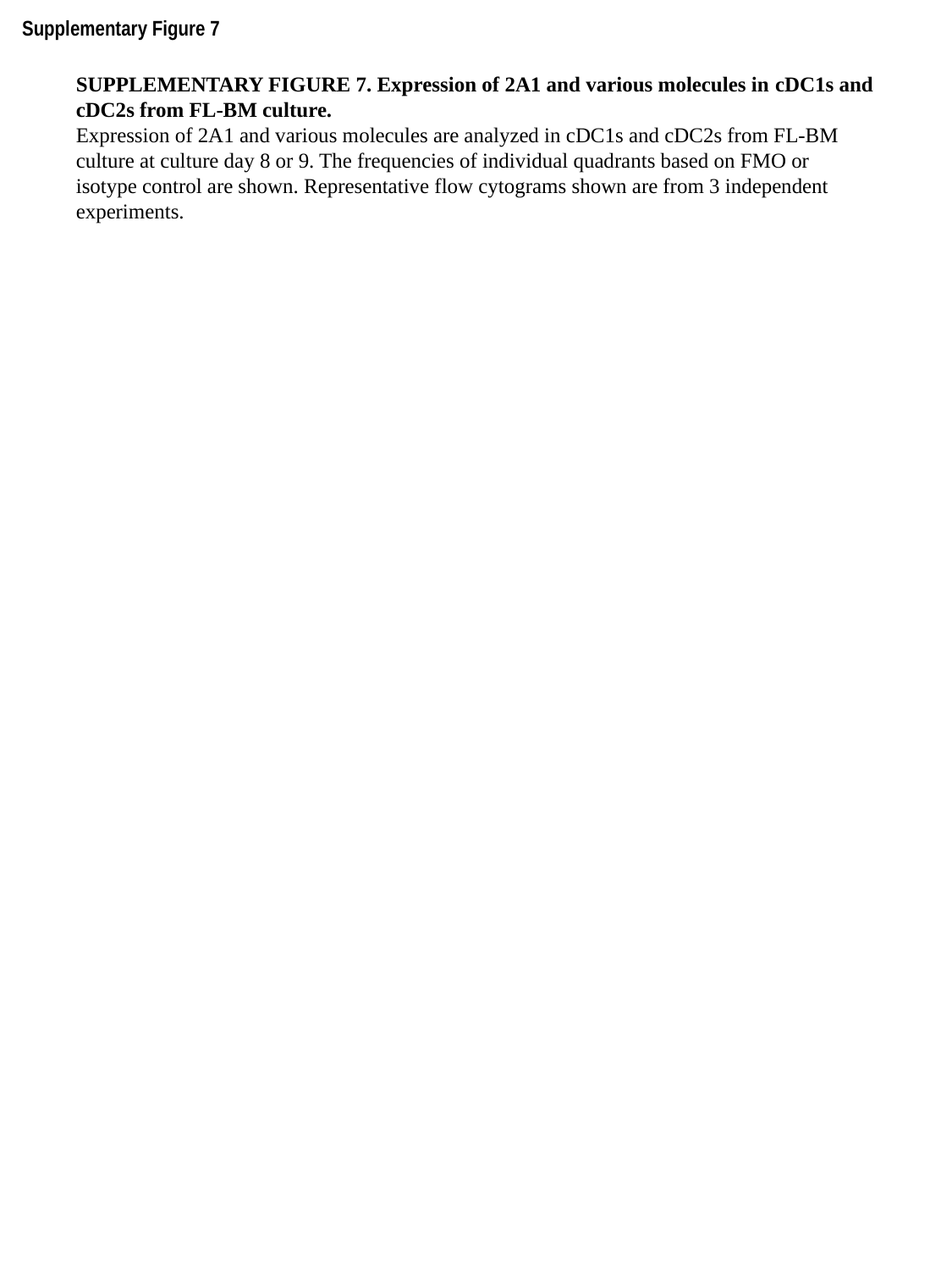

Supplementary Figure 7
SUPPLEMENTARY FIGURE 7. Expression of 2A1 and various molecules in cDC1s and cDC2s from FL-BM culture.
Expression of 2A1 and various molecules are analyzed in cDC1s and cDC2s from FL-BM culture at culture day 8 or 9. The frequencies of individual quadrants based on FMO or isotype control are shown. Representative flow cytograms shown are from 3 independent experiments.

## Slide 9
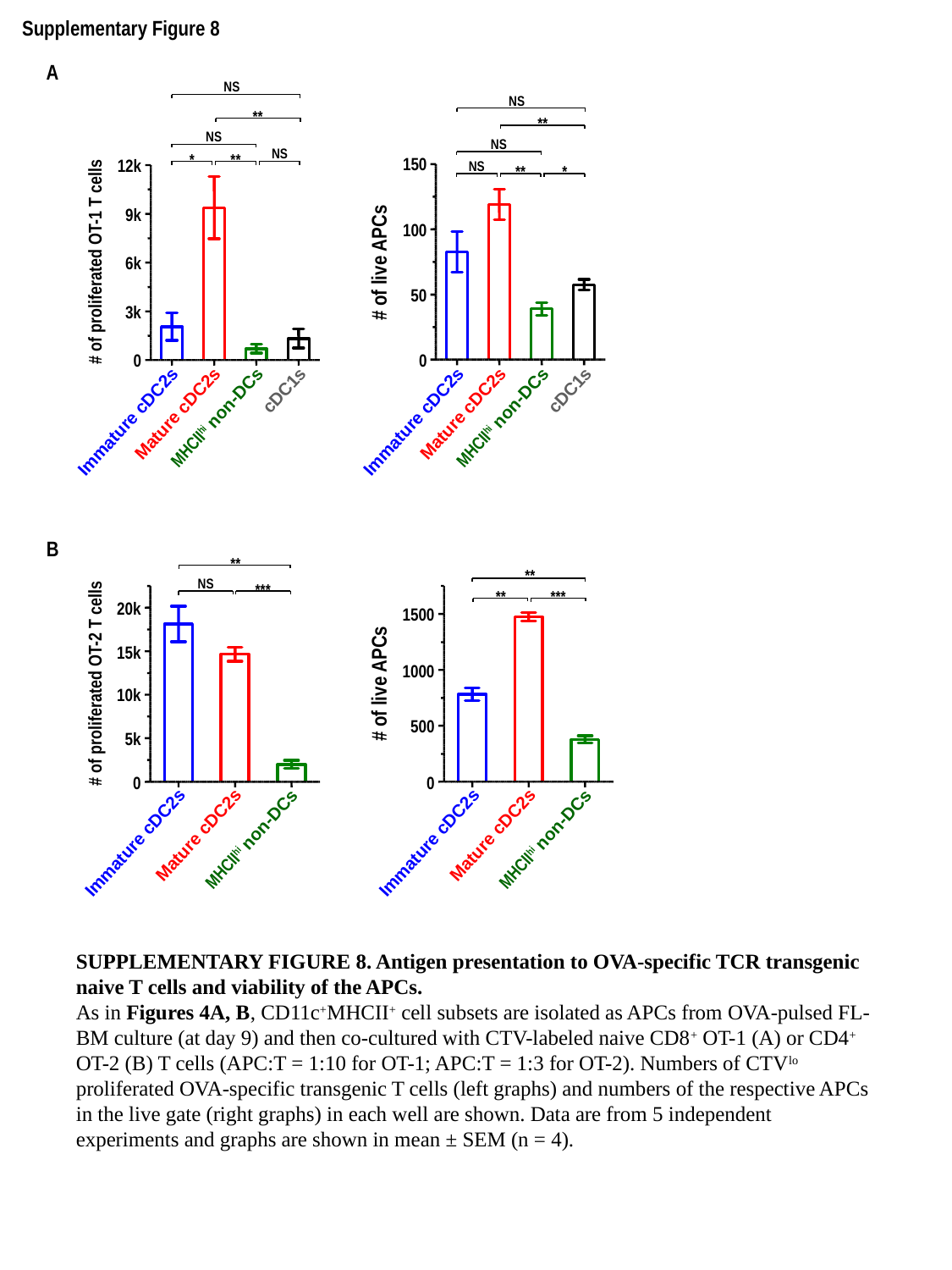

Supplementary Figure 8
A
NS
**
NS
NS
*
**
12k
9k
# of proliferated OT-1 T cells
6k
3k
0
cDC1s
Mature cDC2s
MHCIIhi non-DCs
Immature cDC2s
NS
**
NS
150
NS
**
*
100
# of live APCs
50
0
cDC1s
Mature cDC2s
MHCIIhi non-DCs
Immature cDC2s
B
**
NS
***
20k
15k
# of proliferated OT-2 T cells
10k
5k
0
Mature cDC2s
MHCIIhi non-DCs
Immature cDC2s
**
1500
1000
500
0
**
***
# of live APCs
Mature cDC2s
MHCIIhi non-DCs
Immature cDC2s
SUPPLEMENTARY FIGURE 8. Antigen presentation to OVA-specific TCR transgenic naive T cells and viability of the APCs.
As in Figures 4A, B, CD11c+MHCII+ cell subsets are isolated as APCs from OVA-pulsed FL-BM culture (at day 9) and then co-cultured with CTV-labeled naive CD8+ OT-1 (A) or CD4+ OT-2 (B) T cells (APC:T = 1:10 for OT-1; APC:T = 1:3 for OT-2). Numbers of CTVlo proliferated OVA-specific transgenic T cells (left graphs) and numbers of the respective APCs in the live gate (right graphs) in each well are shown. Data are from 5 independent experiments and graphs are shown in mean ± SEM (n = 4).

## Slide 10
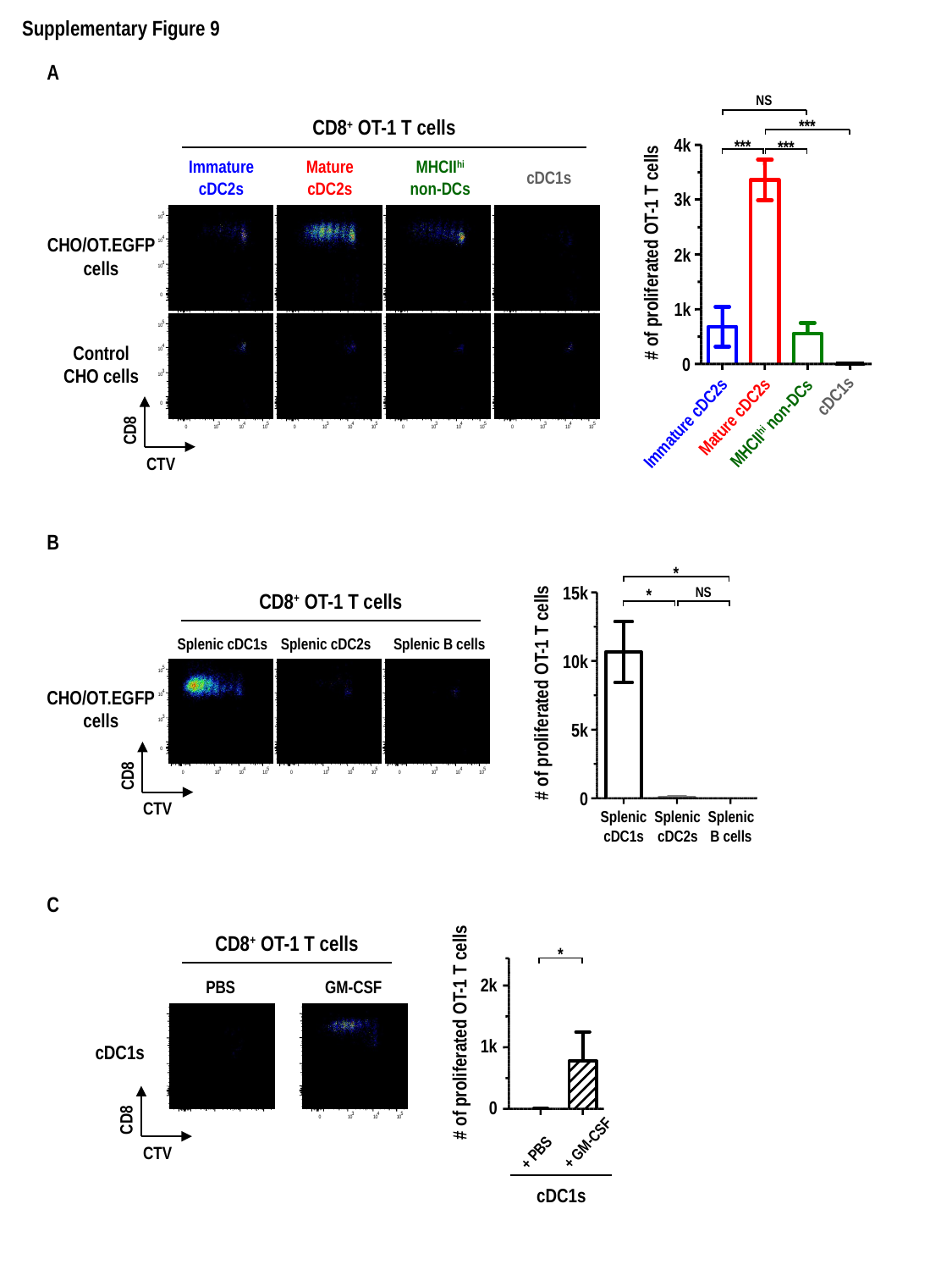

Supplementary Figure 9
A
NS
***
4k
3k
2k
1k
0
# of proliferated OT-1 T cells
***
***
cDC1s
Mature cDC2s
Immature cDC2s
MHCIIhi non-DCs
CD8+ OT-1 T cells
Immature
cDC2s
Mature
cDC2s
MHCIIhi
non-DCs
cDC1s
49.3
50.6
5
10
4
10
3
10
0
0
0.073
3
4
5
10
10
10
0
34.5
62.6
76.5
23.1
0
2.86
0.081
0.28
3
4
5
0
0
10
10
10
25.9
50.0
0
24.1
3
4
5
3
4
10
10
10
0
10
10
5
10
CHO/OT.EGFP
cells
4.00
92.8
0
0
3.20
3
4
5
3
4
5
0
10
10
10
10
10
10
5.38
86.0
2.50
95.0
1.08
7.53
0
2.50
3
4
5
0
10
10
10
0
0
92.6
0
7.41
3
4
5
10
10
10
0
5
10
4
10
3
10
Control
CHO cells
CD8
CTV
B
*
15k
NS
*
10k
# of proliferated OT-1 T cells
5k
0
Splenic
cDC1s
Splenic
cDC2s
Splenic
B cells
CD8+ OT-1 T cells
Splenic cDC1s
Splenic cDC2s
Splenic B cells
96.7
3.26
0
0
3
4
5
0
10
10
10
30.8
69.2
2.38
95.2
0
0
0
2.38
3
4
5
0
0
10
10
10
5
10
4
10
3
10
0
3
4
5
10
10
10
CHO/OT.EGFP
cells
CD8
CTV
C
CD8+ OT-1 T cells
PBS
GM-CSF
91.6
8.41
0
0
3
4
5
0
10
10
10
26.3
31.6
26.3
15.8
cDC1s
CD8
CTV
*
2k
1k
0
+ GM-CSF
+ PBS
cDC1s
# of proliferated OT-1 T cells

## Slide 11
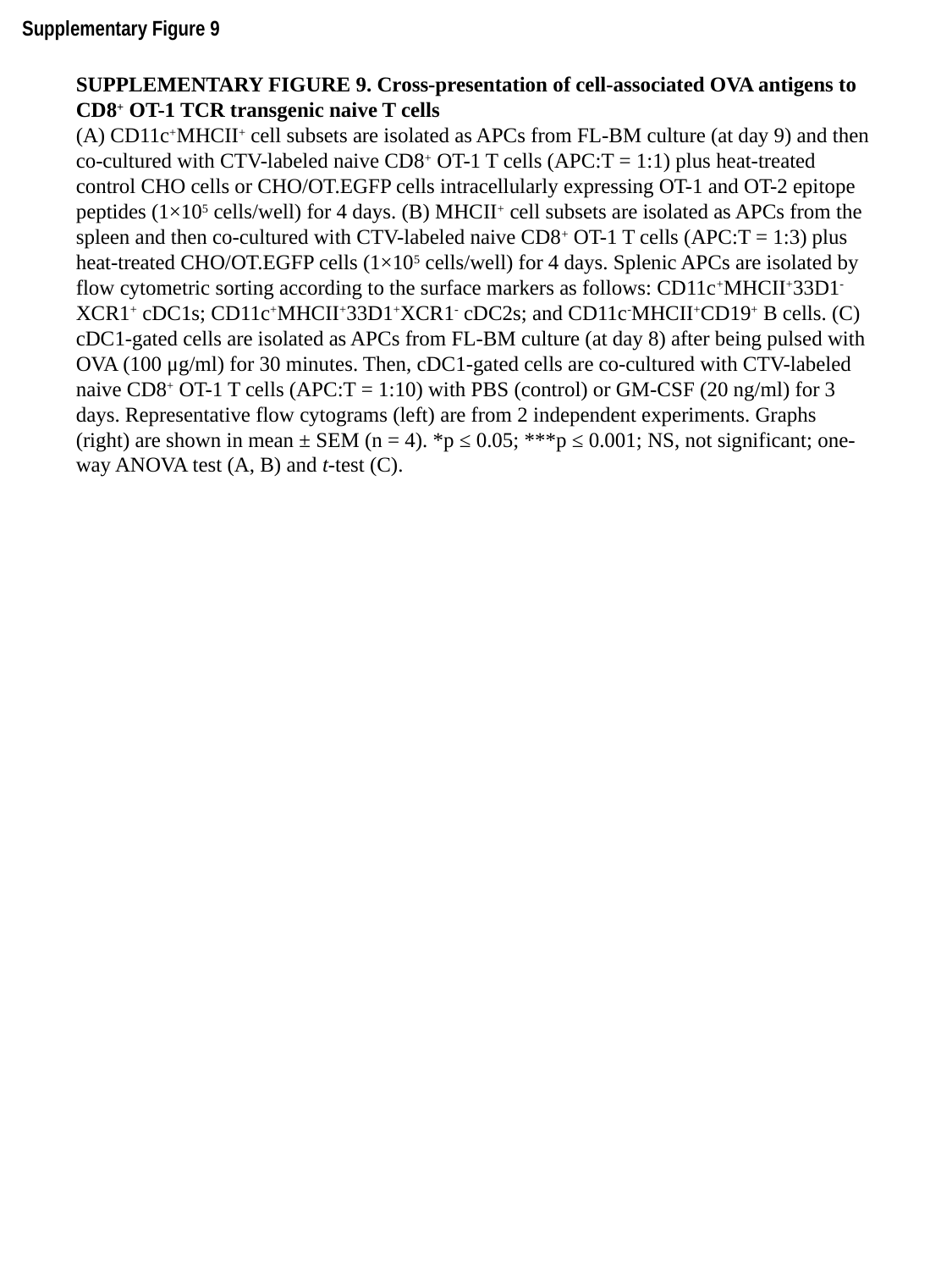

Supplementary Figure 9
SUPPLEMENTARY FIGURE 9. Cross-presentation of cell-associated OVA antigens to CD8+ OT-1 TCR transgenic naive T cells
(A) CD11c+MHCII+ cell subsets are isolated as APCs from FL-BM culture (at day 9) and then co-cultured with CTV-labeled naive CD8+ OT-1 T cells (APC:T = 1:1) plus heat-treated control CHO cells or CHO/OT.EGFP cells intracellularly expressing OT-1 and OT-2 epitope peptides (1×10^5^ cells/well) for 4 days. (B) MHCII+ cell subsets are isolated as APCs from the spleen and then co-cultured with CTV-labeled naive CD8+ OT-1 T cells (APC:T = 1:3) plus heat-treated CHO/OT.EGFP cells (1×10^5^ cells/well) for 4 days. Splenic APCs are isolated by flow cytometric sorting according to the surface markers as follows: CD11c+MHCII+33D1-XCR1+ cDC1s; CD11c+MHCII+33D1+XCR1- cDC2s; and CD11c-MHCII+CD19+ B cells. (C) cDC1-gated cells are isolated as APCs from FL-BM culture (at day 8) after being pulsed with OVA (100 μg/ml) for 30 minutes. Then, cDC1-gated cells are co-cultured with CTV-labeled naive CD8+ OT-1 T cells (APC:T = 1:10) with PBS (control) or GM-CSF (20 ng/ml) for 3 days. Representative flow cytograms (left) are from 2 independent experiments. Graphs (right) are shown in mean ± SEM (n = 4). *p ≤ 0.05; ***p ≤ 0.001; NS, not significant; one-way ANOVA test (A, B) and t-test (C).

## Slide 12
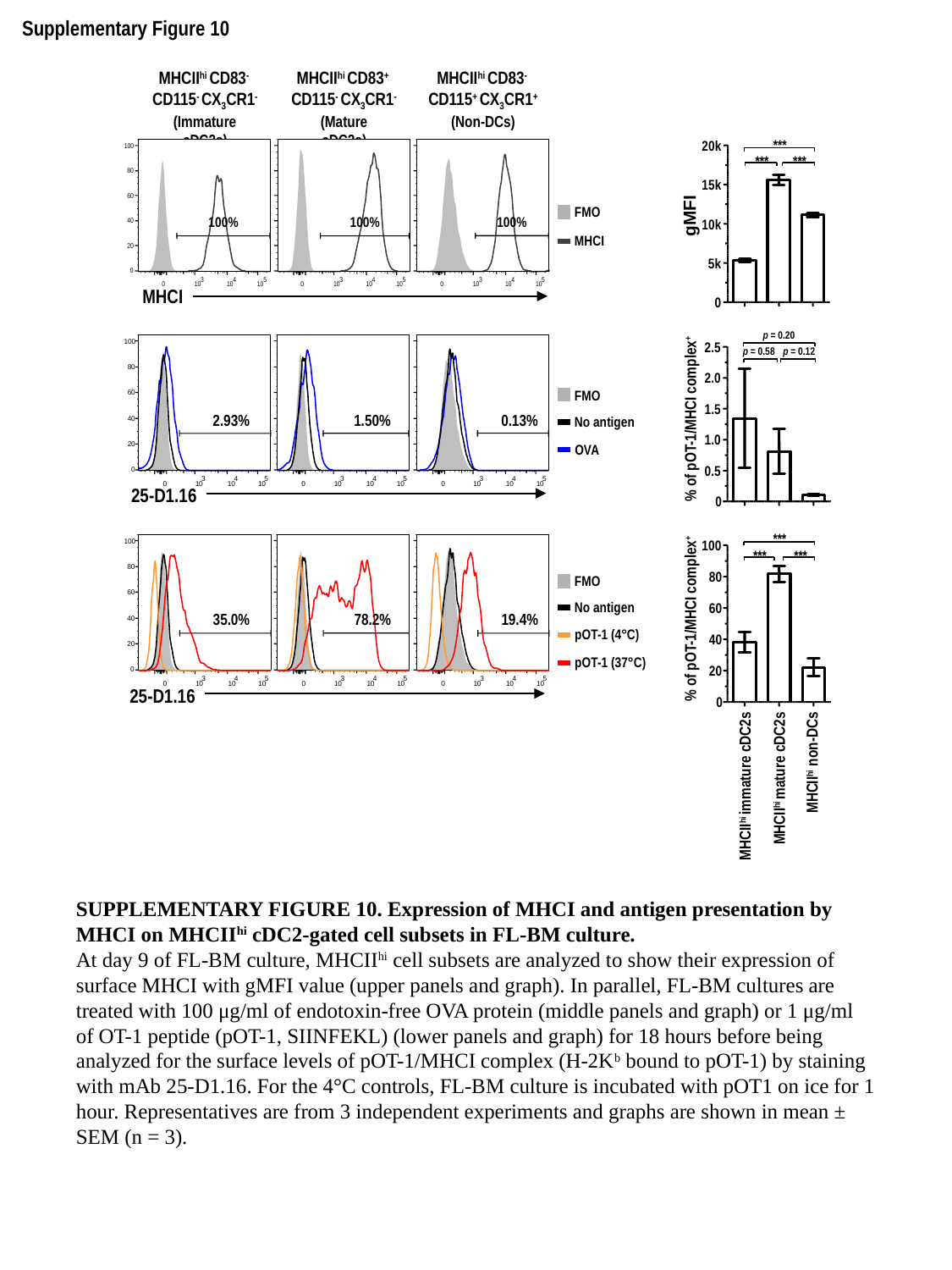

Supplementary Figure 10
MHCIIhi CD83-
CD115+ CX3CR1+
(Non-DCs)
MHCIIhi CD83+
CD115- CX3CR1-
(Mature cDC2s)
MHCIIhi CD83-
CD115- CX3CR1-
(Immature cDC2s)
***
***
***
20k
15k
gMFI
10k
5k
0
100
80
60
100%
40
20
0
3
4
5
0
10
10
10
100%
3
4
5
0
10
10
10
100%
3
4
5
0
10
10
10
MHCI
FMO
MHCI
p = 0.20
p = 0.58
p = 0.12
2.5
2.0
1.5
% of pOT-1/MHCI complex+
1.0
0.5
0
100
80
60
2.93%
40
20
0
3
4
5
0
10
10
10
1.50%
3
4
5
10
10
10
0
0.13%
3
4
5
0
10
10
10
25-D1.16
FMO
No antigen
OVA
***
100
***
***
80
60
% of pOT-1/MHCI complex+
40
20
0
100
80
60
35.0%
40
20
0
3
4
5
0
10
10
10
78.2%
3
4
5
0
10
10
10
19.4%
3
4
5
0
10
10
10
25-D1.16
FMO
No antigen
pOT-1 (4°C)
pOT-1 (37°C)
MHCIIhi non-DCs
MHCIIhi mature cDC2s
MHCIIhi immature cDC2s
SUPPLEMENTARY FIGURE 10. Expression of MHCI and antigen presentation by MHCI on MHCIIhi cDC2-gated cell subsets in FL-BM culture.
At day 9 of FL-BM culture, MHCIIhi cell subsets are analyzed to show their expression of surface MHCI with gMFI value (upper panels and graph). In parallel, FL-BM cultures are treated with 100 μg/ml of endotoxin-free OVA protein (middle panels and graph) or 1 μg/ml of OT-1 peptide (pOT-1, SIINFEKL) (lower panels and graph) for 18 hours before being analyzed for the surface levels of pOT-1/MHCI complex (H-2Kb bound to pOT-1) by staining with mAb 25-D1.16. For the 4°C controls, FL-BM culture is incubated with pOT1 on ice for 1 hour. Representatives are from 3 independent experiments and graphs are shown in mean ± SEM (n = 3).

## Slide 13
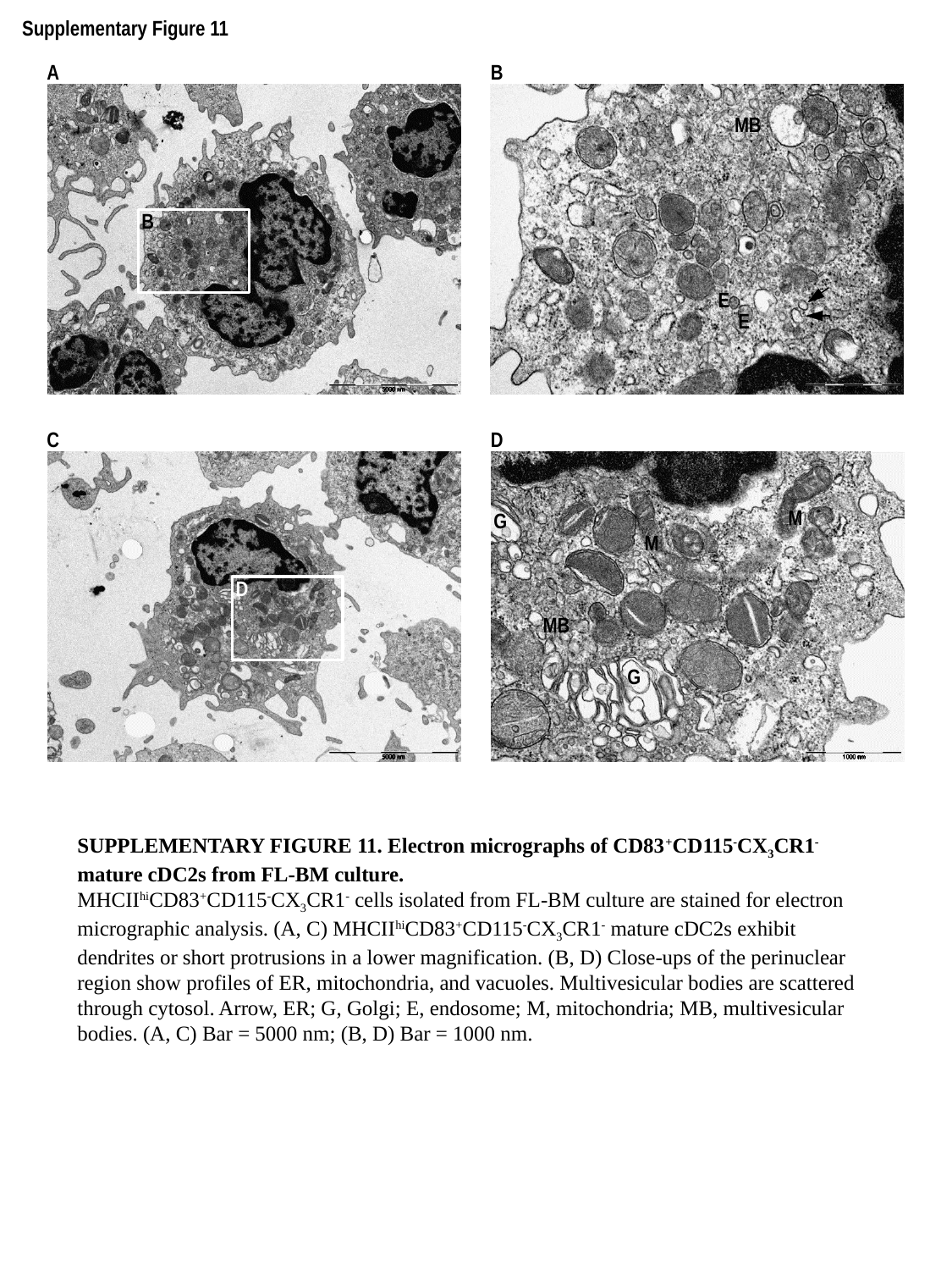

Supplementary Figure 11
A
B
MB
B
E
E
C
D
M
G
M
D
MB
G
SUPPLEMENTARY FIGURE 11. Electron micrographs of CD83+CD115-CX3CR1- mature cDC2s from FL-BM culture.
MHCIIhiCD83+CD115-CX3CR1- cells isolated from FL-BM culture are stained for electron micrographic analysis. (A, C) MHCIIhiCD83+CD115-CX3CR1- mature cDC2s exhibit dendrites or short protrusions in a lower magnification. (B, D) Close-ups of the perinuclear region show profiles of ER, mitochondria, and vacuoles. Multivesicular bodies are scattered through cytosol. Arrow, ER; G, Golgi; E, endosome; M, mitochondria; MB, multivesicular bodies. (A, C) Bar = 5000 nm; (B, D) Bar = 1000 nm.

## Slide 14
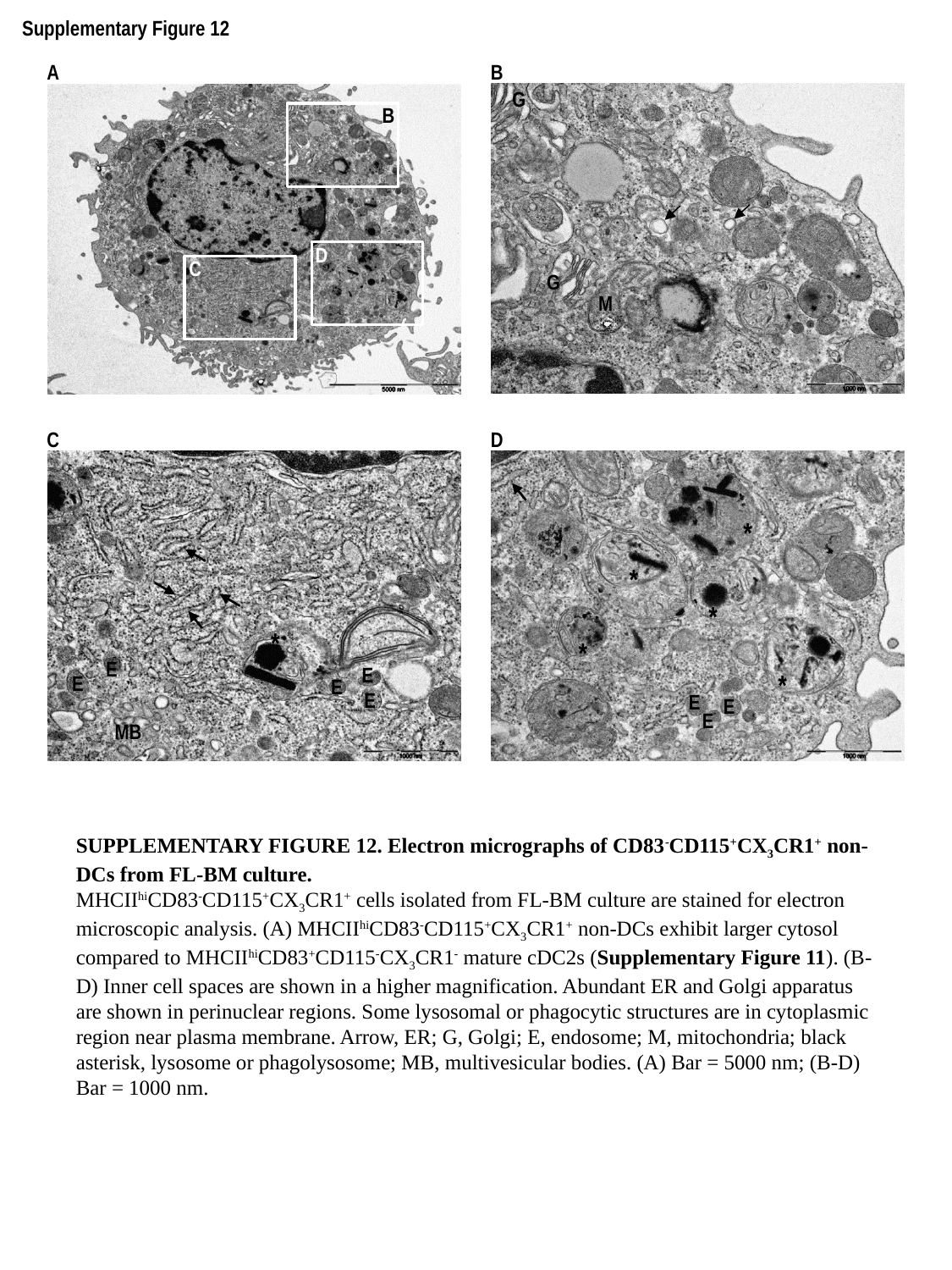

Supplementary Figure 12
A
B
G
B
D
C
G
M
C
D
*
*
*
*
*
E
E
*
E
E
E
E
E
E
MB
SUPPLEMENTARY FIGURE 12. Electron micrographs of CD83-CD115+CX3CR1+ non-DCs from FL-BM culture.
MHCIIhiCD83-CD115+CX3CR1+ cells isolated from FL-BM culture are stained for electron microscopic analysis. (A) MHCIIhiCD83-CD115+CX3CR1+ non-DCs exhibit larger cytosol compared to MHCIIhiCD83+CD115-CX3CR1- mature cDC2s (Supplementary Figure 11). (B-D) Inner cell spaces are shown in a higher magnification. Abundant ER and Golgi apparatus are shown in perinuclear regions. Some lysosomal or phagocytic structures are in cytoplasmic region near plasma membrane. Arrow, ER; G, Golgi; E, endosome; M, mitochondria; black asterisk, lysosome or phagolysosome; MB, multivesicular bodies. (A) Bar = 5000 nm; (B-D) Bar = 1000 nm.

## Slide 15
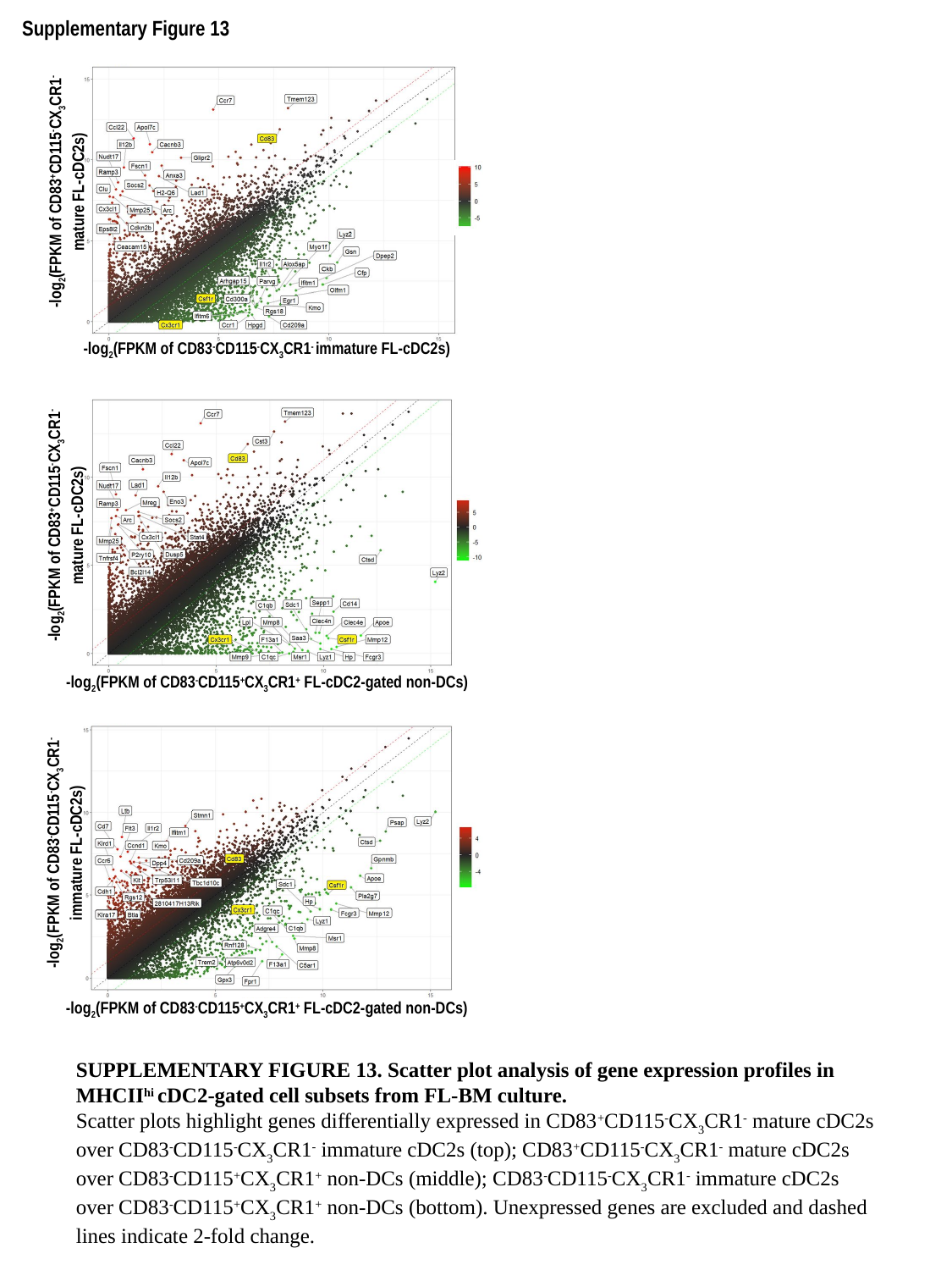

Supplementary Figure 13
-log2(FPKM of CD83+CD115-CX3CR1-
mature FL-cDC2s)
-log2(FPKM of CD83-CD115-CX3CR1- immature FL-cDC2s)
-log2(FPKM of CD83+CD115-CX3CR1-
mature FL-cDC2s)
-log2(FPKM of CD83-CD115+CX3CR1+ FL-cDC2-gated non-DCs)
-log2(FPKM of CD83-CD115-CX3CR1-
immature FL-cDC2s)
-log2(FPKM of CD83-CD115+CX3CR1+ FL-cDC2-gated non-DCs)
SUPPLEMENTARY FIGURE 13. Scatter plot analysis of gene expression profiles in MHCIIhi cDC2-gated cell subsets from FL-BM culture.
Scatter plots highlight genes differentially expressed in CD83+CD115-CX3CR1- mature cDC2s over CD83-CD115-CX3CR1- immature cDC2s (top); CD83+CD115-CX3CR1- mature cDC2s over CD83-CD115+CX3CR1+ non-DCs (middle); CD83-CD115-CX3CR1- immature cDC2s over CD83-CD115+CX3CR1+ non-DCs (bottom). Unexpressed genes are excluded and dashed lines indicate 2-fold change.

## Slide 16
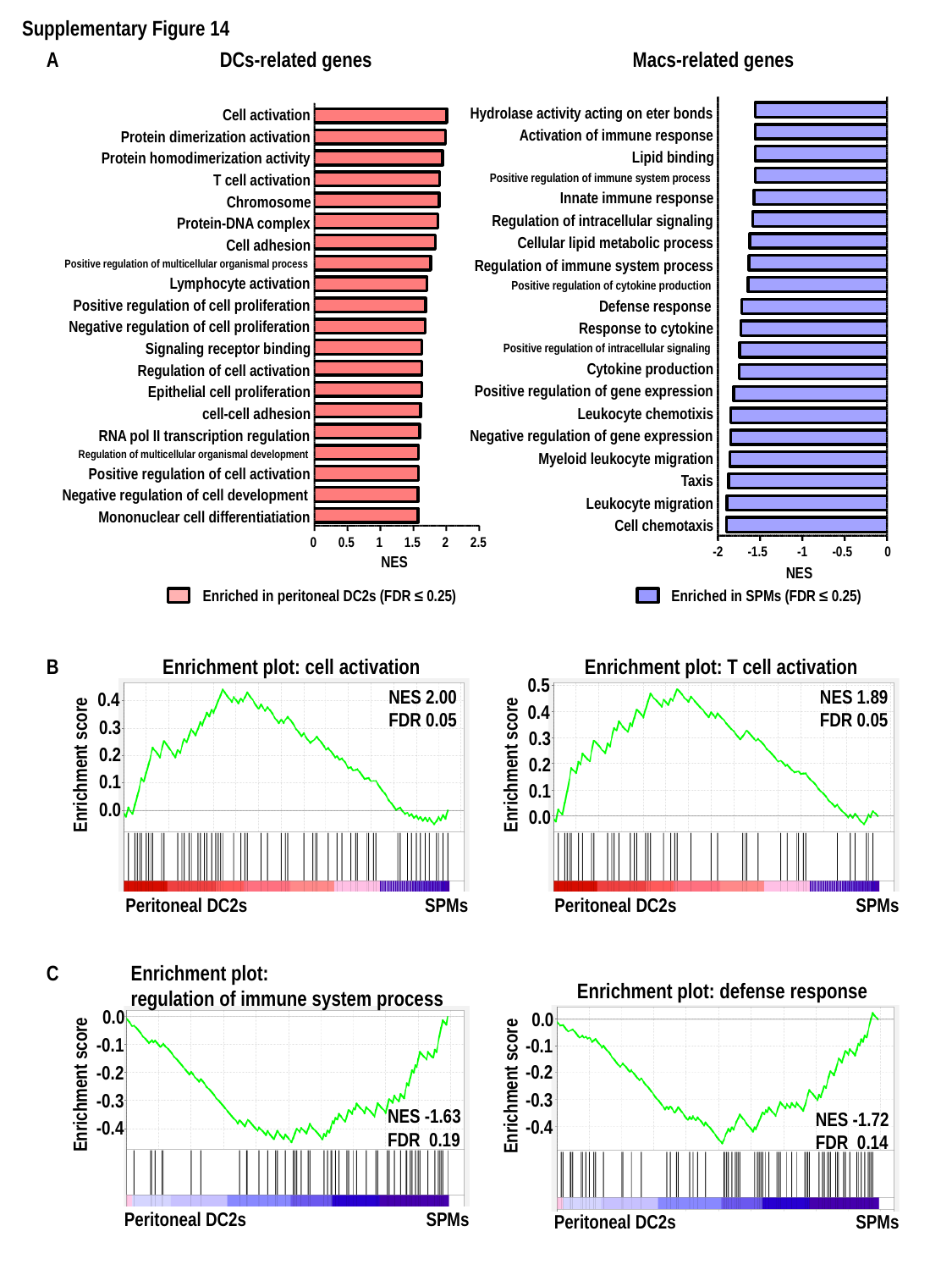

Supplementary Figure 14
A
DCs-related genes
Macs-related genes
Hydrolase activity acting on eter bonds
Activation of immune response
Lipid binding
Positive regulation of immune system process
Innate immune response
Regulation of intracellular signaling
Cellular lipid metabolic process
Regulation of immune system process
Positive regulation of cytokine production
Defense response
Response to cytokine
Positive regulation of intracellular signaling
Cytokine production
Positive regulation of gene expression
Leukocyte chemotixis
Negative regulation of gene expression
Myeloid leukocyte migration
Taxis
Leukocyte migration
Cell chemotaxis
-2
-1.5
-1
-0.5
0
NES
Cell activation
Protein dimerization activation
Protein homodimerization activity
T cell activation
Chromosome
Protein-DNA complex
Cell adhesion
Positive regulation of multicellular organismal process
Lymphocyte activation
Positive regulation of cell proliferation
Negative regulation of cell proliferation
Signaling receptor binding
Regulation of cell activation
Epithelial cell proliferation
cell-cell adhesion
RNA pol II transcription regulation
Regulation of multicellular organismal development
Positive regulation of cell activation
Negative regulation of cell development
Mononuclear cell differentiatiation
0
0.5
1
1.5
2
2.5
NES
Enriched in peritoneal DC2s (FDR ≤ 0.25)
Enriched in SPMs (FDR ≤ 0.25)
B
Enrichment plot: T cell activation
Enrichment plot: cell activation
0.5
NES 1.89
FDR 0.05
NES 2.00
FDR 0.05
0.4
0.4
0.3
0.3
0.2
0.2
Enrichment score
Enrichment score
0.1
0.1
0.0
0.0
Peritoneal DC2s
SPMs
Peritoneal DC2s
SPMs
C
Enrichment plot:
regulation of immune system process
Enrichment plot: defense response
0.0
0.0
-0.1
-0.1
-0.2
-0.2
Enrichment score
Enrichment score
-0.3
-0.3
NES -1.63
FDR 0.19
NES -1.72
FDR 0.14
-0.4
-0.4
Peritoneal DC2s
SPMs
Peritoneal DC2s
SPMs

## Slide 17
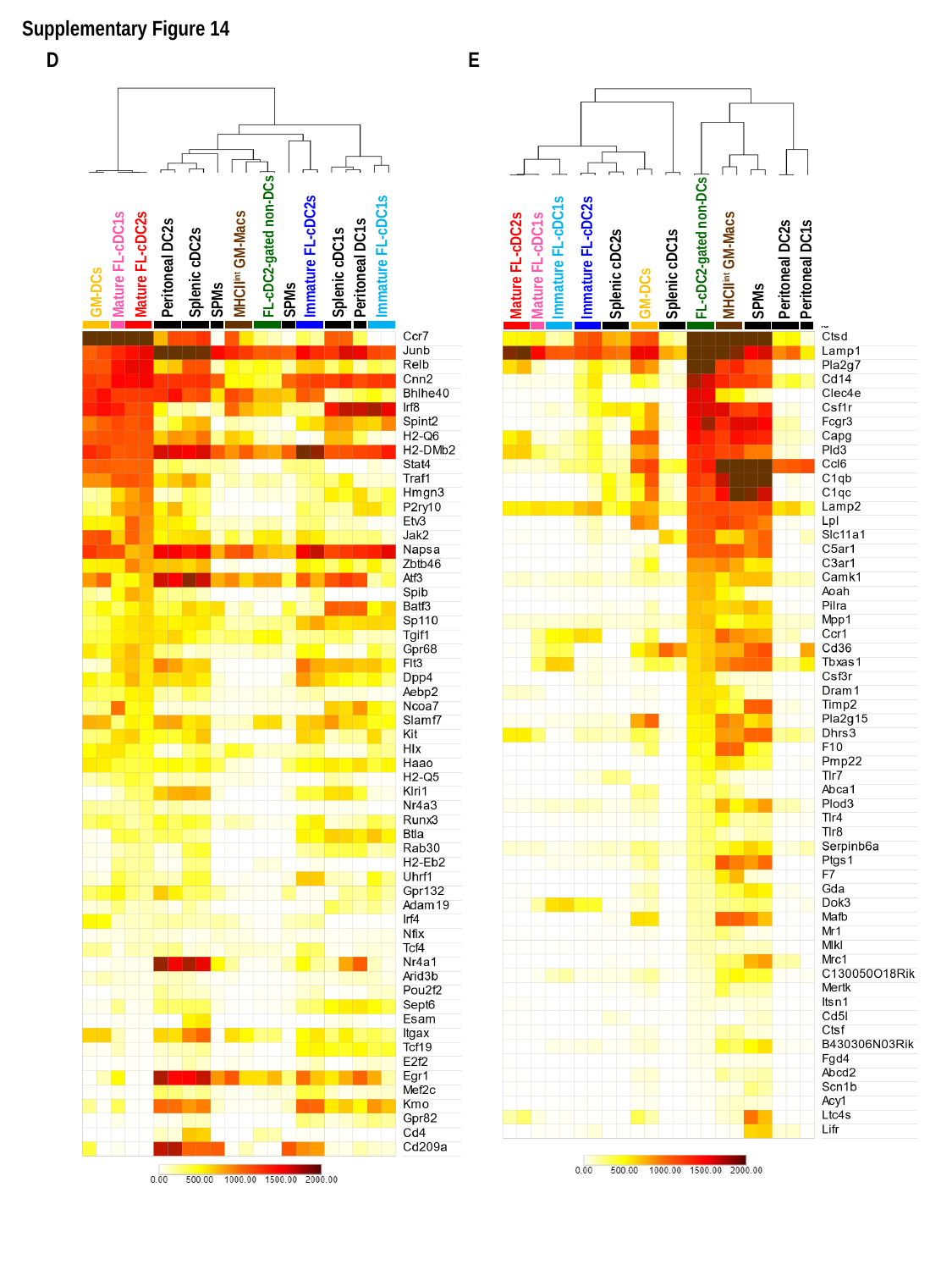

Supplementary Figure 14
D
E
FL-cDC2-gated non-DCs
Mature FL-cDC2s
Mature FL-cDC1s
Immature FL-cDC1s
Immature FL-cDC2s
Splenic cDC2s
GM-DCs
Splenic cDC1s
MHCIIint GM-Macs
SPMs
Peritoneal DC2s
Peritoneal DC1s
FL-cDC2-gated non-DCs
GM-DCs
Mature FL-cDC1s
Mature FL-cDC2s
Peritoneal DC2s
Splenic cDC2s
SPMs
MHCIIint GM-Macs
SPMs
Immature FL-cDC2s
Splenic cDC1s
Peritoneal DC1s
Immature FL-cDC1s

## Slide 18
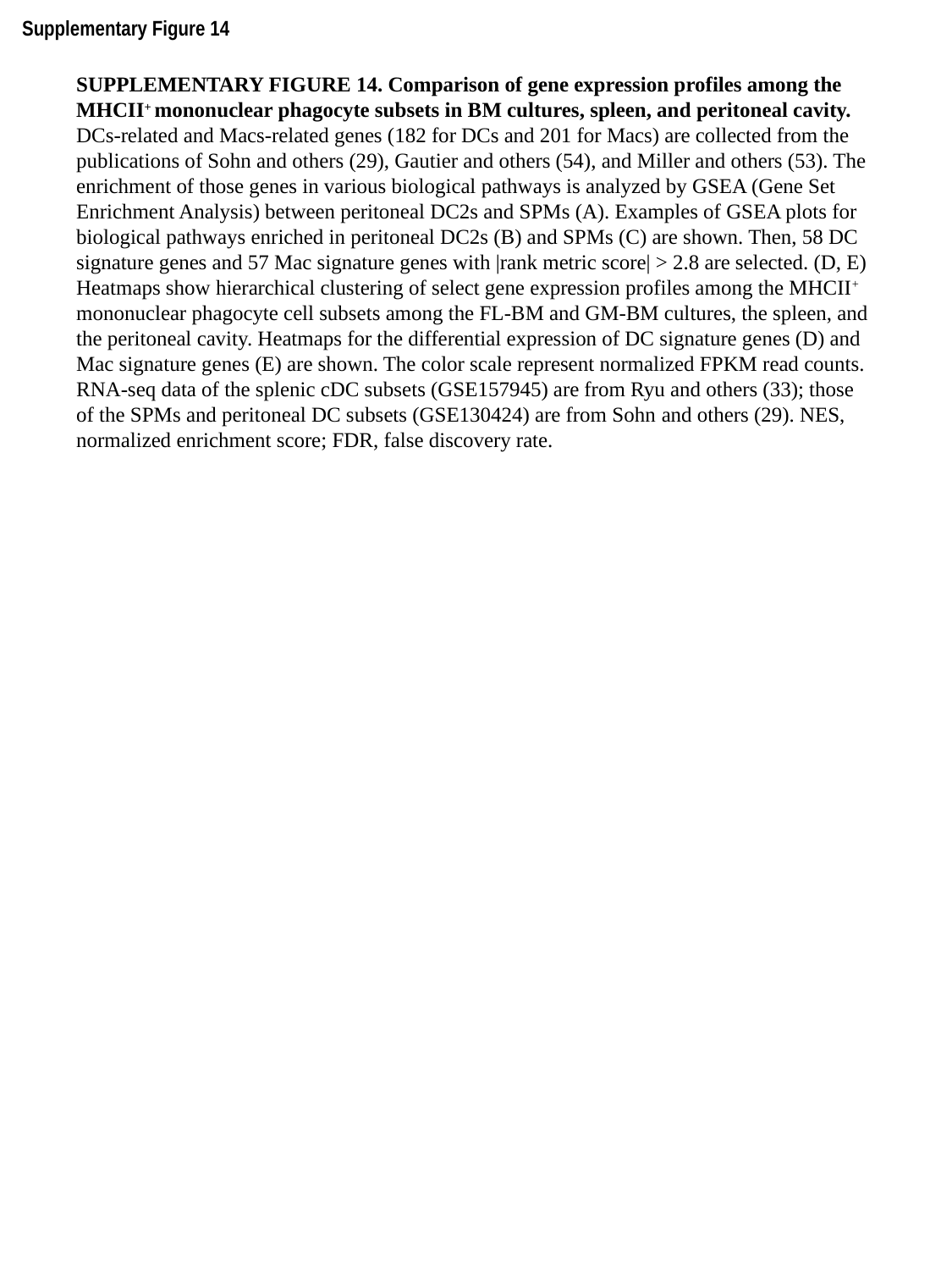

Supplementary Figure 14
SUPPLEMENTARY FIGURE 14. Comparison of gene expression profiles among the MHCII+ mononuclear phagocyte subsets in BM cultures, spleen, and peritoneal cavity.
DCs-related and Macs-related genes (182 for DCs and 201 for Macs) are collected from the publications of Sohn and others (29), Gautier and others (54), and Miller and others (53). The enrichment of those genes in various biological pathways is analyzed by GSEA (Gene Set Enrichment Analysis) between peritoneal DC2s and SPMs (A). Examples of GSEA plots for biological pathways enriched in peritoneal DC2s (B) and SPMs (C) are shown. Then, 58 DC signature genes and 57 Mac signature genes with |rank metric score| > 2.8 are selected. (D, E) Heatmaps show hierarchical clustering of select gene expression profiles among the MHCII+ mononuclear phagocyte cell subsets among the FL-BM and GM-BM cultures, the spleen, and the peritoneal cavity. Heatmaps for the differential expression of DC signature genes (D) and Mac signature genes (E) are shown. The color scale represent normalized FPKM read counts. RNA-seq data of the splenic cDC subsets (GSE157945) are from Ryu and others (33); those of the SPMs and peritoneal DC subsets (GSE130424) are from Sohn and others (29). NES, normalized enrichment score; FDR, false discovery rate.

## Slide 19
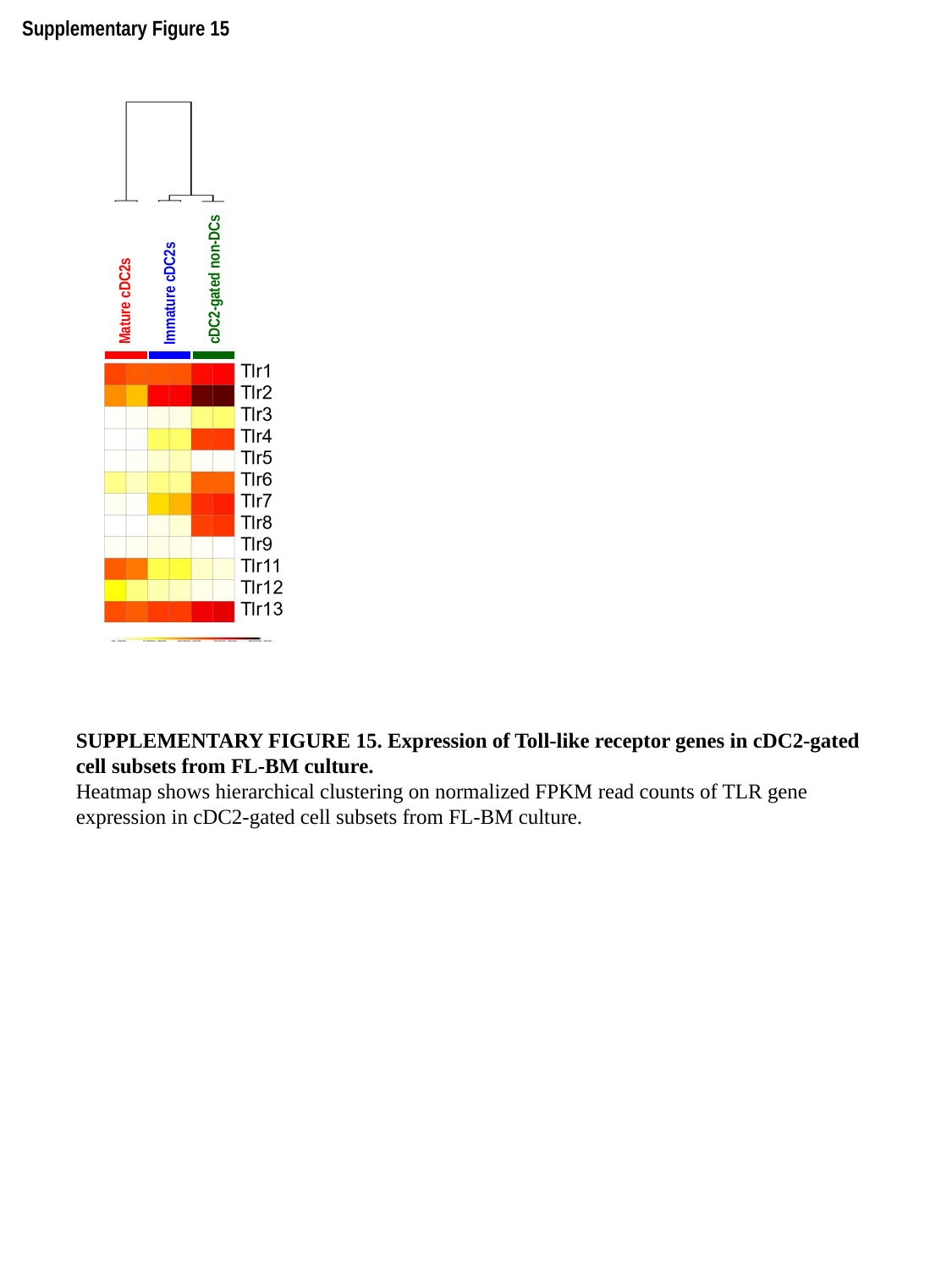

Supplementary Figure 15
cDC2-gated non-DCs
Mature cDC2s
Immature cDC2s
SUPPLEMENTARY FIGURE 15. Expression of Toll-like receptor genes in cDC2-gated cell subsets from FL-BM culture.
Heatmap shows hierarchical clustering on normalized FPKM read counts of TLR gene expression in cDC2-gated cell subsets from FL-BM culture.

## Slide 20
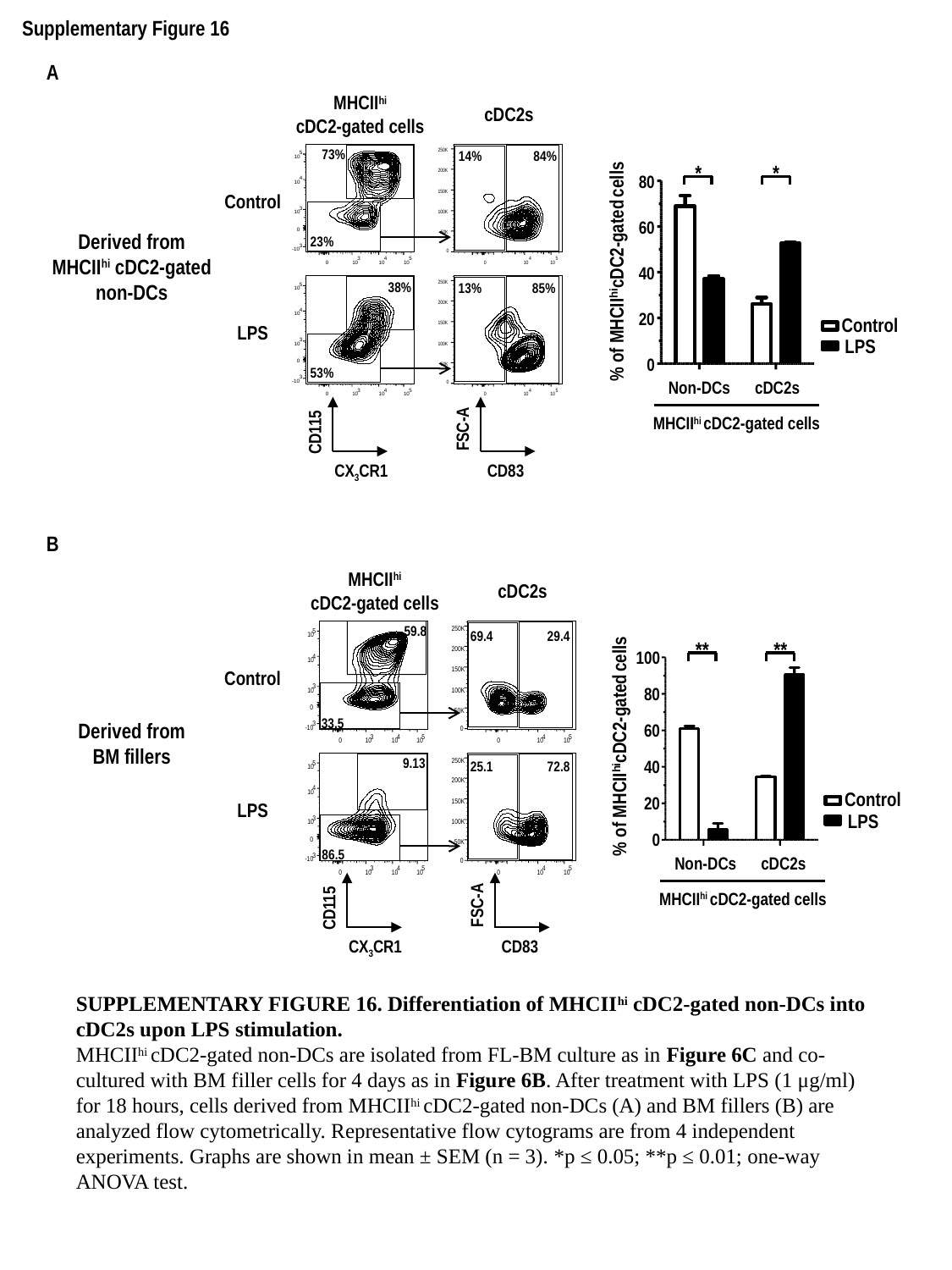

Supplementary Figure 16
A
MHCIIhi
cDC2-gated cells
cDC2s
73%
5
10
4
10
3
10
0
23%
3
-10
3
4
5
0
10
10
10
250K
14%
84%
200K
150K
100K
50K
0
4
5
0
10
10
38%
5
10
4
10
3
10
0
53%
3
-10
3
4
5
0
10
10
10
250K
13%
85%
200K
150K
100K
50K
0
4
5
0
10
10
Control
LPS
FSC-A
CD83
CD115
CX3CR1
*
*
80
60
40
20
0
% of MHCIIhicDC2-gated cells
Control
LPS
Non-DCs
cDC2s
MHCIIhi cDC2-gated cells
Derived from
MHCIIhi cDC2-gated
non-DCs
B
MHCIIhi
cDC2-gated cells
cDC2s
59.8
5
10
4
10
3
10
0
33.5
3
-10
3
4
5
0
10
10
10
250K
69.4
29.4
200K
150K
100K
50K
0
4
5
0
10
10
9.13
5
10
4
10
3
10
0
86.5
3
-10
3
4
5
0
10
10
10
250K
25.1
72.8
200K
150K
100K
50K
0
4
5
0
10
10
Control
LPS
**
**
100
80
60
40
20
0
% of MHCIIhicDC2-gated cells
Control
LPS
Non-DCs
cDC2s
MHCIIhi cDC2-gated cells
FSC-A
CD83
CD115
CX3CR1
Derived from
BM fillers
SUPPLEMENTARY FIGURE 16. Differentiation of MHCIIhi cDC2-gated non-DCs into cDC2s upon LPS stimulation.
MHCIIhi cDC2-gated non-DCs are isolated from FL-BM culture as in Figure 6C and co-cultured with BM filler cells for 4 days as in Figure 6B. After treatment with LPS (1 μg/ml) for 18 hours, cells derived from MHCIIhi cDC2-gated non-DCs (A) and BM fillers (B) are analyzed flow cytometrically. Representative flow cytograms are from 4 independent experiments. Graphs are shown in mean ± SEM (n = 3). *p ≤ 0.05; **p ≤ 0.01; one-way ANOVA test.

## Slide 21
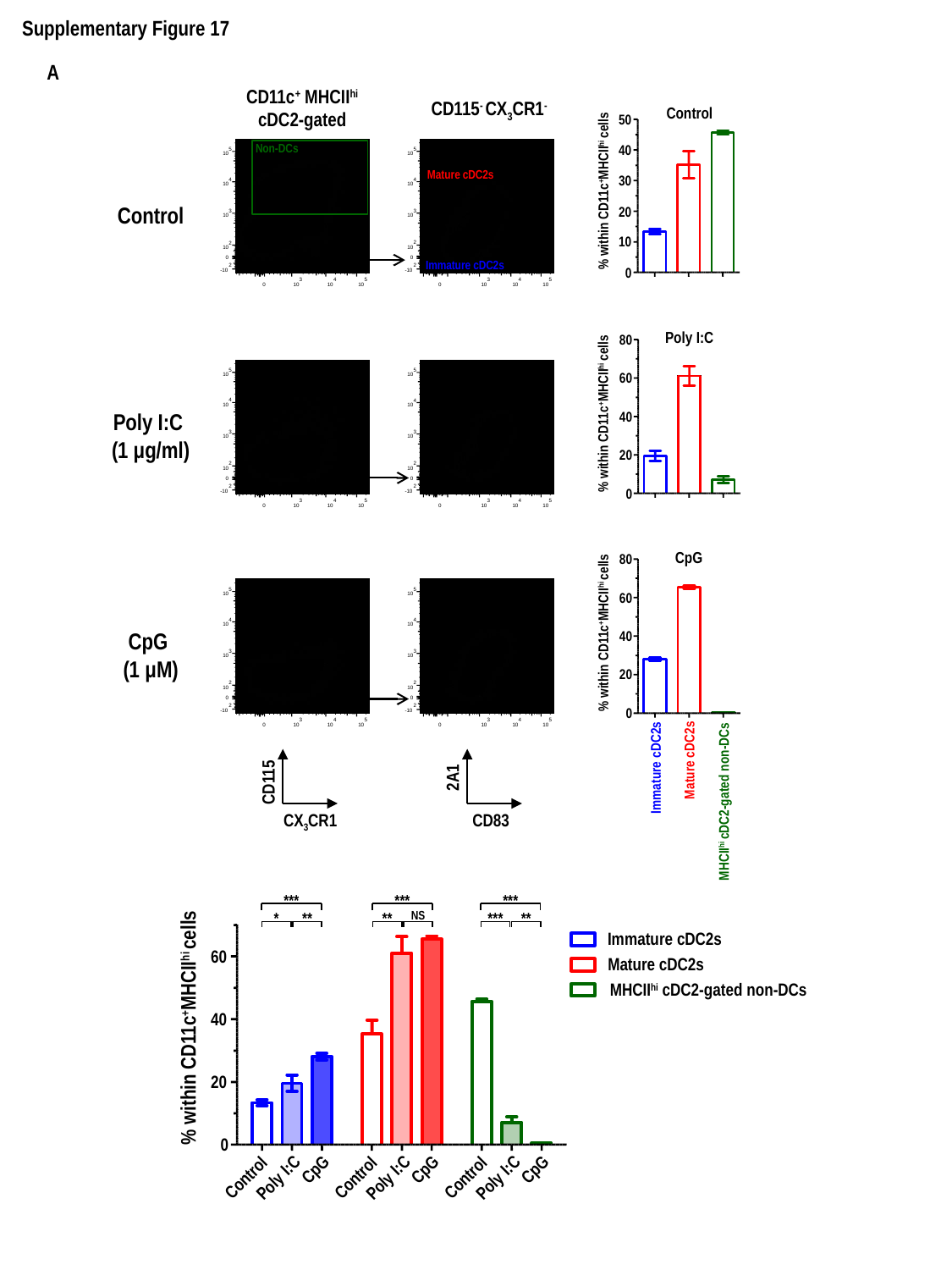

Supplementary Figure 17
A
CD11c+ MHCIIhi
cDC2-gated
CD115- CX3CR1-
Control
50
40
30
20
10
0
% within CD11c+MHCIIhi cells
46%
5
10
4
10
3
10
2
10
51%
0
2
-10
3
4
5
0
10
10
10
5
10
72%
4
10
3
10
26%
2
10
0
2
-10
3
4
5
0
10
10
10
Non-DCs
Mature cDC2s
Control
Immature cDC2s
Poly I:C
80
60
40
20
0
% within CD11c+MHCIIhi cells
3.0%
5
10
4
10
3
10
2
10
0
96%
2
-10
3
4
5
0
10
10
10
5
10
90%
4
10
3
10
8.2%
2
10
0
2
-10
3
4
5
0
10
10
10
Poly I:C
(1 μg/ml)
CpG
80
60
40
20
0
Mature cDC2s
Immature cDC2s
MHCIIhi cDC2-gated non-DCs
% within CD11c+MHCIIhi cells
0.73%
5
10
4
10
3
10
2
10
0
98%
2
-10
3
4
5
0
10
10
10
5
10
72%
4
10
3
10
25%
2
10
0
2
-10
3
4
5
0
10
10
10
CpG
(1 μM)
CD115
CX3CR1
2A1
CD83
***
*
**
***
NS
**
***
***
**
60
40
% within CD11c+MHCIIhi cells
20
0
CpG
CpG
CpG
Poly I:C
Poly I:C
Control
Control
Control
Poly I:C
Immature cDC2s
Mature cDC2s
MHCIIhi cDC2-gated non-DCs

## Slide 22
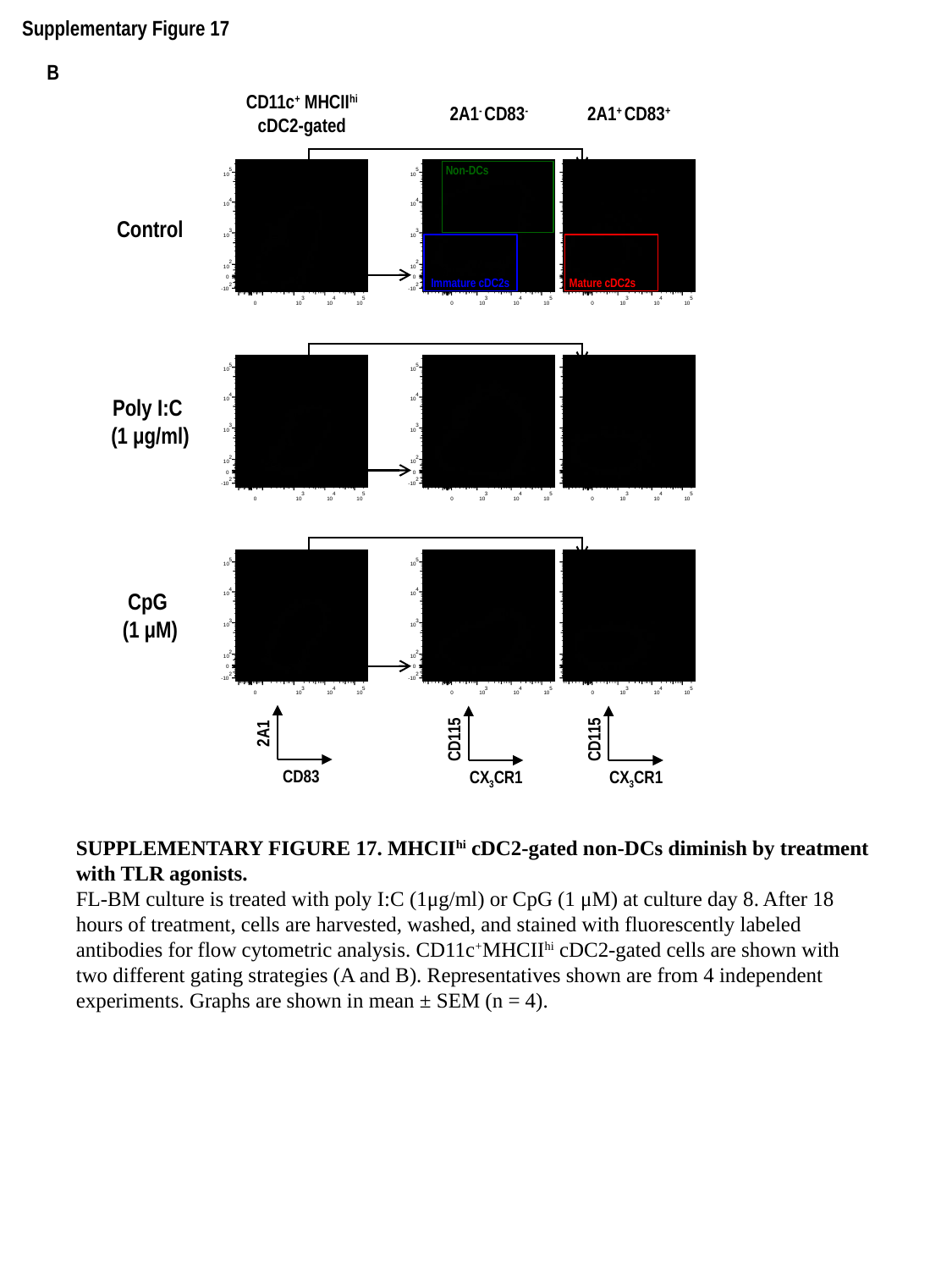

Supplementary Figure 17
B
CD11c+ MHCIIhi
cDC2-gated
2A1- CD83-
2A1+ CD83+
5
10
36%
4
10
3
10
61%
2
10
0
2
-10
3
4
5
0
10
10
10
71%
5
10
4
10
3
10
2
26%
10
0
2
-10
3
4
5
0
10
10
10
96%
3
4
5
0
10
10
10
Non-DCs
Control
Immature cDC2s
Mature cDC2s
5
10
79%
4
10
3
10
18%
2
10
0
2
-10
3
4
5
0
10
10
10
21%
5
10
4
10
3
10
2
10
72%
0
2
-10
3
4
5
0
10
10
10
99%
3
4
5
0
10
10
10
Poly I:C
(1 μg/ml)
5
10
58%
4
10
3
10
36%
2
10
0
2
-10
3
4
5
0
10
10
10
3.9%
5
10
4
10
3
10
2
10
91%
0
2
-10
3
4
5
0
10
10
10
99%
3
4
5
0
10
10
10
CpG
(1 μM)
CD115
CX3CR1
CD115
CX3CR1
2A1
CD83
SUPPLEMENTARY FIGURE 17. MHCIIhi cDC2-gated non-DCs diminish by treatment with TLR agonists.
FL-BM culture is treated with poly I:C (1μg/ml) or CpG (1 μM) at culture day 8. After 18 hours of treatment, cells are harvested, washed, and stained with fluorescently labeled antibodies for flow cytometric analysis. CD11c+MHCIIhi cDC2-gated cells are shown with two different gating strategies (A and B). Representatives shown are from 4 independent experiments. Graphs are shown in mean ± SEM (n = 4).

## Slide 23
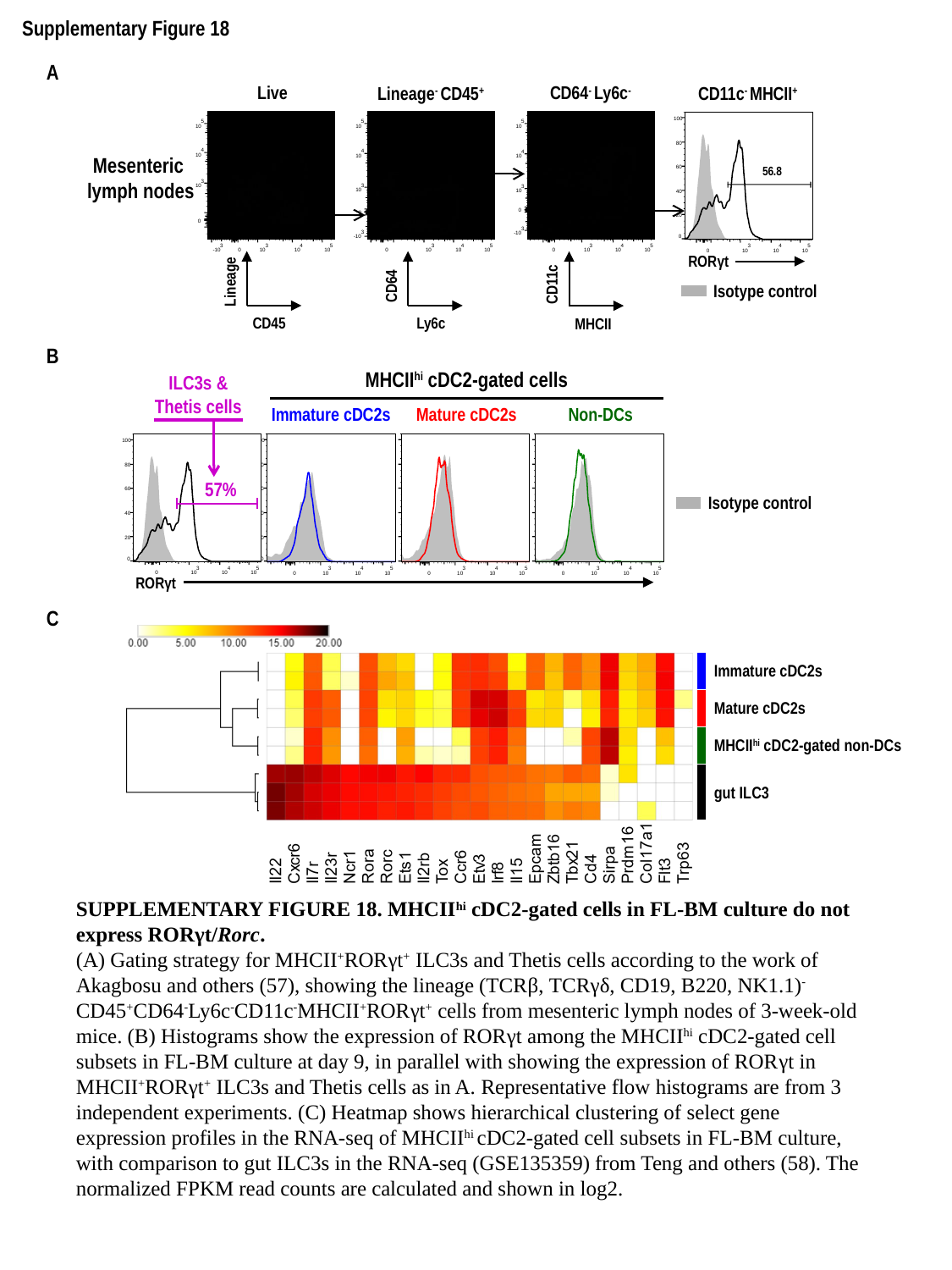

Supplementary Figure 18
A
Live
CD64- Ly6c-
Lineage- CD45+
CD11c- MHCII+
5
10
4
10
3
10
0
9.50
3
3
4
5
-10
0
10
10
10
5
10
4
10
58.1
3
10
0
3
-10
3
4
5
0
10
10
10
5
10
4
10
3
10
0
8.81
3
-10
3
4
5
0
10
10
10
100
80
56.8
60
40
20
0
3
4
5
0
10
10
10
Mesenteric
lymph nodes
Lineage
CD45
RORγt
CD11c
MHCII
CD64
Ly6c
Isotype control
B
MHCIIhi cDC2-gated cells
ILC3s &
Thetis cells
Immature cDC2s
Mature cDC2s
Non-DCs
100
80
60
40
20
0
3
4
5
0
10
10
10
4
5
3
10
10
0
10
3
4
5
0
10
10
10
100
80
57%
60
40
20
0
3
4
5
0
10
10
10
Isotype control
RORγt
C
Immature cDC2s
Mature cDC2s
MHCIIhi cDC2-gated non-DCs
gut ILC3
SUPPLEMENTARY FIGURE 18. MHCIIhi cDC2-gated cells in FL-BM culture do not express RORγt/Rorc.
(A) Gating strategy for MHCII+RORγt+ ILC3s and Thetis cells according to the work of Akagbosu and others (57), showing the lineage (TCRβ, TCRγδ, CD19, B220, NK1.1)-CD45+CD64-Ly6c-CD11c-MHCII+RORγt+ cells from mesenteric lymph nodes of 3-week-old mice. (B) Histograms show the expression of RORγt among the MHCIIhi cDC2-gated cell subsets in FL-BM culture at day 9, in parallel with showing the expression of RORγt in MHCII+RORγt+ ILC3s and Thetis cells as in A. Representative flow histograms are from 3 independent experiments. (C) Heatmap shows hierarchical clustering of select gene expression profiles in the RNA-seq of MHCIIhi cDC2-gated cell subsets in FL-BM culture, with comparison to gut ILC3s in the RNA-seq (GSE135359) from Teng and others (58). The normalized FPKM read counts are calculated and shown in log2.

## Slide 24
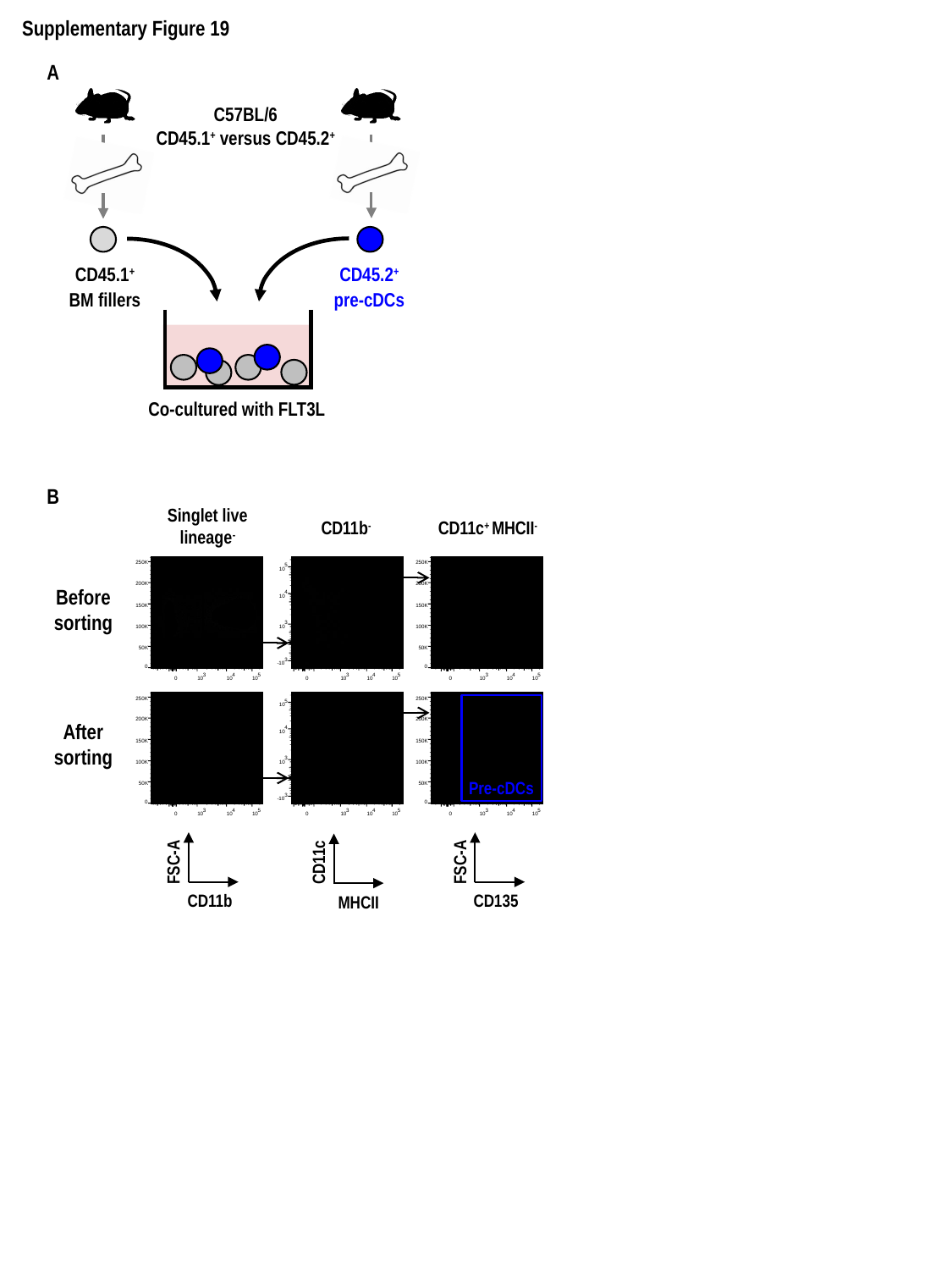

Supplementary Figure 19
A
C57BL/6
CD45.1+ versus CD45.2+
CD45.1+
BM fillers
CD45.2+
pre-cDCs
Co-cultured with FLT3L
B
Singlet live
lineage-
CD11b-
CD11c+ MHCII-
250K
12.6
86.3
200K
150K
100K
50K
0
3
4
5
0
10
10
10
6.66
5
10
4
10
3
10
0
3
-10
3
4
5
0
10
10
10
250K
78.1
200K
150K
100K
50K
0
3
4
5
0
10
10
10
Before sorting
250K
96.7
2.22
200K
150K
100K
50K
0
3
4
5
0
10
10
10
97.7
5
10
4
10
3
10
0
3
-10
3
4
5
0
10
10
10
250K
98.8
200K
150K
100K
50K
0
3
4
5
0
10
10
10
After sorting
Pre-cDCs
CD11c
MHCII
FSC-A
CD135
FSC-A
CD11b

## Slide 25
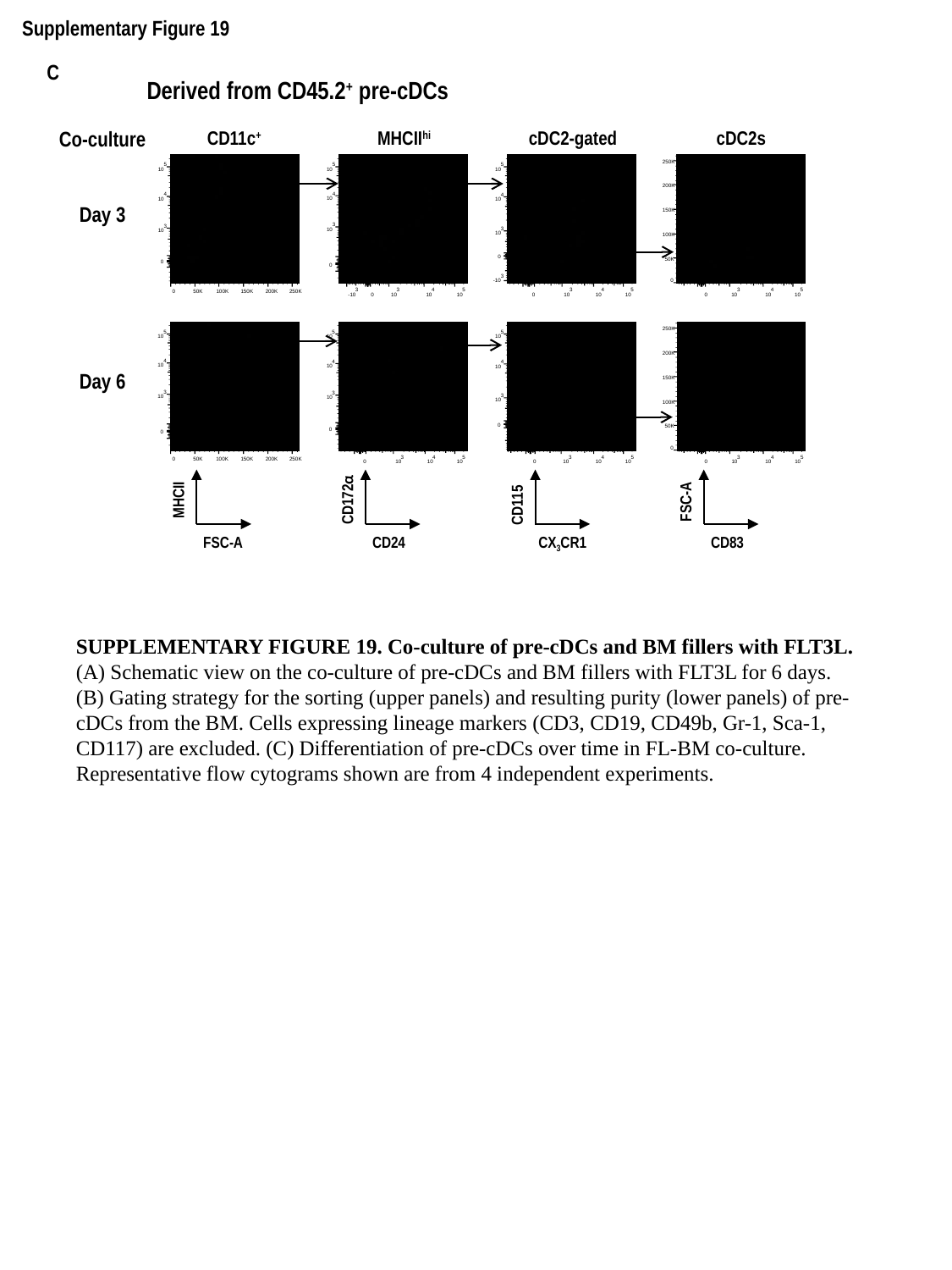

Supplementary Figure 19
C
Derived from CD45.2+ pre-cDCs
Co-culture
Day 3
Day 6
CD11c+
MHCIIhi
cDC2-gated
cDC2s
5
85.6
10
4
10
3
10
0
0
50K
100K
150K
200K
250K
97.3
5
10
4
10
3
10
0
3
3
4
5
-10
0
10
10
10
5
53.6
10
4
10
3
10
0
44.0
3
-10
3
4
5
0
10
10
10
250K
22.9
69.2
200K
150K
100K
50K
0
3
4
5
0
10
10
10
85.8
5
10
4
10
3
10
0
0
50K
100K
150K
200K
250K
5
10
99.9
4
10
3
10
0
3
4
5
0
10
10
10
5
10
68.9
4
10
3
10
0
24.3
3
4
5
0
10
10
10
250K
73.7
26.3
200K
150K
100K
50K
0
3
4
5
0
10
10
10
CD172α
CD24
MHCII
FSC-A
FSC-A
CD83
CD115
CX3CR1
SUPPLEMENTARY FIGURE 19. Co-culture of pre-cDCs and BM fillers with FLT3L.
(A) Schematic view on the co-culture of pre-cDCs and BM fillers with FLT3L for 6 days.
(B) Gating strategy for the sorting (upper panels) and resulting purity (lower panels) of pre-cDCs from the BM. Cells expressing lineage markers (CD3, CD19, CD49b, Gr-1, Sca-1, CD117) are excluded. (C) Differentiation of pre-cDCs over time in FL-BM co-culture. Representative flow cytograms shown are from 4 independent experiments.

## Slide 26
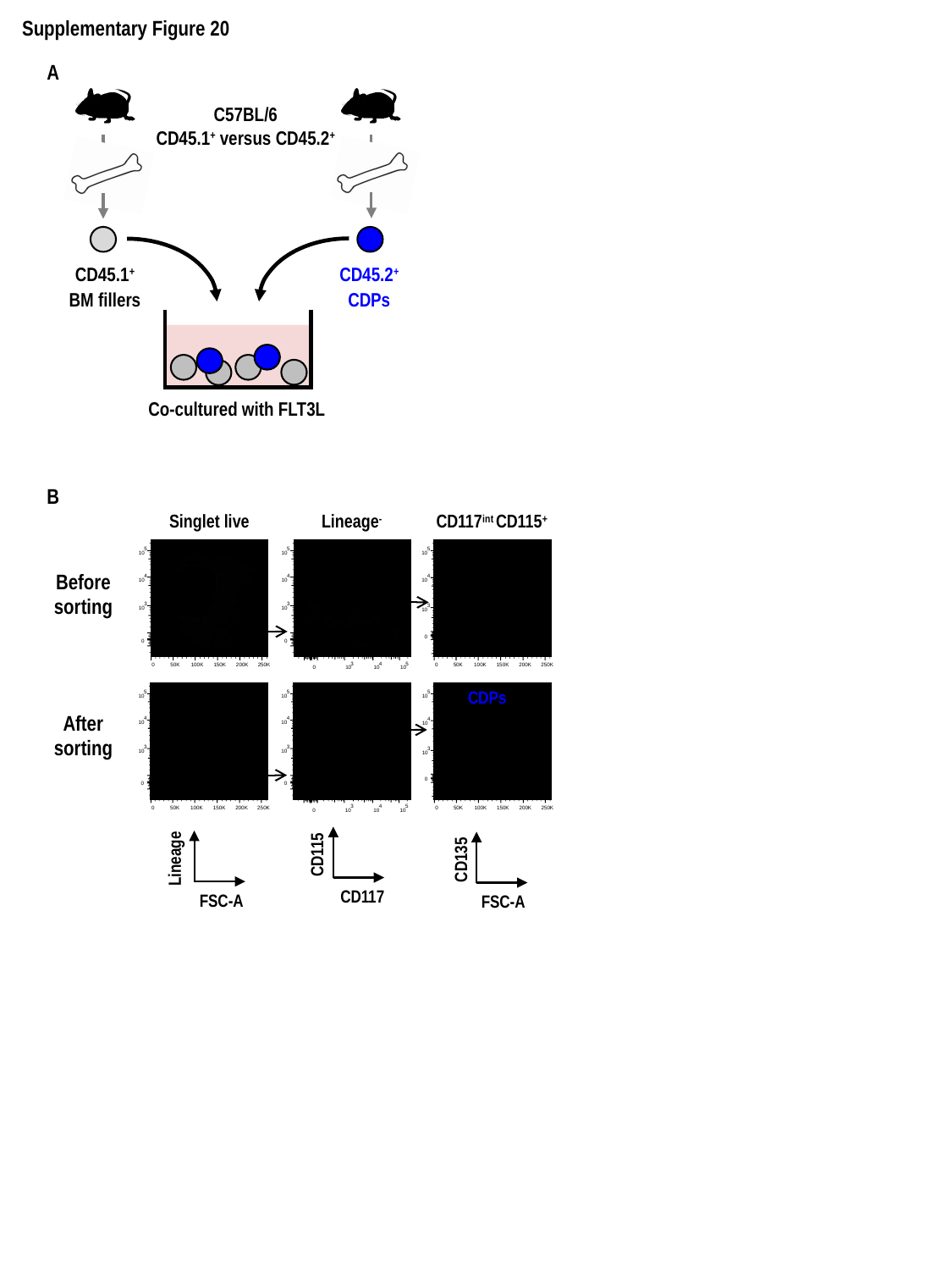

Supplementary Figure 20
A
C57BL/6
CD45.1+ versus CD45.2+
CD45.1+
BM fillers
CD45.2+
CDPs
Co-cultured with FLT3L
B
Singlet live
Lineage-
CD117int CD115+
5
10
4
10
3
10
11.2
0
0
50K
100K
150K
200K
250K
5
10
2.53
4
10
3
10
0
3
4
5
0
10
10
10
34.6
5
10
4
10
3
10
0
0
50K
100K
150K
200K
250K
Before sorting
CDPs
5
10
4
10
3
10
97.4
0
0
50K
100K
150K
200K
250K
5
10
76.3
4
10
3
10
0
3
4
5
0
10
10
10
100
5
10
4
10
3
10
0
0
50K
100K
150K
200K
250K
CDPs
After sorting
Lineage
FSC-A
CD115
CD117
CD135
FSC-A

## Slide 27
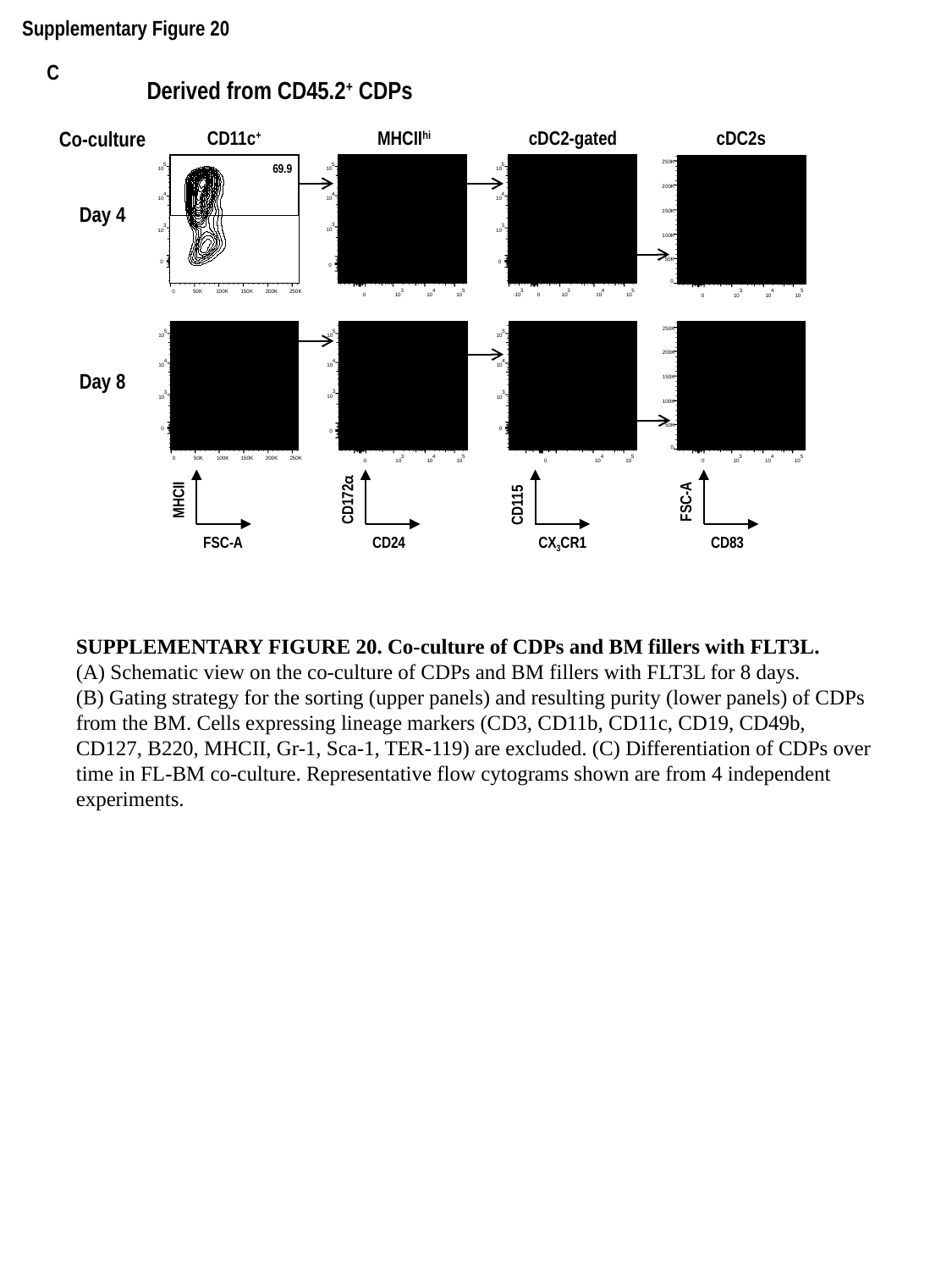

Supplementary Figure 20
C
Derived from CD45.2+ CDPs
Co-culture
Day 4
Day 8
CD11c+
MHCIIhi
cDC2-gated
cDC2s
5
69.9
10
4
10
3
10
0
0
50K
100K
150K
200K
250K
98.3
5
10
4
10
3
10
0
3
4
5
0
10
10
10
5
80.8
10
4
10
3
10
0
16.6
3
3
4
5
-10
0
10
10
10
250K
11.1
87.0
200K
150K
100K
50K
0
3
4
5
0
10
10
10
39.0
5
10
4
10
3
10
0
0
50K
100K
150K
200K
250K
98.1
5
10
4
10
3
10
0
3
4
5
0
10
10
10
86.3
5
10
4
10
3
10
0
11.7
4
5
0
10
10
250K
66.7
25.0
200K
150K
100K
50K
0
3
4
5
0
10
10
10
CD172α
CD24
MHCII
FSC-A
FSC-A
CD83
CD115
CX3CR1
SUPPLEMENTARY FIGURE 20. Co-culture of CDPs and BM fillers with FLT3L.
(A) Schematic view on the co-culture of CDPs and BM fillers with FLT3L for 8 days.
(B) Gating strategy for the sorting (upper panels) and resulting purity (lower panels) of CDPs from the BM. Cells expressing lineage markers (CD3, CD11b, CD11c, CD19, CD49b, CD127, B220, MHCII, Gr-1, Sca-1, TER-119) are excluded. (C) Differentiation of CDPs over time in FL-BM co-culture. Representative flow cytograms shown are from 4 independent experiments.

## Slide 28
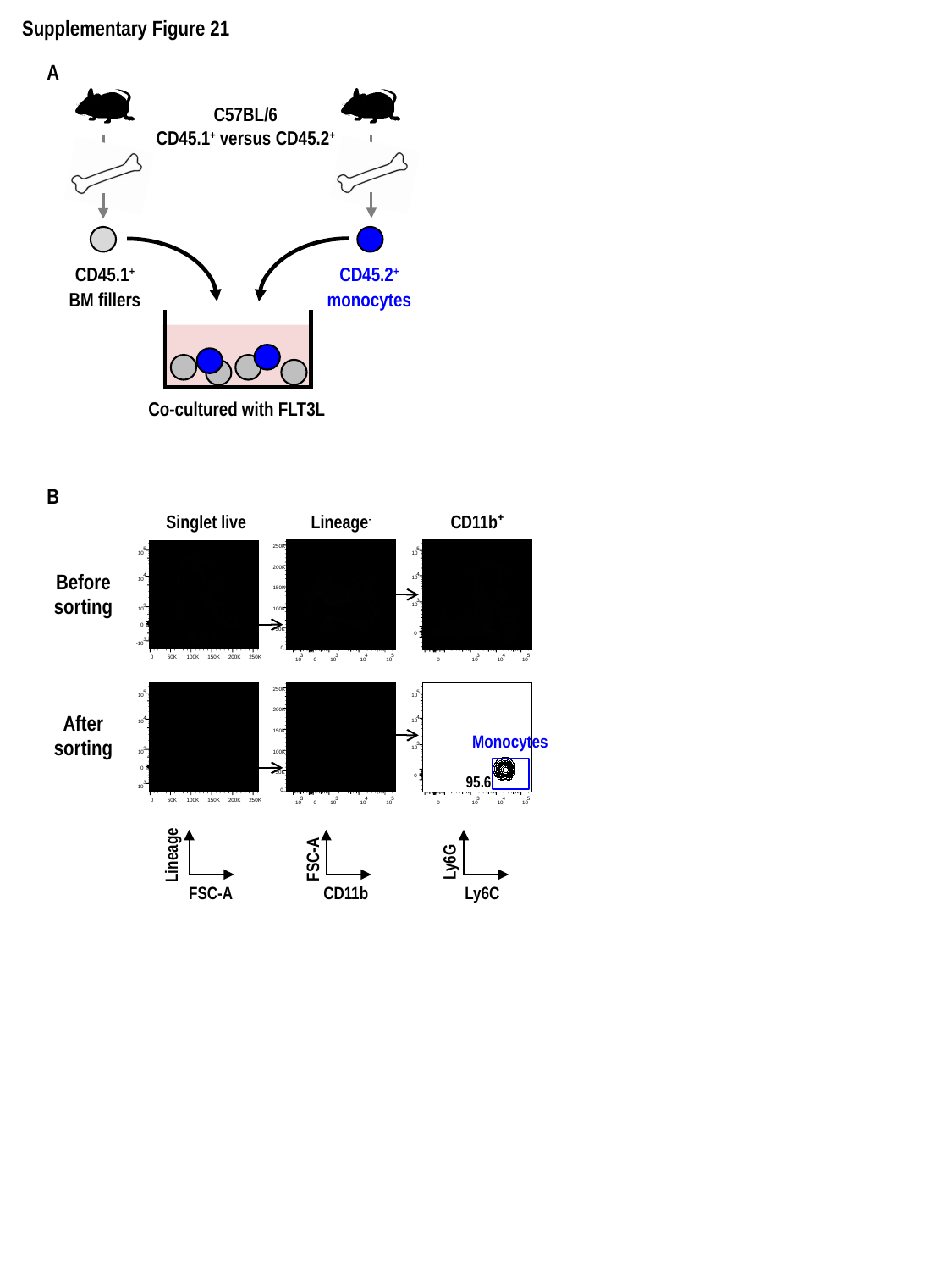

Supplementary Figure 21
A
C57BL/6
CD45.1+ versus CD45.2+
CD45.1+
BM fillers
CD45.2+
monocytes
Co-cultured with FLT3L
B
Singlet live
Lineage-
CD11b⁺
250K
57.0
200K
150K
100K
50K
0
5
3
3
4
-10
0
10
10
10
5
10
4
10
3
10
0
16.8
5
3
4
0
10
10
10
5
10
4
10
3
10
0
65.2
3
-10
0
50K
100K
150K
200K
250K
Before sorting
5
10
4
10
3
10
0
99.2
3
-10
0
50K
100K
150K
200K
250K
250K
98.9
200K
150K
100K
50K
0
3
3
4
5
-10
0
10
10
10
5
10
4
10
3
10
95.6
0
3
4
5
0
10
10
10
After sorting
Monocytes
Lineage
FSC-A
Ly6G
Ly6C
FSC-A
CD11b

## Slide 29
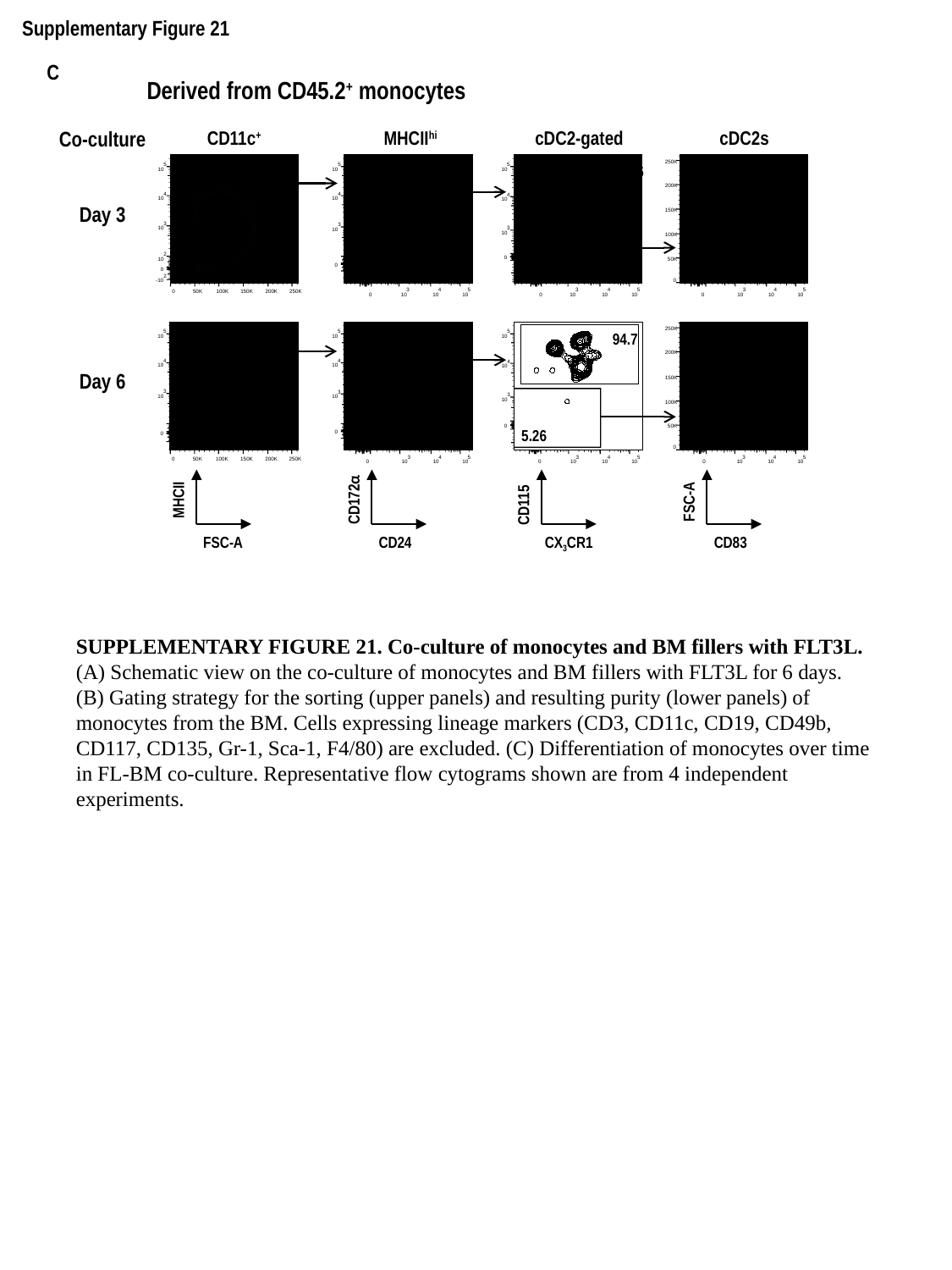

Supplementary Figure 21
C
Derived from CD45.2+ monocytes
Co-culture
Day 3
Day 6
CD11c+
MHCIIhi
cDC2-gated
cDC2s
5
9.17
10
4
10
3
76.5
10
2
10
10.6
0
2
-10
0
50K
100K
150K
200K
250K
5
10
98.6
4
10
3
10
1.17
0
3
4
5
0
10
10
10
5
94.6
10
4
10
3
10
0
4.26
3
4
5
0
10
10
10
250K
88.9
11.1
200K
150K
100K
50K
0
3
4
5
0
10
10
10
5
1.44
10
4
10
3
65.6
10
28.9
0
0
50K
100K
150K
200K
250K
5
10
76.0
4
10
3
10
0
0
3
4
5
0
10
10
10
5
94.7
10
4
10
3
10
0
5.26
3
4
5
0
10
10
10
250K
0
100
200K
150K
100K
50K
0
3
4
5
0
10
10
10
CD172α
CD24
MHCII
FSC-A
FSC-A
CD83
CD115
CX3CR1
SUPPLEMENTARY FIGURE 21. Co-culture of monocytes and BM fillers with FLT3L.
(A) Schematic view on the co-culture of monocytes and BM fillers with FLT3L for 6 days.
(B) Gating strategy for the sorting (upper panels) and resulting purity (lower panels) of monocytes from the BM. Cells expressing lineage markers (CD3, CD11c, CD19, CD49b, CD117, CD135, Gr-1, Sca-1, F4/80) are excluded. (C) Differentiation of monocytes over time in FL-BM co-culture. Representative flow cytograms shown are from 4 independent experiments.

## Slide 30
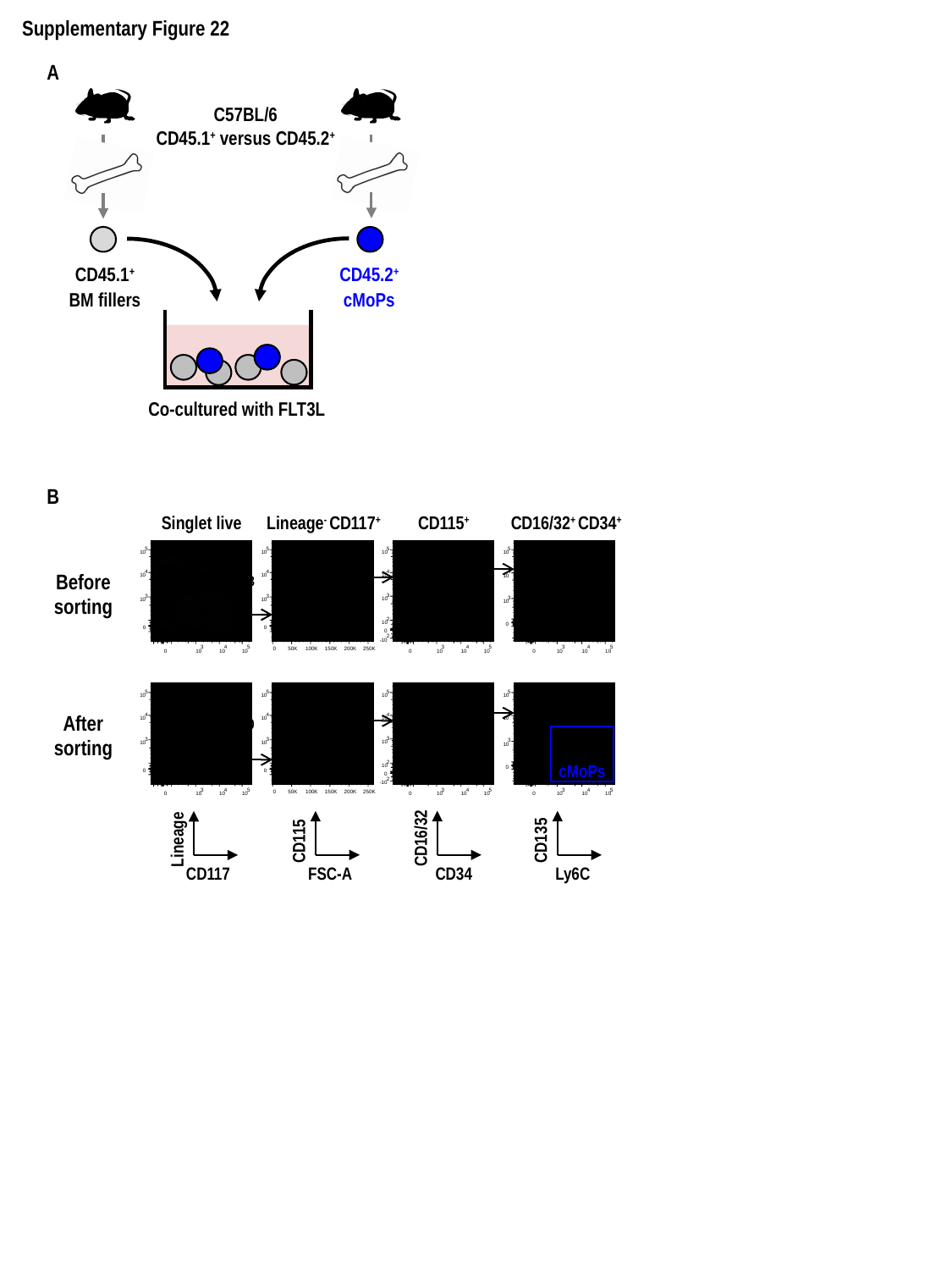

Supplementary Figure 22
A
C57BL/6
CD45.1+ versus CD45.2+
CD45.1+
BM fillers
CD45.2+
cMoPs
Co-cultured with FLT3L
B
Singlet live
Lineage- CD117+
CD115+
CD16/32+ CD34+
5
10
4
10
4.93
3
10
0
3
4
5
0
10
10
10
5
10
23.5
4
10
3
10
0
0
50K
100K
150K
200K
250K
5
56.5
10
4
10
3
10
2
10
0
2
-10
3
4
5
0
10
10
10
5
10
78.4
4
10
3
10
0
3
4
5
0
10
10
10
5
10
4
10
100
3
10
0
3
4
5
0
10
10
10
5
10
78.8
4
10
3
10
0
0
50K
100K
150K
200K
250K
5
97.8
10
4
10
3
10
2
10
0
2
-10
3
4
5
0
10
10
10
5
10
100
4
10
3
10
0
3
4
5
0
10
10
10
cMoPs
CD16/32
CD34
Lineage
CD117
CD135
Ly6C
CD115
FSC-A
Before sorting
After sorting

## Slide 31
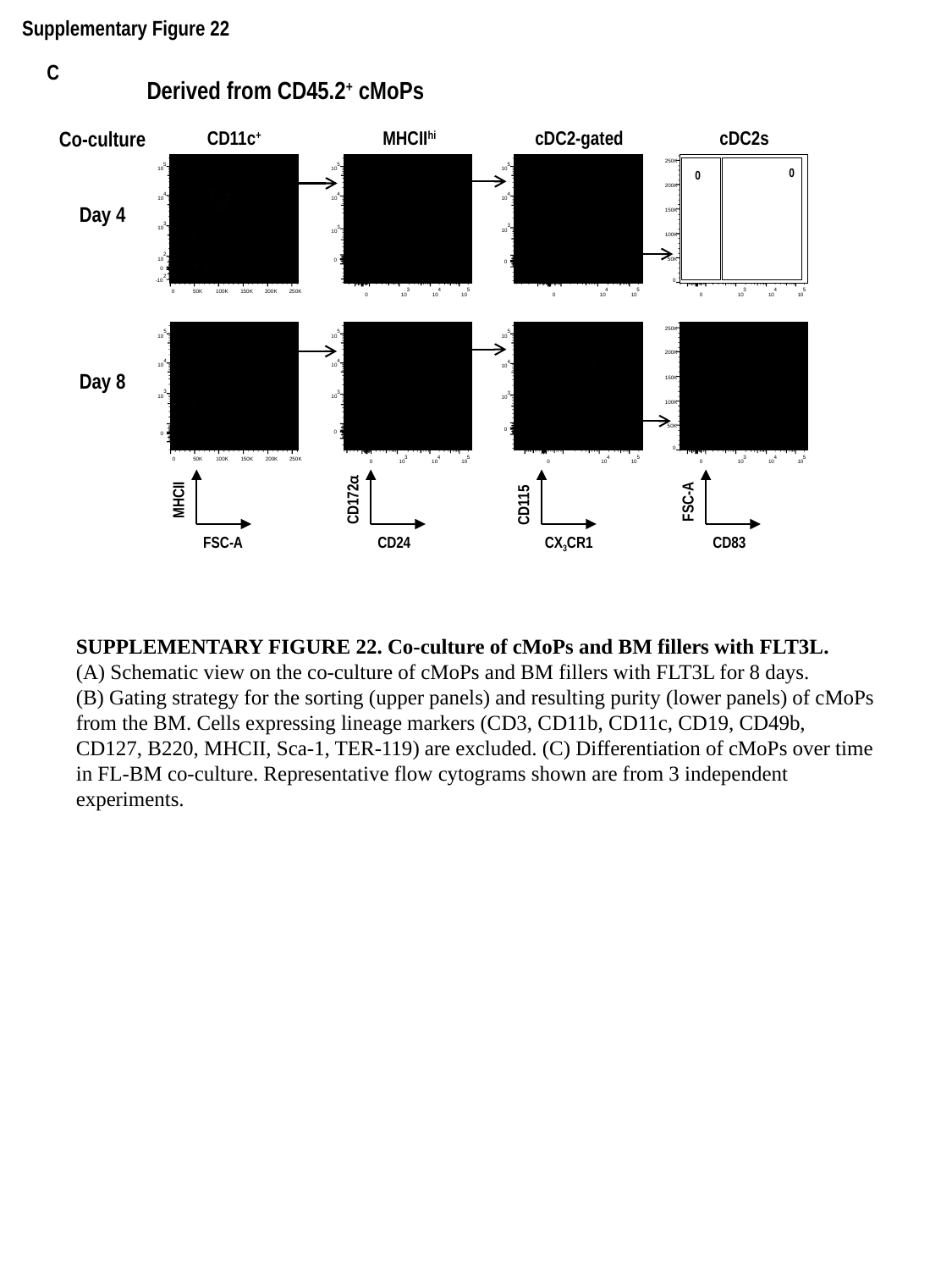

Supplementary Figure 22
C
Derived from CD45.2+ cMoPs
Co-culture
Day 4
Day 8
CD11c+
MHCIIhi
cDC2-gated
cDC2s
5
0.83
10
4
10
35.6
3
10
61.3
2
10
0
2
-10
0
50K
100K
150K
200K
250K
5
85.7
10
4
10
3
10
0
3
4
5
0
10
10
10
5
100
10
4
10
3
10
0
0
4
5
0
10
10
250K
0
0
200K
150K
100K
50K
0
3
4
5
0
10
10
10
5
1.67
10
4
10
36.0
3
10
61.0
0
0
50K
100K
150K
200K
250K
5
80.0
10
4
10
3
10
0
3
4
5
0
10
10
10
5
50.0
10
4
10
3
10
50.0
0
4
5
0
10
10
250K
100
0
200K
150K
100K
50K
0
3
4
5
0
10
10
10
CD172α
CD24
MHCII
FSC-A
FSC-A
CD83
CD115
CX3CR1
SUPPLEMENTARY FIGURE 22. Co-culture of cMoPs and BM fillers with FLT3L.
(A) Schematic view on the co-culture of cMoPs and BM fillers with FLT3L for 8 days.
(B) Gating strategy for the sorting (upper panels) and resulting purity (lower panels) of cMoPs from the BM. Cells expressing lineage markers (CD3, CD11b, CD11c, CD19, CD49b, CD127, B220, MHCII, Sca-1, TER-119) are excluded. (C) Differentiation of cMoPs over time in FL-BM co-culture. Representative flow cytograms shown are from 3 independent experiments.
